# Controllable Hydrosilylation and Dehydrogenative Silylation of Alkenes Catalyzed by a Manganese Alkyl Complex

**DOI:** 10.1021/acs.organomet.6c00169

**Published:** 2026-07-06

**Authors:** Daniel P. Zobernig, Luis F. Veiros, Karl Kirchner

**Affiliations:** † Institute of Applied Synthetic Chemistry, TU Wien, Getreidemarkt 9/163-AC, A-1060 Wien, Austria; ‡ Centro de Química Estrutural, Institute of Molecular Sciences, Departamento de Engenharia Química, Instituto Superior Técnico, 72971 Universidade de Lisboa, Avenida Rovisco Pais, 1049 001 Lisboa, Portugal

## Abstract

We report additive-free Mn­(I)-catalyzed controllable hydrosilylation (HS) and dehydrogenative silylation (DS) of terminal alkenes. The precatalyst is the well-defined Mn­(I) alkyl complex *fac*-[Mn­(PC-*i*Pr)­(CO)_3_(CH_2_CH_2_CH_3_)]. The reaction regioselectively yields either hydrosilylated alkenes or (*E*)-alkenyl silanes together with the corresponding alkanes in an approximately 1:1 ratio. The former are obtained selectively with primary and secondary silanes under neat conditions, while the latter are exclusively formed with more bulky tertiary silanes in THF as solvent. The reactions proceed with catalyst loadings of 1 mol % at 85 °C and a reaction time of 24 h. The catalytic process is initiated by migratory insertion of a CO ligand into the M*n*–alkyl bond to yield an acyl intermediate that undergoes Si–H bond cleavage of silane forming the active 16e^–^ Mn­(I) hydride complex [Mn­(PC-*i*Pr)­(CO)_2_(H)] together with a liberated siloxane Si–O–Si species. [Mn­(PC-*i*Pr)­(CO)_2_(H)] is the active catalyst in the DS cycle as well as the key intermediate for the formation of the silyl complex [Mn­(PC-*i*Pr)­(CO)_2_(silyl)], which is the key intermediate for the HS pathway. The DS pathway requires an alkene as a sacrificial hydrogen acceptor. Mechanistic insights are provided based on experimental data and DFT calculations.

## Graphical abstract

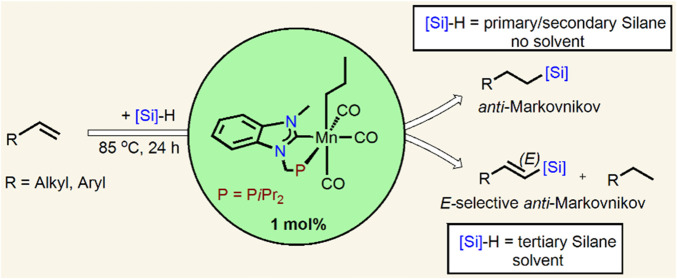

## Introduction

Catalytic functionalization of alkenes with hydrosilanes represents one of the most powerful methods for the synthesis of organosilicon molecules, which serve as essential building blocks in silicone polymers, electronic materials, and medicinal chemistry.^1–6^ These transformations of alkenes follow two distinct chemical trajectories: hydrosilylation (HS) and dehydrogenative silylation (DS). HS is an atom-economical addition of a Si–H bond across the alkene double bond. Current research focuses on achieving high *anti*-Markovnikov regioselectivity to produce linear alkyl silanes that are indispensable as precursors in materials science and as intermediates in organic synthesis. DS, conversely, yields unsaturated vinyl silanes or allyl silanes via a sacrificial alkene acceptor or through the formal loss of molecular hydrogen. This pathway is particularly prized in organic synthesis, as it installs a versatile silicon group while retaining a functionalizable double bond for subsequent cross-coupling or polymerization reactions.

While this field has historically relied on noble metals like platinum and rhodium (e.g., Speier’s,^7^ Karstedt’s,^8^ and Mark’o’s^9^ catalysts), the high cost and environmental impact of these elements have catalyzed a shift toward earth-abundant 3d transition metals^10–13^ such as Fe, Co, and Ni.^14–16^ In this context, also manganese complexes have recently become versatile and sustainable players in this field. Recent advancements in the ligand design ranging from pincer complexes to simple carbonyl precursors have enabled manganese systems to compete with their noble-metal counterparts.

An overview of well-defined Mn complexes for HS and DS of alkenes is shown in Scheme 1. One of the first reported manganese catalyst active for HS of alkenes was described in 2018 by Thomas and co-workers.^17^ They utilized a Mn­(II) NNN–pincer complex with two nitrogen donors and pyridine backbone as the central motif. This complex was able to quickly and efficiently hydrosilylate a wide variety of alkenes with NaOtBu as an additive. Trovitch and co-workers reported in the same year on a binuclear Mn­(II) complex with a NacNac-type ligand and bridging hydrides. It could hydrosilylate alkenes without any additives at 130 °C.^18^ Wang and co-workers demonstrated in 2018 that commercially available Mn­(CO)_5_Br can also be used at a catalyst loading of 5 mol %.^19^ In 2023, Ke and co-workers reported on a Mn­(II) complex bearing a tridentate trispyridino-ligand that could hydrosilylate alkenes at room temperature with NaOtBu as the base.^20^ In the same year, Thieuleux and co-workers utilized Mn­(CO)_5_Br and UV light to hydrosilylate a variety of alkenes with industrially relevant 1,1,1,3,5,5,5-heptamethyltrisiloxane.^21^ In 2025, Payard, Perrin, and co-workers utilized the Mn­(I) *n*-octyl complex [Mn­(CO)_5_(*n*-octyl)] for an *anti*-Markovnikov HS of alkenes at room temperature with loadings as low as 0.5 mol %.^22^

**Scheme 1:**
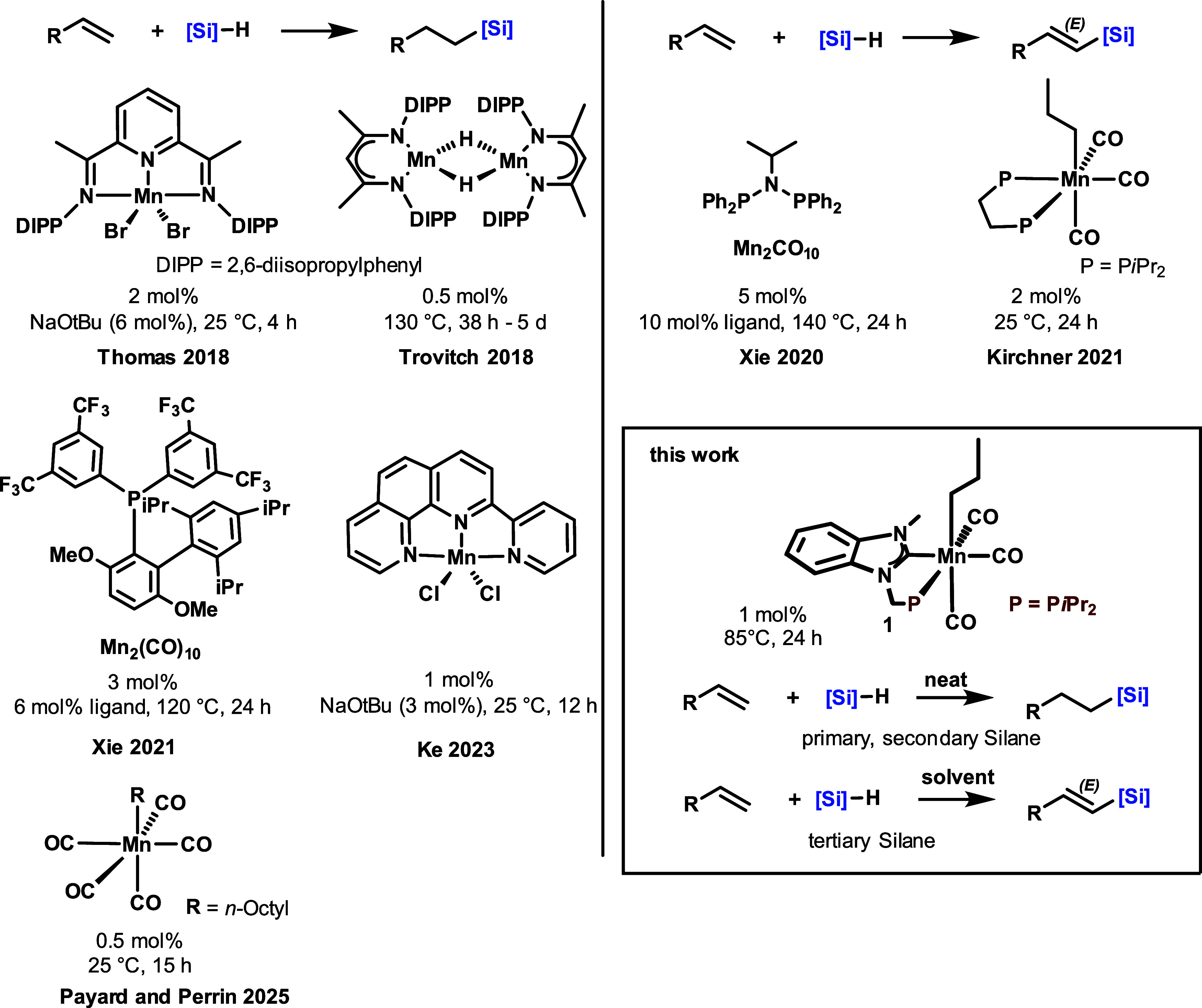
Overview of Manganese-Catalyzed Hydrosilylations (HS) and Dehydrogenative Silylations (DS) of Alkenes

The first reported DS with Mn was reported by Wang in 2018 who utilized Mn_2_(CO)_10_, albeit with a somewhat limited substrate scope and a high temperature of 150 °C.^19^ Xie and co-workers reported in 2020 on the efficient and selective dehydrogenative silylation of alkenes to yield the corresponding (*E*)-alkenyl silanes with 5 mol % Mn_2_CO_10_ and 10 mol % bidentate bisphosphine ligand.^23^ By changing the ligand to a bulky monodentate phosphine ligand, they could hydrosilylate alkenes, giving control over what product is obtained. The next year, we reported on the bench-stable alkyl bisphosphine Mn­(I) complex *fac-*[Mn­(dippe)­(CO)_3_(CH_2_CH_2_CH_3_)] (dippe = 1,2-bis­(di-*iso*-propylphosphino)­ethane)^24,25^ that is capable of converting a broad variety of aromatic and aliphatic alkenes efficiently and selectively into *E*-vinyl silanes and allyl silanes, respectively, with a catalyst loading of 2 mol % at room temperature.^26^

Here, we introduce the bench-stable Mn­(I) alkyl complex *fac*-[Mn­(PC-*i*Pr)­(CO)_3_(CH_2_CH_2_CH_3_)] (**1**)^27^ as the precatalyst for HS and DS of terminal alkenes to afford in a controllable fashion either alkyl silanes (*anti*-Markovnikov addition) or vinyl silanes (*E*-selective *anti*-Markovnikov). By simply changing the reaction medium (neat vs solvent) and the nature of the silane (primary and secondary vs tertiary silanes), we can flip the selectivity from nearly 100% HS to 100% DS.

## Results and Discussion

We studied the potential of Mn­(I) alkyl complex *fac*-[Mn­(PC-*i*Pr)­(CO)_3_(CH_2_CH_2_CH_3_)] (**1**) as the precatalyst for the HS of 4-chlorostyrene as model substrate using various primary, secondary, and tertiary silanes (1.1 equiv). Optimization reactions are depicted in Table 1. At 60 °C under neat conditions with SiH_2_MePh and a catalyst loading of 1 mol %, a conversion of 60% was achieved affording HS product **2**, DS product **3**, and 1-chloro-4-ethylbenzene **4** in a ratio of 97/2/1 (Table 1, entry 1). When performing the reaction in THF, toluene and 1,2-DCE, the conversion dropped significantly to 20, 11, and 5%, respectively (Table 1, entries 2–4). The silanes SiH_2_Ph_2_, SiH_3_Ph, and SiHMe_2_Ph were then also tested under neat conditions. The first two silanes were selectively converted to HS **2**, but with conversions of only 49 and 59%, respectively (Table 1, entries 5 and 6). The reaction with the tertiary silane SiHMe_2_Ph was essentially quantitative but not very selective, forming the corresponding compounds **2**, **3**, and **4** in a 64/28/8 ratio (Table 1, entry 7). Upon increasing the temperature to 85 °C with SiH_2_MePh, alkyl silane **2** was obtained in an essentially quantitative yield (Table 1, entry 8).

**Table 1:**
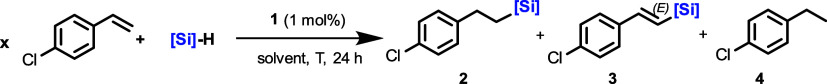
Optimization Reactions for the HS and DS of 4-Chlorostyrene with Silanes with **1** as the Precatalyst^a^

entry	silane ([Si])	*x* (equiv)	solvent	*T* [°C]	conversion [%]	**2/3/4**
**1**	SiH_2_MePh	1	neat	60	66	97/2/1
**2**	SiH_2_MePh	1	THF	60	20	87/10/3
**3**	SiH_2_MePh	1	toluene	60	11	78/14/8
**4**	SiH_2_MePh	1	1,2-DCE	60	5	55/30/15
**5**	SiH_2_Ph_2_	1	neat	60	49	100/–/–
**6**	SiPhH_3_	1	neat	60	57	100/–/–
**7**	SiHMe_2_Ph	1	neat	60	>99	64/28/8
**8**	**SiH**_**2**_ **MePh**	**1**	**neat**	**85**	**>99**	**100/–/–**
**9**	**SiHMe**_**2**_ **Ph** ^b^	**1.8**	**THF**	**85**	**99**	**0/60/40**

a Reaction conditions: 4-Chlorostyrene (27 μL, 0.23 mmol, 1 equiv), silane (0.25 mmol, 1.1 equiv), **1** (2.3 μmol, 1 mol %), 0.5 mL of solvent, 60–85 °C, 24 h, conversion and isomer ratio determined by GC-MS.

b Silane (0.41 mmol, 1.8 equiv).

Since the tertiary silane SiHMe_2_Ph afforded substantial amounts of DS product **2** (Table 1, entry 7), this silane was reacted in THF as the solvent at 85 °C but with an excess of 4-chlorostyrene (1.8 equiv) resulting in the full conversion of the alkene to yield (*E*)-vinyl silane **3** together with hydrogenated product 1-chloro-4-ethylbenzene (**4**) in a 60:40 ratio (Table 1, entry 9). It has to be noted that in the absence of an additional acceptorless DS pathway, which is accompanied by the release of dihydrogen, the ratio of **3**:**4** should be typically 50:50. Accordingly, to some extent, also this pathway seems to take place.^24^ Finally, no reaction took place without any catalyst.

With the optimized conditions in hand, the scope and limitations were examined as depicted in Table 2. SiH_2_MePh, SiH_3_Ph, and SiH_2_Ph_2_ were reacted with styrene forming the respective alkyl silanes (**2a**, **2c**) in good to excellent yields. Styrene derivatives with both electron-donating groups such as *tert*-butyl and methoxy and electron-withdrawing groups such as chloro and trifluoromethyl in the *para*-position were well-tolerated, resulting in excellent to quantitative conversions to the corresponding alkyl silanes with very good selectivities (**2d**–**2g**). Substituents in the *meta*- and *ortho*-positions as well as strongly electron-poor alkenes such as 2,3,4,5,6-pentafluorostyrene did not significantly alter the conversions and selectivities (**2h**–**2j**). 2-Vinylnaphthalene and 4-vinylphenyl acetate could also successfully be hydrosilylated, with no reaction taking place at the ester moiety for the latter (**2k** and **2l**). Aliphatic alkenes were also examined and were chemoselectively hydrosilylated (**2m**, **2n**). 4-Phenyl-1-butene was completely converted into methylphenyl­(4-phenylbut-1-yl)­silane (**2o**) albeit with a slightly poorer selectivity, which may be attributed to isomerization reactions. This effect can also be observed for allyl substrates such as allylbenzene and allyltrimethylsilane, which exhibited only moderate conversions (**2p**, **2q**). Finally, a 1,1-disubstituted alkene was also hydrosilylated, but dimethylphenylsilane had to be used, as no reaction took place otherwise (**2r**).

**Table 2:**
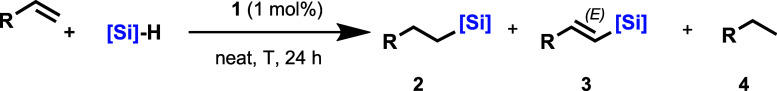

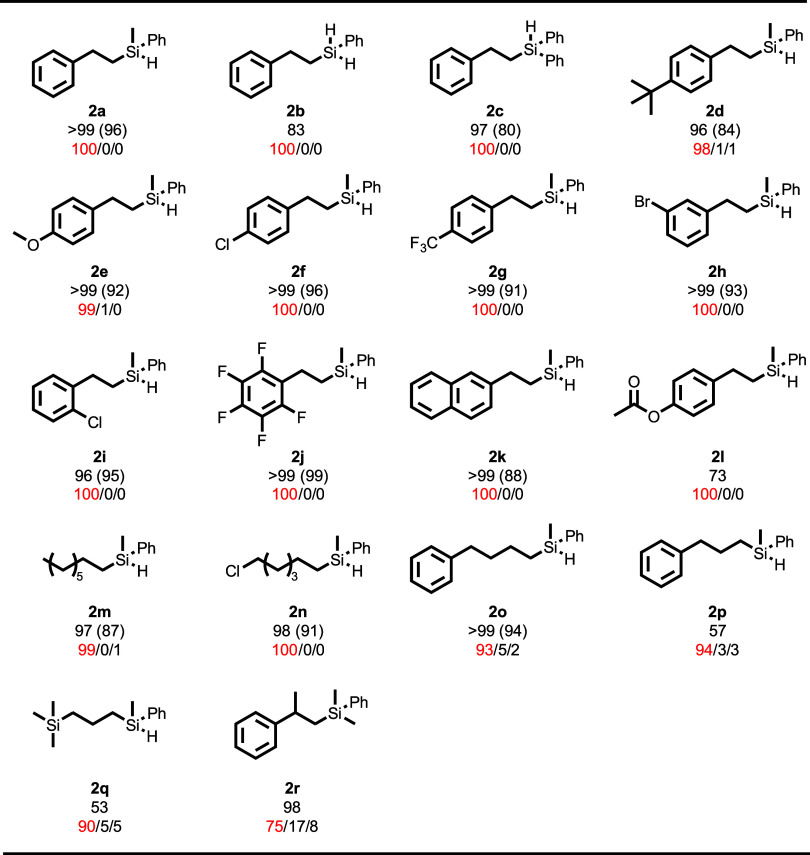
HS of Terminal Alkenes Catalyzed by **1** ^a^

a Reaction conditions: alkene (0.50 mmol, 1.0 equiv), silane (0.55, 1.1 equiv), **1** (1 mol %), 85 °C, 24 h, conversion and product ratio determined by GC-MS, isolated yield in parentheses, product ratio is given as **2**/**3**/**4**.

Furthermore, we examined the scope and limitations of the DS of terminal alkenes, as shown in Table 3. Styrene as well as styrene derivatives with electron-donating groups such as *tert*-butyl and methoxy in the *para*-position were fully converted to the corresponding (*E*)-alkenyl silanes (**3a**–**3c**). Electron-withdrawing groups in *para*- and *meta*-positions were also well-tolerated, resulting in mostly full conversions (**3d**, **3e**) with excellent selectivities. Moreover, also a very electron-rich system such as 2,4,6-trimethylstyrene and N-vinylcarbazole could be silylated successfully converted to **3f** and **3g**. 1,1-Disubstituted alkenes reacted with the corresponding vinyl silanes, but due to isomerization reactions, a mixture of two products was obtained in a ratio of 67:33 (**3h**, **3i**). Aliphatic alkenes were silylated, albeit not selectively, and a mixture of different isomers was obtained (**3j**).

**Table 3:**
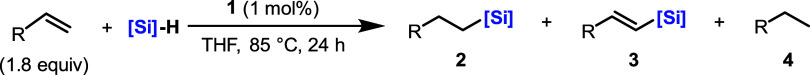

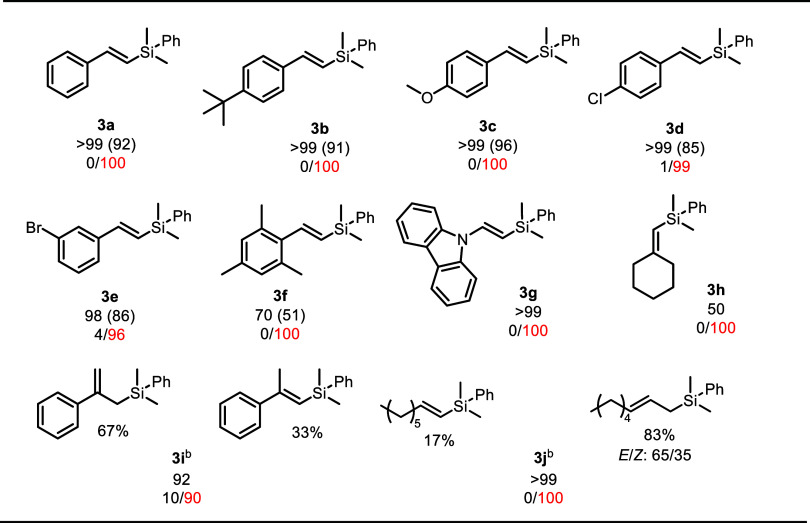
DS of Terminal Alkenes Catalyzed by **1** ^a^

a Reaction conditions: Me_2_PhSiH (77 μL, 0.50 mmol, 1 equiv), alkene (0.8 mmol, 1.8 equiv), **1** (1 mol %), 1 mL of THF, 85 °C, 24 h, conversion and product ratio determined by GC-MS, isolated yield in parentheses, product ratio given below as **2**/**3**, >98% (*E*)-isomer detected unless stated otherwise.

b The product ratio was determined by NMR spectroscopy.

The homogeneity of the reaction was confirmed by addition of one drop of mercury, where no decrease of reactivity and selectivity was observed for the HS and DS of 4-chlorostyrene with SiH_2_MePh and SiHMe_2_Ph, respectively. In the presence of 1 equiv of PEt_3_ (with respect to substrate), only traces of product formation could be detected, which indicates an inner-sphere mechanism due to coordination of PEt_3_ blocking a vacant side of the active species for incoming substrates.

In order to get further mechanistic insights, several additional experiments were carried out. Upon reacting **1** with SiHMe_2_Ph (3 equiv) in THF-*d*_8_ at 50 °C for 6 h, a new compound was detected: NMR spectroscopy giving rise to two doublets centered at −8.86 (1H) and −8.82 (1H) ppm in the ^1^H NMR spectrum assignable to two hydridic hydrogen atoms, a singlet at 124.9 ppm in the ^31^P­{^1^H} NMR spectrum, and a signal at −3.3 ppm in the ^29^Si­{^1^H} NMR spectrum (see Figures S1–S3). This species, which could not be isolated, was tentatively assigned to [Mn­(PC-*i*Pr)­(CO)_2_(κ^2^ *H,H*-H_2_SiMe_2_Ph)] (**4**), featuring presumably a κ^2^ *H,H*-bound silane (Scheme 2). The chemical shifts are in the same region as described by Schubert and co-workers for the manganese complex [Mn­(CpMe)­(CO_2_)­(H-SiR_2_SiR_2_H)] featuring an agostic Si–H bond.^28^ In addition, under these conditions, the formation of the siloxane PhMe_2_Si–O–SiMe_2_Ph was detected by GC/MS (*m*/*z*: 286.13 [M^+^]) (see Figure S4). This compound originates from the addition of the strongly electrophilic silane moiety to the oxygen atom of an acyl intermediate (see Figure S5). In total, the formation of **4** and PhMe_2_Si–O–SiMe_2_Ph requires 3 equiv of silane. Thus, the source of oxygen in the siloxane is the oxygen atom of one CO ligand. In fact, in the absence of oxygen (air) no siloxanes were detected. These complex catalyst initiation processes are currently the subject of extensive computational and experimental investigations and will be reported in detail in a future publication.

**Scheme 2:**
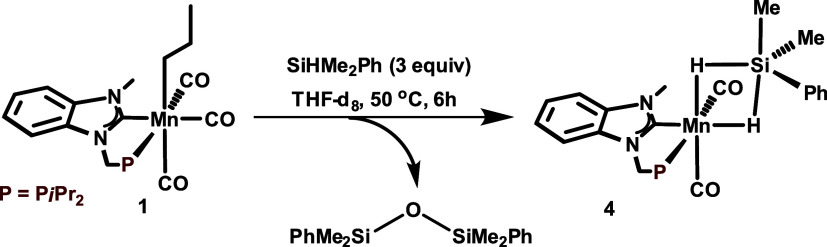
Reaction of **1** with SiHMe_2_Ph in THF-*d*_8_

When styrene-*d*_8_ reacts with SiH_2_MePh under neat conditions (Scheme 3), hydrogen is incorporated predominantly into the benzylic carbon atom. This selectivity agrees reasonably well with the catalytic cycle shown in Scheme 4 (from **VI** to **VII**, H-transfer from the silane to the alkyl moiety takes place, thereby releasing the product) but also to a lesser extent at the carbon adjacent to Si, which we cannot explain.

**Scheme 3:**
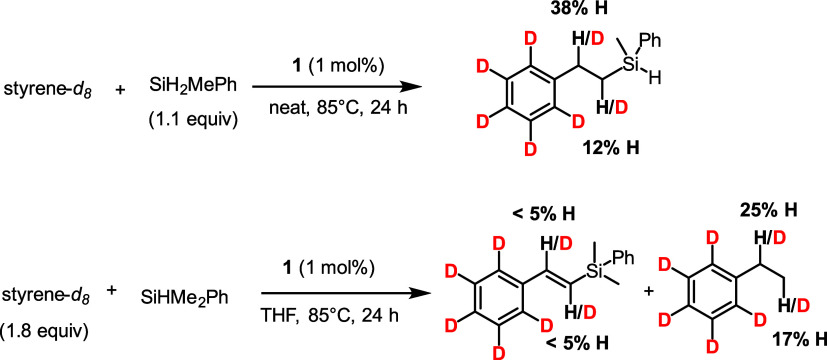
HS and DS of Styrene-*d*_8_ ^a^

a Reaction conditions: Styrene-*d*_8_ (54–97 μL, 0.46–0.83 mmol, 1.1–1.8 equiv), silane (0.46–0.50 mmol, 1.1–1.1 equiv), 1 mL of solvent, ^1^H and ^2^H-incorporation determined by ^1^H and ^2^H NMR spectroscopy.

**Scheme 4:**
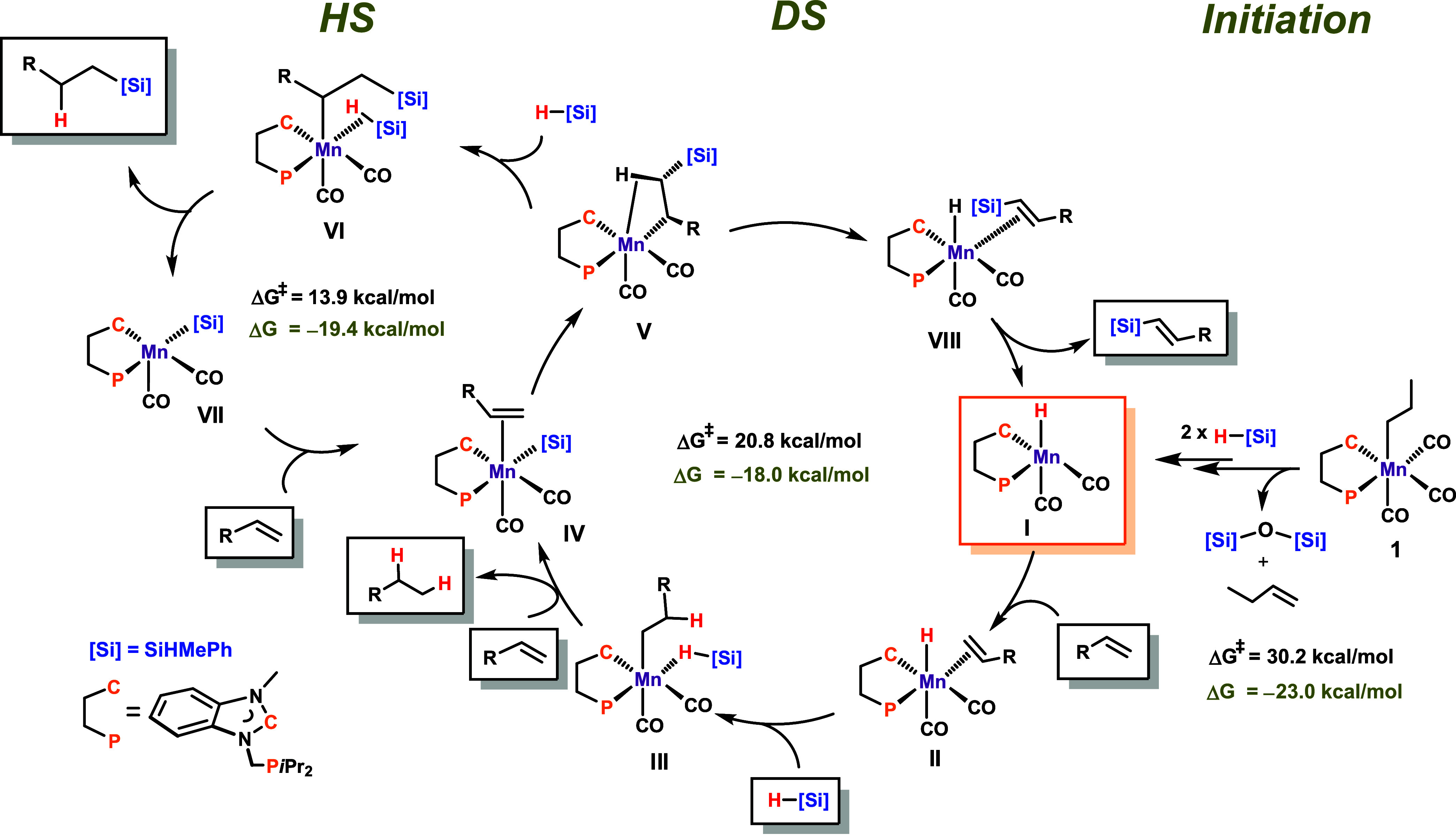
Proposed Mechanism for the HS and DS Pathways

If styrene-*d*_8_ was used as a substrate in combination with SiHMe_2_Ph (1.8 equiv) in THF, only traces of hydrogen were found in the DS product, but as expected, higher levels of hydrogen were found at the internal and, to a lesser extent, also at the terminal carbon atoms of ethylbenzene. This is expected for the DS pathway as the H distribution depends on the orientation of the coordinated alkene with respect to hydride in **II** (in Scheme 4, only one orientation is depicted).

Noteworthy, when performing the DS in an open system, the product ratio shifts toward the DS product. With an essentially quantitative conversion of 4-chlorostyrene, 76% of silylated 4-chlorostyrene (**3d**) and 24% of 1-chloro-4-ethylbenzene (**4d**) were formed. Accordingly, under these conditions, an acceptorless dehydrogenation pathway comes into play, leading to an increase of alkyl silane with respect to the alkane. This alternative pathway was recently also described in detail for the DS of alkenes with *fac-*[Mn­(dippe)­(CO)_3_(CH_2_CH_2_CH_3_)] as the precatalyst also under neat conditions with tertiary silanes albeit already at room temperature.^26^

The mechanism of the HS and DS of terminal alkenes catalyzed by **1** was also investigated in detail by DFT calculations^29^ using 1-butene and SiH_2_MePh as model substrates (see the Supporting Information for computational details). In Scheme 4, simplified catalytic cycles are depicted showing only key intermediates. Free energy profiles are provided in Figures S6–S12.

Catalyst initiation starts from **1**, involving migratory insertion of the propyl ligand into a Mn–CO bond to form an acyl species intermediate,^24–27^ which, then, reacts with two molecules of silane to form the active 16e^–^ hydride complex [Mn­(PC-*i*Pr)­(CO)_2_(H)] (**I**) together with siloxane PhMe_2_Si–O–SiMe_2_Ph and 1-butene.

The catalytic cycles start with butene coordination to hydride **I** forming intermediate **II** (Scheme 4). From here, migratory insertion of the hydride into the C=C double bond yields an alkyl complex that coordinates silane resulting in the Si–H complex that corresponds to intermediate **III**, in Scheme 4. From **III**, H-transfer from the κ^2^-(Si,H)-silane to the alkyl releases butane and forms a silyl species that coordinates a fresh butene to yield intermediate **IV**. This process, from **I** to **IV**, has a favorable thermodynamic balance of Δ*G* = – 27.1 kcal/mol and a barrier of 6.9 kcal/mol, associated with the H-transfer step, from the silane ligand to the alkyl. The initial part of the catalytic process, from **I** to **IV**, is depicted in the profiles of Figures S6 and S7.

In **IV**, the rearrangement of the η^2^-olefin ligand is followed by Si–C bond formation through migration of the Si-atom to the terminal C atom of butene yielding intermediate **V**, a complex with a coordinated silyl alkyl ligand. This is the point where the HS and the DS cycles diverge. In intermediate **V**, β-elimination provides a silylated olefin ligand continuing in the DS cycle, through intermediate **VIII**. Conversely, the HS cycle proceeds, from **V**, through addition of a fresh silane molecule and formation of intermediate **VI**.

In intermediate **VI**, H-transfer from the silane to the silylated alkyl ligand releases the HS product, CH_3_(CH_2_)_3_SiHMePh, and forms silyl intermediate **VII**. The HS cycle proceeds, from **VII**, through addition of a fresh butane molecule and regeneration of the silyl alkene intermediate **IV**. The free energy balance for the HS cycle is rather favorable (Δ*G* = −19.4 kcal/mol) and the overall barrier is 13.9 kcal/mol, measured from the olefin/silyl intermediate **IV** to the transition state associated with the Si–C bond formation. The HS cycle is represented in Figures S8–S10 and the relevant points to the barrier are intermediate **I** (Figure S8) and transition state **TS**_**JK**_ (Figure S9).

The first part of the DS catalytic cycle, from **I** to **V**, was described above and is represented in the profiles of Figures S6 and S7. From **V**, there is a rearrangement of the silylated alkyl ligand providing a C–H agostic interaction that is followed by β-elimination of the H atom involved in that interaction. This yields intermediate **VIII** with a coordinated silylated olefin that corresponds to the DS product. Release of this olefin (CH_3_CH_2_CH=CHSiHMePh) from **VIII** regenerates hydride **I** and closes the cycle. The DS cycle has a free energy balance of Δ*G* = −18.0 kcal/mol and an overall barrier of 20.8 kcal/mol, measured from the silyl alkyl intermediate, **V**, to the following transition state for addition of butene to the hydride complex. The DS cycle is represented in Figures S6–S9, S11, and S12, and the relevant points to the barrier are intermediate **O** (Figure S7) and transition state **TS**_**AB**_ (Figure S2).

Importantly, the calculated mechanism indicates a more facile HS process, with a barrier 6.9 kcal/mol lower than the one obtained for the DS cycle, in good agreement with the experimental observations for the silane used in the calculations (SiH_2_MePh). Moreover, the relevant step in the determination of the barrier for the HS catalytic cycle is the one corresponding to migration of the silyl ligand to the terminal C=C carbon atom in the adjacent olefin. Thus, bulkier tertiary silanes should disfavor this process, increasing the corresponding barrier and shifting the reaction toward the DS cycle. This was recently also observed with [Mn­(dippe)­(CO)_3_(CH_2_CH_2_CH_3_)] as the catalyst utilizing tertiary silanes.^26^

## Conclusion

HS and DS of alkenes display an interesting approach to synthesize alkyl silanes and vinyl silanes, respectively. We have established a solvent-free manganese-catalyzed HS procedure of terminal alkenes with no additives needed and a catalyst loading of 1 mol % at 85 °C. The is the bench-stable alkyl Mn­(I) complex *fac*-[Mn­(PC-*i*Pr)­(CO)_3_(CH_2_CH_2_CH_3_)] is the precatalyst for HS and DS of a series of terminal alkenes to afford in a controllable fashion either alkyl silanes (*anti*-Markovnikov addition) or vinyl silanes (*E*-selective *anti*-Markovnikov). By simply changing the reaction media (neat vs solvent (THF)) and the nature of the silane (primary and secondary vs tertiary silanes), we can flip the selectivity from nearly 100% HS to 100% DS. The catalytic process is initiated by migratory insertion of a CO ligand into the M*n*–alkyl bond to yield an acyl intermediate that undergoes rapid Si–H bond cleavage of the silane HSiR_3_ forming, after several rearrangement steps, the active 16e^–^ hydride complex [Mn­(PC-*i*Pr)­(CO)_2_(H)]. This is also the intermediate for the silyl complex [Mn­(PC-*i*Pr)­(CO)_2_(silyl)] that is the key intermediate for the HS pathway. In the case of DS, one molecule of alkene acts as a sacrificial hydrogen acceptor and thus, the ratio of vinyl silane or allyl silane to alkane approaches a 1:1 ratio as normally observed. In contrast to several other manganese-based procedures, the reaction proceeds via a classical inner-sphere mechanism rather than via radical routes.

Mechanistic studies that include *in situ* NMR measurements, stoichiometric reactions, and deuterium labeling experiments in addition to computational investigations provided insights into the reaction mechanism. The DFT-calculated mechanism indicates a more facile HS process with a barrier 6.9 kcal/mol lower than the one obtained for the DS cycle, which is in excellent agreement with the experimental observations for the silane used in the calculations (SiH_2_MePh). Moreover, the relevant step in the determination of the barrier for the HS catalytic cycle is the one corresponding to migration of the silyl ligand to the terminal C=C carbon atom in the adjacent olefin. Thus, it is expected that bulkier tertiary silanes would unfavor this process and increase the corresponding barrier, shifting the reaction toward the DS cycle, as indeed observed.

## Materials and Methods

### General Procedure for Hydrosilylation of Alkenes

Inside an argon-flushed glovebox, a screw cap vial (8 mL) was sequentially charged with **1** ^27^ (2.2 mg, 1 mol %), alkene (0.5 mmol, 1 equiv), and silane (0.55 mmol, 1.1 equiv). A stirring bar was added, the vial was closed, transferred out of the glovebox, and was stirred for 24 h at 85 °C. Afterward, the reaction mixture was allowed to reach room temperature and exposed to air to quench the catalyst. 2 μL of the sample was analyzed via GC-MS. The solvent was subsequently removed in *vacuo* and the crude product was filtered through a thin pad of silica with the given solvent. The product was then dried *in vacuo*, after which it was characterized with ^1^H-, ^13^C­{^1^H}-, and ^29^Si­{^1^H}-NMR spectroscopy.

### General Procedure for Dehydrogenative Silylation of Alkenes

Inside an argon-flushed glovebox, a screw cap vial (8 mL) was sequentially charged with **1** (2.2 mg, 1 mol %), alkene (0.9 mmol, 1.8 equiv), SiHMe_2_Ph (77 μL, 0.5 mmol, 1.1 equiv), and THF (1 mL). A stirring bar was added, the vial was closed, transferred out of the glovebox, and was stirred for 24 h at 85 °C. Afterward, the reaction mixture was allowed to reach room temperature and exposed to air to quench the catalyst. 2 μL of the sample was analyzed via GC-MS. The solvent was subsequently removed *in vacuo* and the crude product was purified via column chromatography with the given solvent. The product was then dried *in vacuo*, after which it was characterized with ^1^H-, ^13^C­{^1^H}-, and ^29^Si­{^1^H}-NMR spectroscopy.

## Supplementary Material





## Notes

Cartesian coordinates for DFT-optimized structures (XYZ)
Synthetic procedures, ^1^H, ^13^C­{^1^H}, and ^31^P­{H} NMR spectra of all compounds and complete computational details (PDF)
