# Supplementary material for: Controllable Hydrosilylation and Dehydrogenative Silylation of Alkenes Catalyzed by a Manganese Alkyl Complex
Source: Organometallics. 2026 Jul 6;45(14):1731–9. doi: 10.1021/acs.organomet.6c00169 (PMC13418219; doi:10.1021/acs.organomet.6c00169)
Supplement: Supplementary file 2 [file om6c00169_si_002.pdf]

# Controllable Hydrosilylation and Dehydrogenative Silylation of Alkenes Catalyzed by a Manganese Alkyl Complex

**Daniel P. Zobernig,[†] Luis F. Veiros,[$] and Karl Kirchner*,[†]**

[†] Institute of Applied Synthetic Chemistry, TU Wien, Getreidemarkt 9/163-AC, A-1060 Wien, Austria.

e-mail: karl.kirchner@tuwien.ac.at

[$] Centro de Química Estrutural, Institute of Molecular Sciences, Departamento de Engenharia Química, Instituto Superior Técnico, Universidade de Lisboa, Av. Rovisco Pais, 1049 001 Lisboa, Portugal

## Supporting Information

Experimental Section ........ 2

General Information. ........ 2

General Procedure for Hydrosilylation of Alkenes. ........ 2

General Procedure for Dehydrogenative Silylation of Alkenes ........ 2

Mechanistic Studies ........ 2

Deuteration Experiment with Styrene-$d_8$ and Methylphenylsilane. ........ 2

Deuteration Experiment with Styrene-$d_8$ and Dimethylphenylsilane. ........ 2

Poisoning Experiment with Mercury. ........ 3

Poisoning Experiment with $PEt_3$. ........ 3

Dehydrogenative Silylation in an Open System. ........ 3

Reaction of 1 with Dimethylphenylsilane. ........ 3

Computational Details ........ 5

Characterization of Organic Products ........ 9

References ........ 14

NMR Spectra ........ 16

# Experimental Section

**General Information.** All reactions were performed under an inert atmosphere of argon by using Schlenk techniques or in a MBraun inert-gas glovebox. The solvents were purified according to standard procedures. All substrates were purchased from Sigma-Aldrich, Acros Organics, TCI or BLDpharm and used as purchased without further purification. The deuterated solvents were purchased from Eurisotope and dried over 3 Å molecular sieves. $^{1}H$, $^{13}C\{^{1}H\}$, $^{29}Si\{^{1}H\}$ and $^{31}P\{^{1}H\}$ NMR spectra were recorded on Bruker AVANCE-400, AVANCE-NEO-400 and AVANCE-600 spectrometers. $^{1}H$ and $^{13}C\{^{1}H\}$ NMR spectra were referenced internally to residual protio-solvent, and solvent resonances, respectively, and are reported relative to tetramethylsilane ($\delta$ = 0 ppm). $^{29}Si\{^{1}H\}$ NMR spectra were referenced externally to tetramethylsilane ($\delta$ = 0 ppm). $^{31}P\{^{1}H\}$ NMR spectra were referenced externally to $H_3PO_4$ (85%) ($\delta$ = 0 ppm). Preparative flash column chromatography was conducted manually using glass columns packed with silica gel 60 (Merck, 40-63 µm). Complex *fac*-[Mn(PC-*i*Pr)(CO)$_3$(CH$_2$CH$_2$CH$_3$)] (**1**) was synthesiszed according to literature.[1–3]

High resolution-accurate mass data mass spectra were recorded on a hybrid Maxis Qq-aoTOF mass spectrometer (Bruker Daltonics, Bremen, Germany) fitted with an ESI-source. Measured accurate mass data of the $[M]^+$ ions for confirming calculated elemental compositions were typically within ±5 ppm accuracy. The mass calibration was done with a commercial mixture of perfluorinated trialkyl-triazines (ES Tuning Mix, Agilent Technologies, Santa Clara, CA, USA).

GC–MS analyses were conducted on a ISQ LT Single quadrupole MS (Thermo Fisher) directly interfaced to a TRACE 1300 Gas Chromatographic systems (Thermo Fisher), using a Rxi-5Sil MS (30 m, 0.25mm ID) cross-bonded dimethyl polysiloxane capillary column at a carrier flow of He 1.5 mL/min.

**General Procedure for Hydrosilylation of Alkenes.** Inside an argon flushed glovebox, a screw cap vial (8 mL) was charged with **1** (2.2 mg, 1 mol%), alkene (0.5 mmol, 1 equiv.) and silane (0.55 mmol, 1.1 equiv.) in this order. A stirring bar was added, the vial was closed, transferred out of the glovebox and was stirred for 24 hours at 85 °C. Afterwards the reaction mixture was allowed to reach room temperature and exposed to air to quench the catalyst. 2 µL of the sample was analyzed *via* GC-MS. The solvent was subsequently removed in *vacuo* and the crude product was filtered through a thin pad of *silica* with given solvent. The product was then dried in *vacuo*, after which it was characterized with $^{1}H$-, $^{13}C\{^{1}H\}$- and $^{29}Si\{^{1}H\}$ NMR spectroscopy.

**General Procedure for Dehydrogenative Silylation of Alkenes.** Inside an argon flushed glovebox, a screw cap vial (8 mL) was charged with **1** (2.2 mg, 1 mol%), alkene (0.9 mmol, 1.8 equiv.), dimethylphenylsilane (77 µL, 0.5 mmol, 1.1 equiv.) and THF (1 ml) in this order. A stirring bar was added, the vial was closed, transferred out of the glovebox and was stirred for 24 hours at 85 °C. Afterwards the reaction mixture was allowed to reach room temperature and exposed to air to quench the catalyst. 2 µL of the sample was analyzed *via* GC-MS. The solvent was subsequently removed in *vacuo* and the crude product was purified *via* column chromatography with given solvent. The product was then dried in *vacuo*, after which it was characterized with $^{1}H$-, $^{13}C\{^{1}H\}$- and $^{29}Si\{^{1}H\}$ NMR spectroscopy.

# Mechanistic Studies

**Deuteration Experiment with Styrene-d$_8$ and Methylphenylsilane.** Inside an argon flushed glovebox, a screw cap vial (8 mL) was charged with **1** (2.2 mg, 1 mol%), styrene-d$_8$ (57 µL, 0.5 mmol, 1 equiv.) and methylphenylsilane (76 µL, 0.55 mmol, 1.1 equiv.) in this order. A stirring bar was added, the vial was closed, transferred out of the glovebox and was stirred for 24 hours at 85 °C. Afterwards the reaction mixture was allowed to reach room temperature and exposed to air to quench the catalyst. The reaction mixture was characterized with $^{1}H$- and $^{2}H$-NMR spectroscopy to determine the hydrogen and deuterium incorporation.

**Deuteration Experiment with Styrene-d$_8$ and Dimethylphenylsilane.** Inside an argon flushed glovebox, a screw cap vial (8 mL) was charged with **1** (2.2 mg, 1 mol%), styrene-d$_8$ (104 µL, 0.9 mmol, 1.8 equiv.), dimethylphenylsilane (77 µL, 0.5 mmol, 1.0 equiv.) and THF (1 mL) in this order. A stirring-bar was added, the vial was closed, transferred out of the glovebox and was stirred for 24 hours at 85 °C. Afterwards the reaction mixture was allowed to reach room temperature and exposed to air to quench the catalyst. The reaction mixture was characterized with $^{1}H$- and $^{2}H$-NMR spectroscopy to determine the hydrogen and deuterium incorporation.

**Poisoning Experiment with Mercury.** Inside an argon flushed glovebox, a screw cap vial (8 mL) was charged **1** (1 mg, 1 mol%), 4-chlorostyrene (27 µL, 0.23 mmol, 1.0 equiv.) and methylphenylsilane (34 µL, 0.25 mmol, 1.1 equiv.) and a drop of mercury in this order. A stirring-bar was added, the vial was closed, transferred out of the glovebox and was stirred for 24 hours at 85 °C. Afterwards the reaction mixture was allowed to reach room temperature and exposed to air to quench the catalyst. 2 µL of the sample was analyzed *via* GC-MS. A conversion of 85% was detected.

**Poisoning Experiment with $PEt_3$.** Inside an argon flushed glovebox, a screw cap vial (8 mL) was charged **1** (1 mg, 1 mol%), 4-chlorostyrene (27 µL, 0.23 mmol, 1.0 equiv.) methylphenylsilane (34 µL, 0.25 mmol, 1.1 equiv.) and $PEt_3$ (34 µL, 0.23 mmol, 1.0 equiv.) in this order. A stirring-bar was added, the vial was closed, transferred out of the glovebox and was stirred for 24 hours at 85 °C. Afterwards the reaction mixture was allowed to reach room temperature and exposed to air to quench the catalyst. 2 µL of the sample was analyzed *via* GC-MS. A conversion of less than 1% was detected.

**Dehydrogenative Silylation in an Open System.** Inside an argon flushed glovebox, a Schlenk flask (15 mL) was charged **1** (1 mg, 1 mol%), 4-chlorostyrene (27 µL, 0.23 mmol, 1.0 equiv.), dimethylphenylsilane (35 µL, 0.23 mmol, 1.0 equiv.) and 2-methyltetrahydrofuran (0.5 mL) in this order. A stirring-bar was added, the flask was closed and transferred out of the glovebox. The flask was then opened to the Schlenk line to ensure an inert atmosphere and stirred for 24 h at 85 °C. Afterwards the reaction mixture was allowed to reach room temperature and exposed to air to quench the catalyst. 2 µL of the sample was analyzed *via* GC-MS. A conversion of over 99% of 4-chlorostyrene was detected, of which 76% was silylated 4-chlorostyrene and 24% was 1-chloro-4-ethylbenzene.

**Reaction of 1 with Dimethylphenylsilane.** Inside an argon flushed glovebox, an NMR-tube was charged with **1** (10 mg, 23 µmol, 1 equiv.), dimethylphenylsilane (10 µL, 68 µmol, 3 equiv.) and THF-$d_8$ (0.6 mL) in this order and was closed. The reaction mixture was heated up to 50 °C for 6 hours and crude the reaction mixture was analyzed *via* GC-MS and NMR spectroscopy.

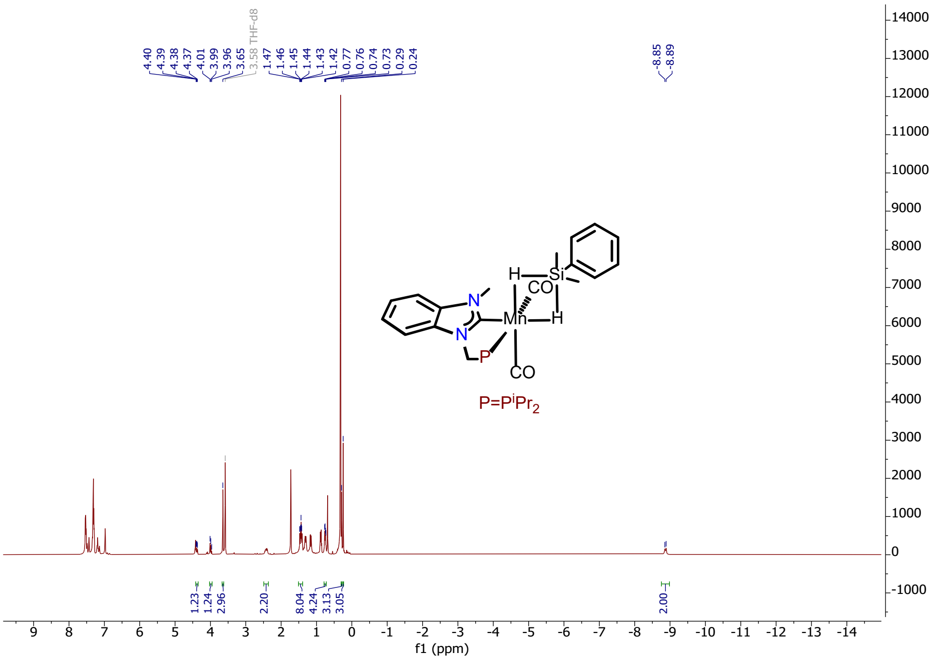


**Figure S1.** $^1$H-NMR spectrum of the reaction mixture of **1** with 3 equivs. of $SiHMe_2Ph$ in THF-$d_8$

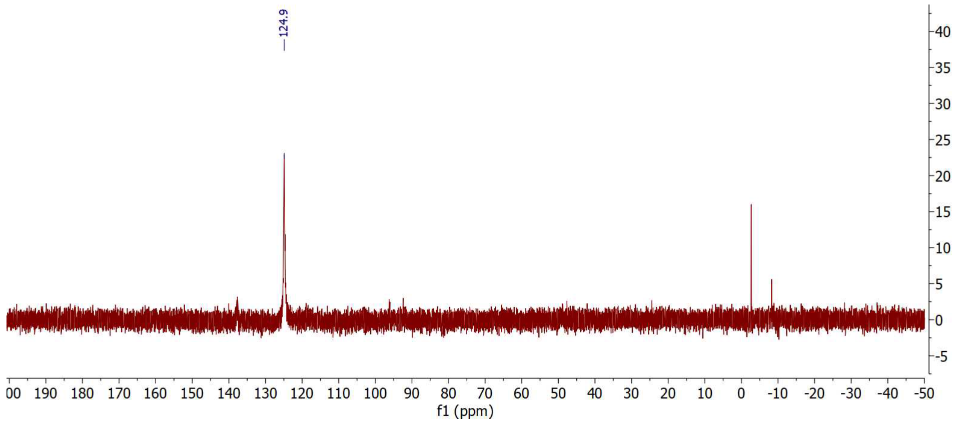


**Figure S2.** $^{31}P\{^1H\}$-NMR spectrum of the reaction mixture of **1** with 3 equivs. of $SiHMe_2Ph$ in THF-$d_8$

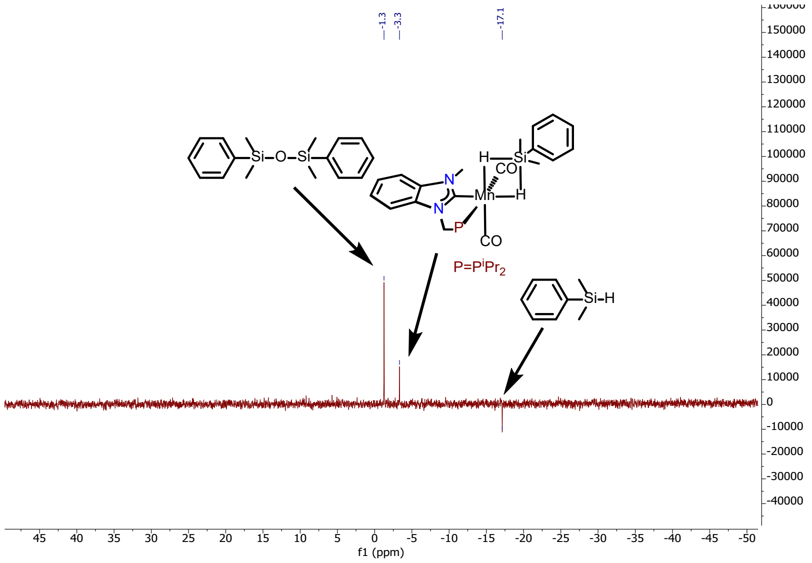


**Figure S3.** $^{29}Si\{^1H\}$-NMR spectrum of the reaction mixture of complex **1** with 3 equivs. $Me_2PhSiH$ in THF-$d_8$

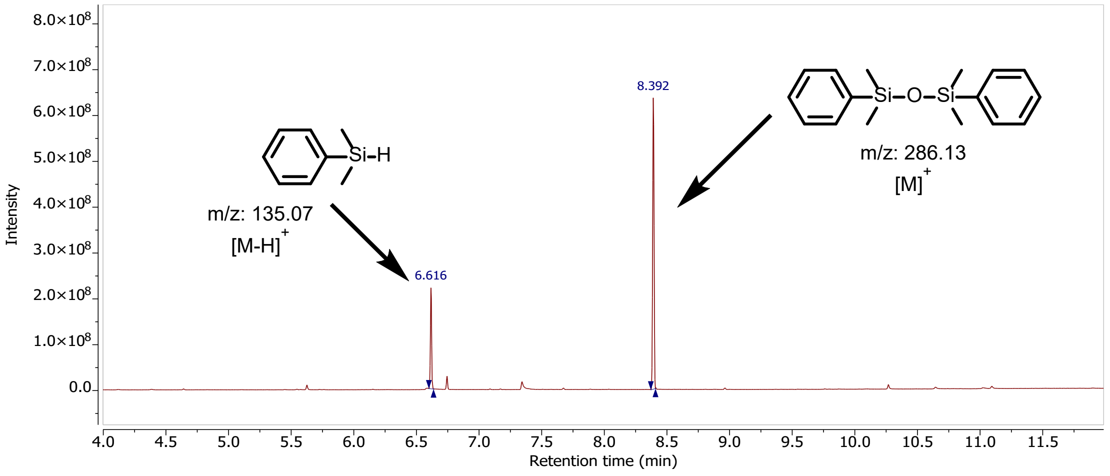


**Figure S4.** GC of the reaction mixture of complex **1** with 3 equivs. $SiHMe_2Ph$ with the corresponding m/z values.

## Computational Details

Calculations were performed using the GAUSSIAN 09 software package[9] and the PBE0 functional, without symmetry constraints. That functional uses a hybrid generalized gradient approximation (GGA), including 25 % mixture of Hartree-Fock[10] exchange with DFT[11] exchange-correlation, given by Perdew, Burke and Ernzerhof functional (PBE).[12] The optimized geometries were obtained with the Stuttgart Effective Core Potentials and associated basis set (SDD)[13] for Mn, and a standard 6-31G(d,p)[14] for the remaining elements (basis b1). Transition state optimizations were performed with the Synchronous Transit-Guided Quasi-Newton Method (STQN) developed by Schlegel *et al*,[15] following extensive searches of the Potential Energy Surface. Frequency calculations were performed to confirm the nature of the stationary points, yielding one imaginary frequency for the transition states and none for the minima. Each transition state was further confirmed by following its vibrational mode downhill on both sides and obtaining the minima presented on the energy profile. The electronic energies ($E_{b1}$) obtained at the PBE0/b1 level of theory were converted to free energy at 298.15 K and 1 atm ($G_{b1}$) by using zero-point energy and thermal energy corrections based on structural and vibration frequency data calculated at the same level.

Single point energy calculations were performed on the geometries obtained at the PBE0/b1 level using the same functional and a 6-311++G(d,p) basis set.[16] The free energy values presented ($G_{b2}$-D3) were corrected for dispersion by means of Grimme DFT-D3 method[17] with Becke and Johnson short distance damping,[18] being derived from the electronic energy values obtained at the PBE0-D3/6-311++G(d,p)//PBE0/b1 level ($E_{b2}$-D3) according to the following expression: $(G_{b2}\text{-D3}) = (E_{b2}\text{-D3}) + G_{b1} - E_{b1}$

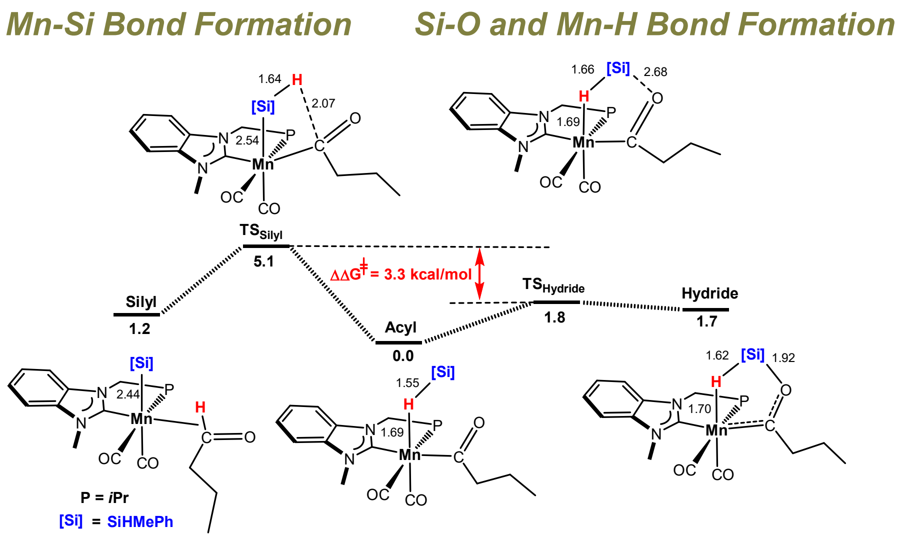


**Figure S5.** Formation of Mn-Silyl (Mn-Si Bond Formation) vs Mn-Hydride (Si-O Bond Formation) Intermediates

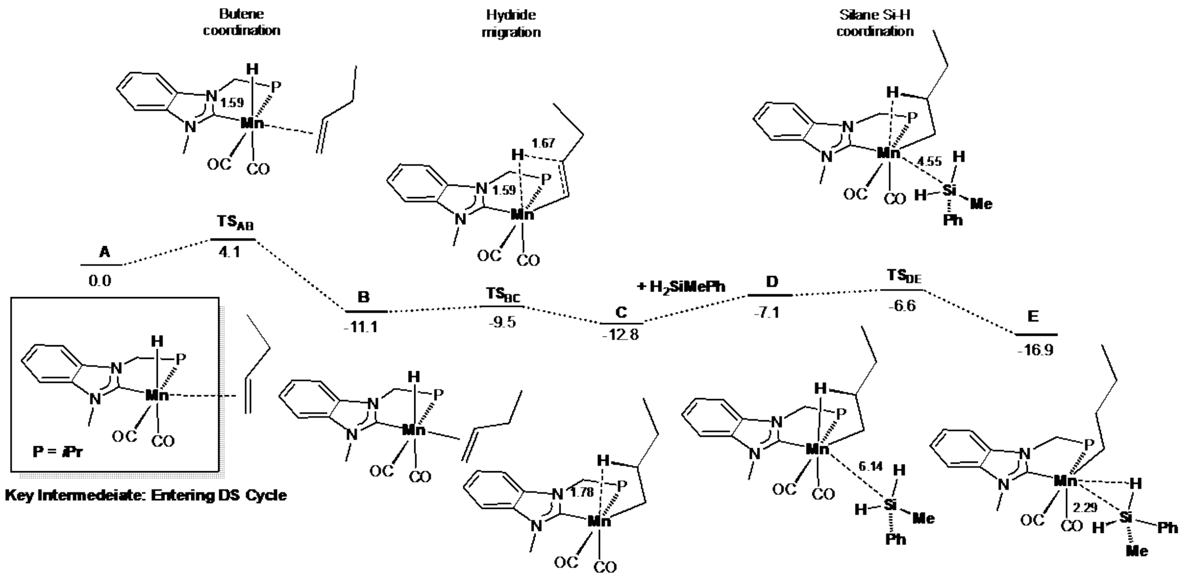


**Figure S6.** Free energy profile calculated for starting the DS cycle and forming the key intermediate **H** for entering the HD cycle. Free energies (kcal/mol) are referred to **A**: [Mn(PC-*i*Pr)(CO)$_2$(H)]···butene (**A**).

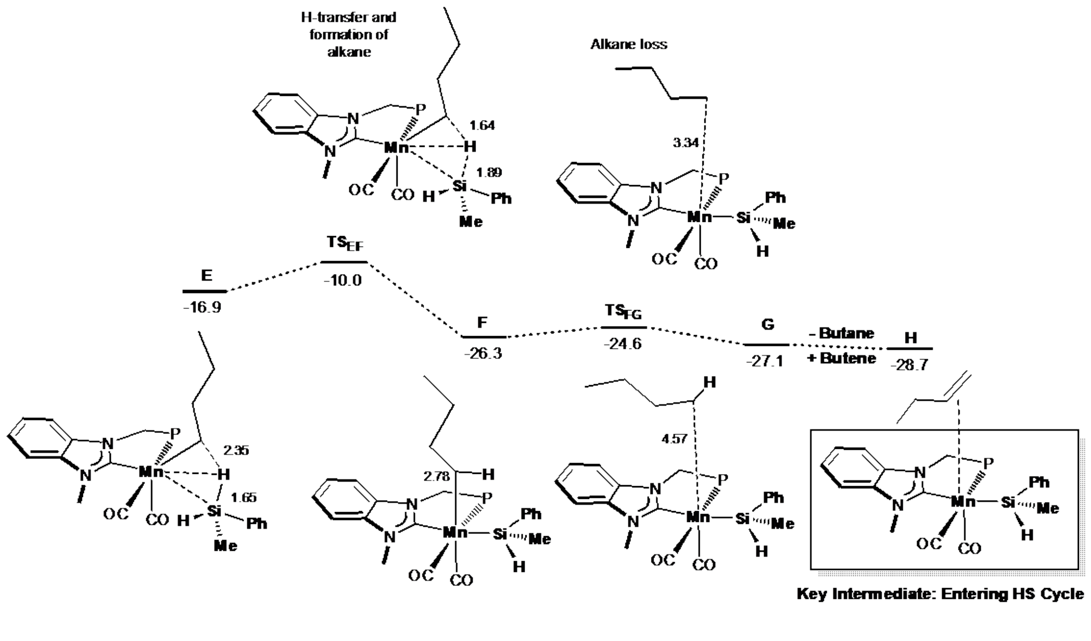


**Figure S7.** Free energy profile calculated for starting the DS cycle and forming the Key Intermediate **H** for Entering the HD Cycle (cont.). Free energies (kcal/mol) are referred to **A** (**A** = [Mn(PC-*i*Pr)(CO)$_2$(H)]···butene adduct).

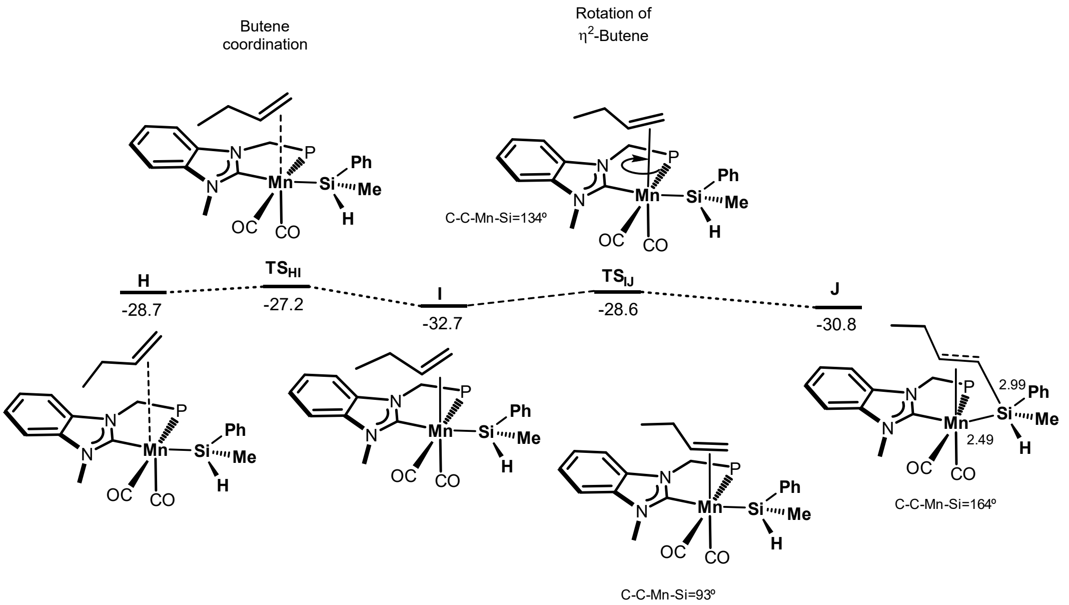


**Figure S8.** Free energy profile calculated for starting the HS cycle. Free energies (kcal/mol) are referred to **A** (**A** = [Mn(PC-*i*Pr)(CO)$_2$(H)]···butene adduct).

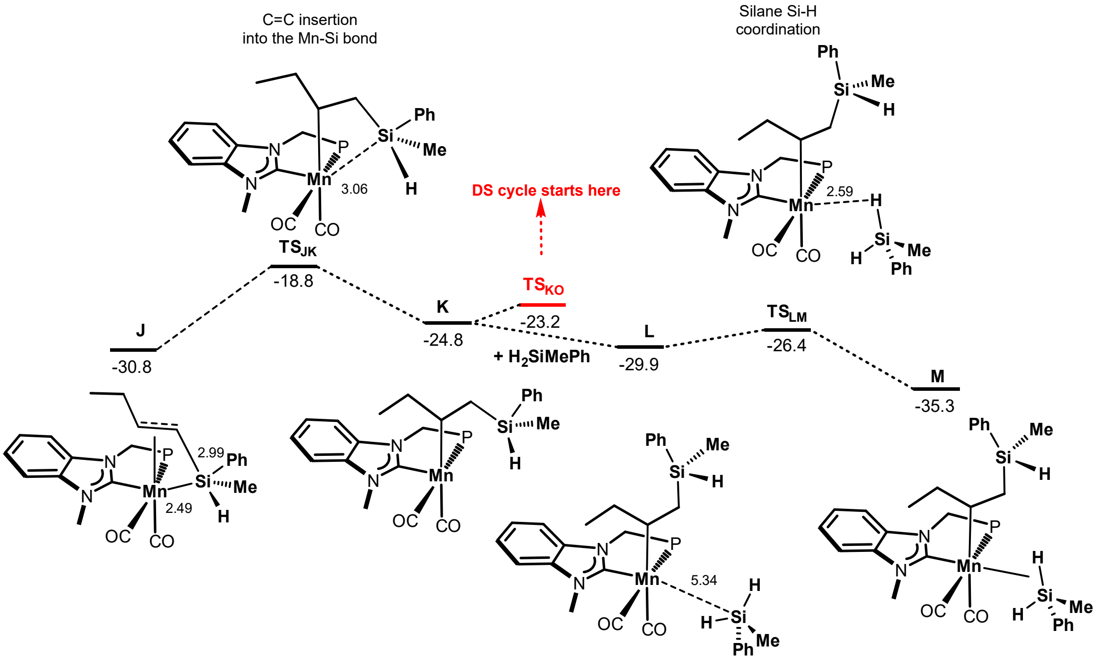


**Figure S9.** Free energy profile calculated for the HS cycle. Free energies (kcal/mol) are referred to **A** (**A** = [Mn(PC-*i*Pr)(CO)$_2$(H)]···butene adduct).

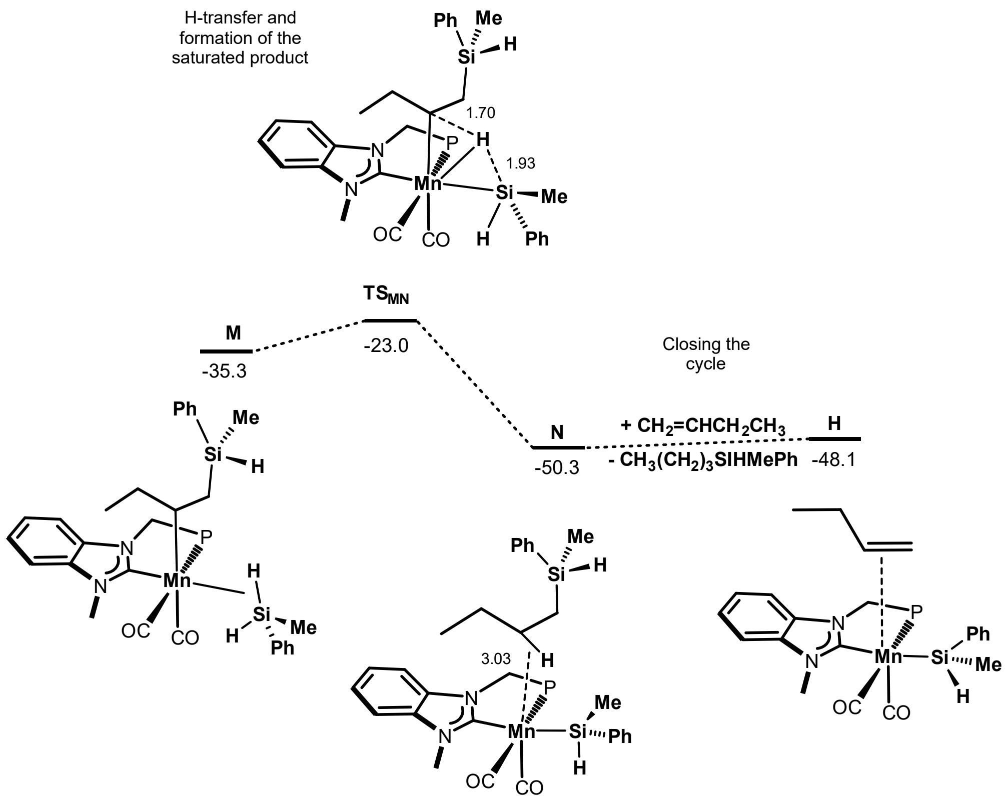


**Figure S10.** Free energy profile calculated for the HS cycle. Free energies (kcal/mol) are referred to **A** (**A** = [Mn(PC-*i*Pr)(CO)$_2$(H)]···butene adduct).

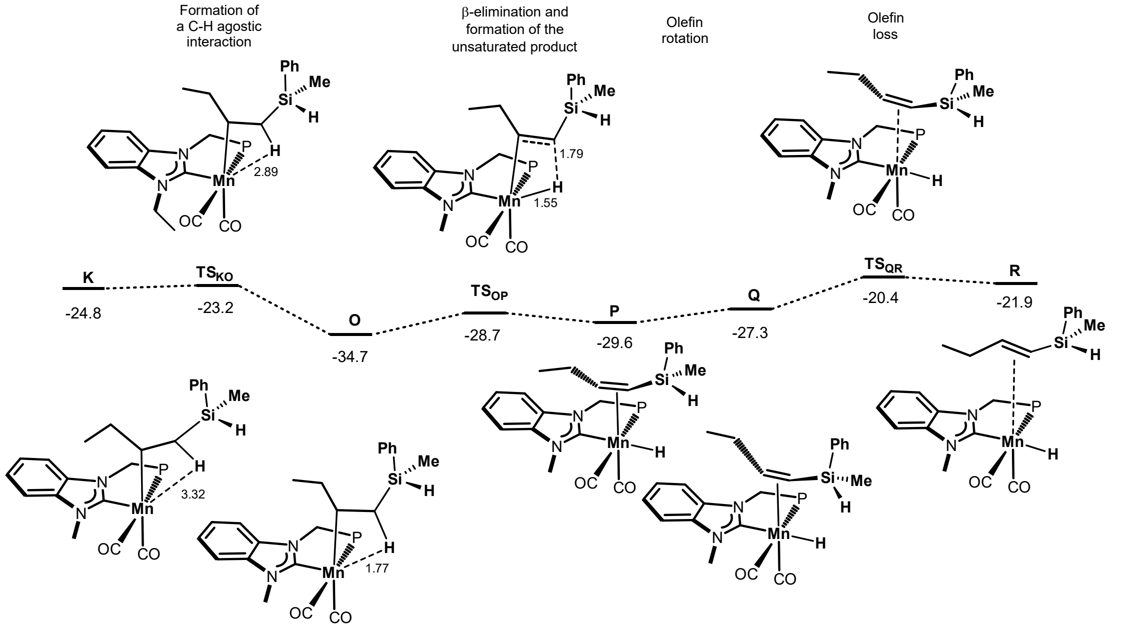


**Figure S11.** Free energy profile calculated for continuing the DS cycle. Free energies (kcal/mol) are referred to **A** (**A** = [Mn(PC-*i*Pr)(CO)$_2$(H)]···butene adduct).

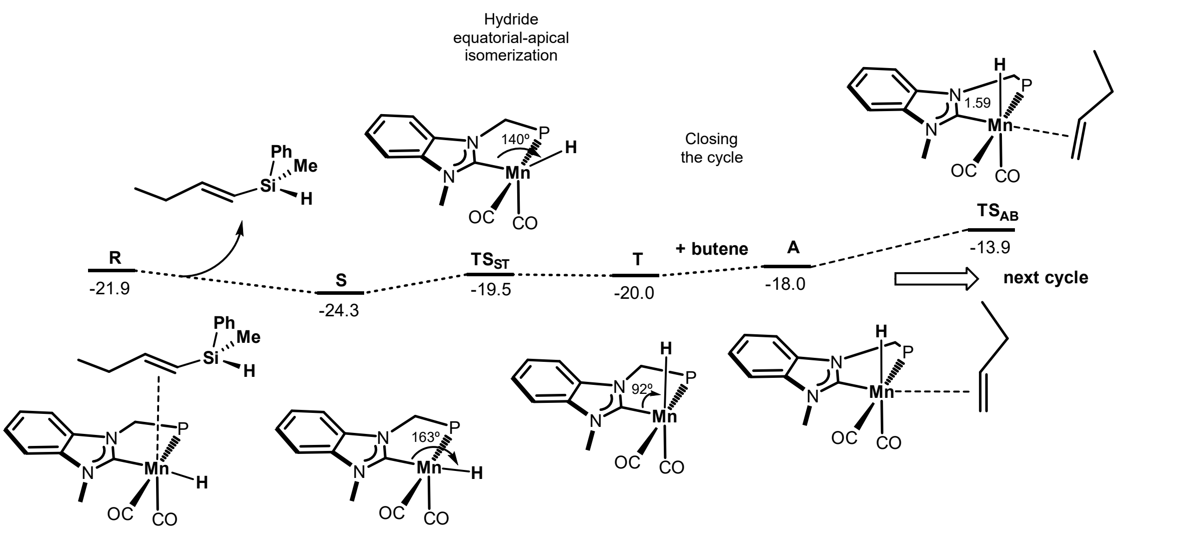


**Figure S12.** Free energy profile calculated for continuing the DS cycle. Free energies (kcal/mol) are referred to **A** (**A** = [Mn(PC-*i*Pr)$(CO)_2$(H)]···butene adduct).

## Characterization of Organic Products

### Methylphenyl(2-phenyleth-1-yl)silane (2a)

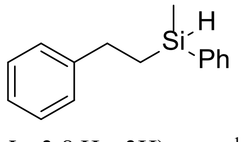


Styrene (57 µL, 0.5 mmol, 1 equiv.); methylphenylsilane (76 µL, 0.55 mmol, 1.1 equiv.); eluent: petroleum ether; yield: 108 mg (96%) of a colorless oil. $^{1}$H NMR (400 MHz, $CDCl_3$): δ = 7.62 – 7.51 (m, 2H), 7.45 – 7.32 (m, 3H), 7.31 – 7.24 (m, 2H), 7.23 – 7.13 (m, 3H), 4.41 (h, *J* = 3.6 Hz, 1H), 2.72 (t, *J* = 8.6 Hz, 2H), 1.32 – 1.13 (m, 2H), 0.36 (d, *J* = 3.8 Hz, 3H) ppm. $^{13}$C{$^{1}$H} NMR (101 MHz, $CDCl_3$): δ 144.7, 136.3, 134.5, 129.5, 128.5, 128.1, 128.0, 125.8, 30.6, 15.5, -5.6 ppm. $^{29}$Si{$^{1}$H} NMR (79 MHz, $CDCl_3$): δ = -13.5 ppm. This spectroscopic data corresponds to previously reported findings.[4]

### Phenyl(2-phenyleth-1-yl)silane (2b)

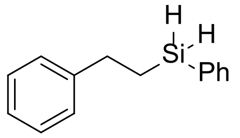


Styrene (57 µL, 0.5 mmol, 1 equiv.); phenylsilane (68 µL, 0.55 mmol, 1.1 equiv.); conversion: 83% (according to GC-MS). This spectroscopic data corresponds to previously reported findings.[5]

### Diphenyl(2-phenyleth-1-yl)silane (2c)

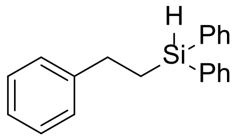


Styrene (57 µL, 0.5 mmol, 1 equiv.); diphenylsilane (102 µL, 0.55 mmol, 1.1 equiv.); purified *via* column chromatography, eluent: petroleum ether; yield: 115 mg (80%) of a colorless oil. $^{1}$H NMR (400 MHz, $CDCl_3$): δ = 7.62 – 7.54 (m, 4H), 7.44 – 7.35 (m, 6H), 7.31 – 7.23 (m, 2H), 7.23 – 7.13 (m, 3H), 4.90 (t, *J* = 3.6 Hz, 1H), 2.82 – 2.73 (m, 2H), 1.58 – 1.47 (m, 2H) ppm. $^{13}$C{$^{1}$H} NMR (101 MHz, $CDCl_3$) δ = 144.5, 135.3, 134.2, 129.8, 128.5, 128.2, 128.0, 125.9, 30.6, 14.4 ppm. $^{29}$Si{$^{1}$H} NMR (79 MHz, $CDCl_3$): δ = -14.1 ppm. This spectroscopic data corresponds to previously reported findings.[6]

**[2-(4-*tert*-Butylphenyl)eth-1-yl]methylphenylsilane (2d)**

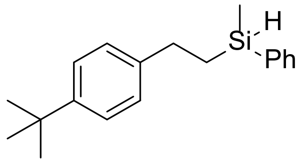


4-*tert*-butylstyrene (91 µL, 0.5 mmol, 1 equiv.); methylphenylsilane (76 µL, 0.55 mmol, 1.1 equiv.); eluent: petroleum ether; yield: 118 mg (84%) of a colorless oil $^{1}$H NMR (400 MHz, $CDCl_3$): δ = 7.58 – 7.53 (m, 2H), 7.42 – 7.34 (m, 3H), 7.33 – 7.27 (m, 2H), 7.17 – 7.10 (m, 2H), 4.41 (h, *J* = 3.6 Hz, 1H), 2.69 (t, *J* = 8.6 Hz, 2H), 1.31 (s, 9H), 1.29 – 1.15 (m, 2H), 0.36 (d, *J* = 3.7 Hz, 3H) ppm. $^{13}$C{$^{1}$H} NMR (101 MHz, $CDCl_3$): δ = 148.6, 141.6, 136.3, 134.5, 129.4, 128.0, 127.6, 125.3, 34.5, 31.6, 29.9, 15.4, -5.6 ppm. $^{29}$Si{$^{1}$H} NMR (79 MHz, $CDCl_3$): δ = -13.5 ppm. Anal. Calcd for $C_{19}H_{26}Si$: C, 80.78; H, 9.28. Found: C, 80.90; H, 9.18.

**[2-(4-Methoxyphenyl)eth-1-yl]methylphenylsilane (2e)**

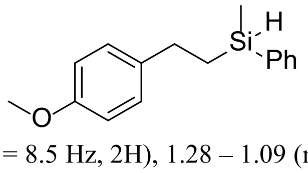


4-methoxystyrene (67 µL, 0.5 mmol, 1 equiv.); methylphenylsilane (76 µL, 0.55 mmol, 1.1 equiv.); eluent: petroleum ether; yield: 118 mg (92%) of a colorless oil $^{1}$H NMR (400 MHz, $CDCl_3$): δ = 7.61 – 7.50 (m, 2H), 7.44 – 7.32 (m, 3H), 7.16 – 7.06 (m, 2H), 6.91 – 6.77 (m, 2H), 4.39 (h, *J* = 3.6 Hz, 1H), 3.79 (s, 3H), 2.66 (t, *J* = 8.5 Hz, 2H), 1.28 – 1.09 (m, 2H), 0.35 (d, *J* = 3.8 Hz, 3H) ppm. $^{13}$C{$^{1}$H} NMR (101 MHz, $CDCl_3$): δ = 157.8, 136.8, 136.3, 134.5, 129.4, 128.8, 128.0, 113.9, 55.4, 29.6, 15.8, -5.6 ppm. $^{29}$Si{$^{1}$H} NMR (79 MHz, $CDCl_3$): δ = -13.7 ppm. Anal. Calcd for $C_{16}H_{20}Si$: C, 74.95; H, 7.68. Found: C, 75.02; H, 7.65.

**[2-(4-Chlorophenyl)eth-1-yl]methylphenylsilane (2f)**

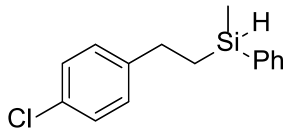


4-chlorostyrene (60 µL, 0.5 mmol, 1 equiv.); methylphenylsilane (76 µL, 0.55 mmol, 1.1 equiv.); eluent: petroleum ether; yield: 122 mg (94%) of a colorless oil. $^{1}$H NMR (400 MHz, $CDCl_3$): δ = 7.59 – 7.48 (m, 2H), 7.46 – 7.31 (m, 3H), 7.24 – 7.20 (m, 2H), 7.15 – 7.05 (m, 2H), 4.39 (h, *J* = 3.6 Hz, 1H), 2.67 (t, *J* = 8.5 Hz, 2H), 1.29 – 1.08 (m, 2H), 0.35 (d, *J* = 3.8 Hz, 3H) ppm. $^{13}$C{$^{1}$H} NMR (101 MHz, $CDCl_3$): δ = 143.0, 136.0, 134.5, 131.4, 129.6, 129.3, 128.5, 128.1, 30.0, 15.5, -5.6 ppm. $^{29}$Si{$^{1}$H} NMR (79 MHz, $CDCl_3$): δ = -13.6 ppm. This spectroscopic data corresponds to previously reported findings.[4]

**{2-[4-(Trifluoromethyl)phenyl]eth-1-yl}methylphenylsilane (2g)**

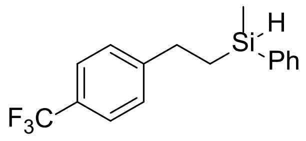


4-(trifluoromethyl)styrene (74 µL, 0.5 mmol, 1 equiv.); methylphenylsilane (76 µL, 0.55 mmol, 1.1 equiv.); eluent: petroleum ether; yield: 134 mg (91%) of a colorless oil. $^{1}$H NMR (400 MHz, $CDCl_3$): δ = 7.57 – 7.53 (m, 2H), 7.53 – 7.49 (m, 2H), 7.42 – 7.35 (m, 3H), 7.30 – 7.26 (m, 2H), 4.41 (h, *J* = 3.6 Hz, 1H), 2.76 (t, *J* = 8.5 Hz, 2H), 1.33 – 1.12 (m, 2H), 0.38 (d, *J* = 3.8 Hz, 3H) ppm. $^{13}$C{$^{1}$H} NMR (101 MHz, $CDCl_3$): δ = 148.7, 135.8, 134.4, 133.5, 129.6, 128.2 (d, *J* = 14.7 Hz), 125.9, 125.4 (q, *J* = 3.9 Hz), 123.2, 30.5, 15.4, -5.6 ppm. $^{29}$Si{$^{1}$H} NMR (79 MHz, $CDCl_3$): δ = -13.4 ppm. Anal. Calcd for $C_{16}H_{17}F_3Si$: C, 65.28; H, 5.82. Found: C, 65.33; H, 5.91.

**[2-(3-Bromophenyl)eth-1-yl]methylphenylsilane (2h)**

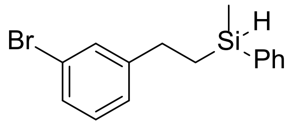


3-bromostyrene (65 µL, 0.5 mmol, 1 equiv.); methylphenylsilane (76 µL, 0.55 mmol, 1.1 equiv.); eluent: petroleum ether and dichloromethane (3:1); yield: 143 mg (93%) of a colorless oil. $^{1}$H NMR (400 MHz, $CDCl_3$): δ = 7.60 – 7.51 (m, 2H), 7.44 – 7.35 (m, 3H), 7.35 – 7.25 (m, 2H), 7.17 – 7.06 (m, 2H), 4.40 (h, *J* = 3.6 Hz, 1H), 2.67 (t, *J* = 8.5 Hz, 2H), 1.26 – 1.12 (m, 2H), 0.36 (d, *J* = 3.8 Hz, 3H) ppm. $^{13}$C{$^{1}$H} NMR (101 MHz, $CDCl_3$): δ = 147.0, 135.9, 134.5, 131.1, 130.0, 129.6, 128.9, 128.1, 126.7, 122.5, 30.3, 15.4, -5.6 ppm. $^{29}$Si{$^{1}$H} NMR (79 MHz, $CDCl_3$): δ = -13.5 ppm. This spectroscopic data corresponds to previously reported findings.[1]

**[2-(2-Chlorophenyl)eth-1-yl]methylphenylsilane (2i)**

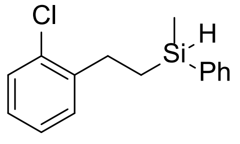


2-chlorostyrene (64 µL, 0.5 mmol, 1 equiv.); methylphenylsilane (76 µL, 0.55 mmol, 1.1 equiv.); eluent: petroleum ether and dichloromethane (3:1); yield: 123 mg (95%) of a colorless oil. $^{1}$H NMR (400 MHz, $CDCl_3$): δ = 7.62 – 7.54 (m, 2H), 7.43 – 7.35 (m, 3H), 7.35 – 7.28 (m, 1H), 7.24 – 7.06 (m, 3H), 4.43 (h, *J* = 3.6 Hz, 1H), 2.80 (t, *J* = 8.6 Hz, 2H), 1.32 – 1.11 (m, 2H), 0.39 (d, *J* = 3.8 Hz, 3H) ppm. $^{13}$C{$^{1}$H} NMR (101 MHz, $CDCl_3$): δ = 142.2, 136.1, 134.5, 133.7, 129.8, 129.6, 129.5, 128.1, 127.3, 126.9, 28.6, 14.0, -5.6 ppm. $^{29}$Si{$^{1}$H} NMR (79 MHz, $CDCl_3$): δ = -13.5 ppm. Anal. Calcd for $C_{15}H_{17}ClSi$: C, 69.07; H, 6.57. Found: C, 69.33; H, 6.49.

**[2-(2,3,4,5,6-Pentafluorophenyl)eth-1-yl]methylphenylsilane (2j)**

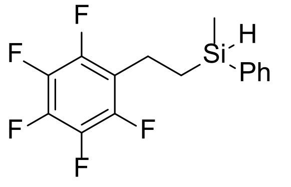


2,3,4,5,6-pentafluorostyrene (69 µL, 0.5 mmol, 1 equiv.); methylphenylsilane (76 µL, 0.55 mmol, 1.1 equiv.); eluent: petroleum ether; yield: 156 mg (99%) of a colorless oil. $^{1}$H NMR (400 MHz, $CDCl_3$) δ 7.58 – 7.46 (m, 2H), 7.44 – 7.31 (m, 3H), 4.39 (h, *J* = 3.6 Hz, 1H), 2.76 (tt, *J* = 8.4, 1.8 Hz, 2H), 1.28 – 1.09 (m, 2H), 0.40 (d, *J* = 3.8 Hz, 3H). $^{13}$C{$^{1}$H} NMR (101 MHz, $CDCl_3$): δ = 135.2, 134.3, 129.7, 128.1, 17.5, 13.9, -5.7 ppm. (**C**-F not detected). $^{29}$Si{$^{1}$H} NMR (79 MHz, $CDCl_3$): δ = -13.8 ppm. Anal. Calcd for $C_{15}H_{13}F_5Si$: C, 56.95; H, 4.14. Found: C, 57.13; H, 4.01.

**Methyl-[2-(naphthyl)eth-1-yl]phenylsilane (2k)**

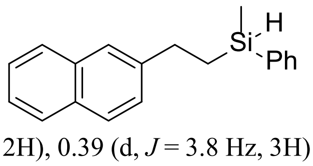


2-vinylnapthalene (77 mg, 0.5 mmol, 1 equiv.); methylphenylsilane (76 µL, 0.55 mmol, 1.1 equiv.); eluent: petroleum ether; yield: 122 mg (88%) of a colorless oil $^{1}$H NMR (400 MHz, $CDCl_3$): δ = 7.84 – 7.73 (m, 3H), 7.66 – 7.52 (m, 3H), 7.48 – 7.32 (m, 6H), 4.44 (h, *J* = 3.6 Hz, 1H), 2.88 (t, *J* = 8.4 Hz, 2H), 1.40 – 1.21 (m, 2H), 0.39 (d, *J* = 3.8 Hz, 3H) ppm. $^{13}$C{$^{1}$H} NMR (101 MHz, $CDCl_3$): δ 142.1, 136.2, 134.5, 133.8, 132.1, 129.5, 128.1, 128.0, 127.7, 127.6, 127.1, 126.0, 125.7, 125.2, 30.8, 15.5, -5.5 ppm. $^{29}$Si{$^{1}$H} NMR (79 MHz, $CDCl_3$): δ = -13.4 ppm. Anal. Calcd for $C_{19}H_{20}Si$: C, 82.55; H, 7.29. Found: C, 82.43; H, 7.18.

**4-[2-(Methylphenylsilyl)ethyl]phenyl acetate (2l)**

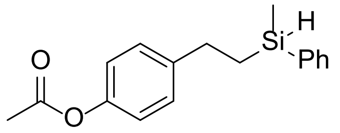


4-vinylphenyl acetate (76 µL, 0.5 mmol, 1 equiv.); methylphenylsilane (76 µL, 0.55 mmol, 1.1 equiv.); conversion: 73% (according to GC-MS). This compound could not be isolated in pure form.

**Methyl-1-octylphenylsilane (2m)**

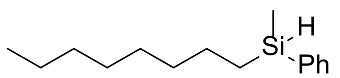


1-Octene (79 µL, 0.5 mmol, 1 equiv.); methylphenylsilane (76 µL, 0.55 mmol, 1.1 equiv.); eluent: petroleum ether; yield: 102 mg (87%) of a colorless oil. $^{1}$H NMR (400 MHz, $CDCl_3$): δ = 7.61 – 7.48 (m, 2H), 7.42 – 7.30 (m, 3H), 4.34 (h, *J* = 3.7 Hz, 1H), 1.45 – 1.19 (m, 12H), 0.95 – 0.74 (m, 5H), 0.33 (d, *J* = 3.8 Hz, 3H) ppm. $^{13}$C{$^{1}$H} NMR (101 MHz, $CDCl_3$): δ = 137.0, 134.4, 129.3, 128.0, 33.3, 32.1, 29.4, 29.4, 24.5, 22.8, 14.3, 13.5, -5.5 ppm. $^{29}$Si{$^{1}$H} NMR (79 MHz, $CDCl_3$): δ = -13.6 ppm. This spectroscopic data corresponds to previously reported findings.[7]

**(6-Chlorohexyl)methylphenylsilane (2n)**

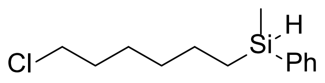


6-chlorohexene (66 µL, 0.5 mmol, 1 equiv.); methylphenylsilane (76 µL, 0.55 mmol, 1.1 equiv.); eluent: petroleum ether; yield: 109 mg (91%) of a colorless oil. $^{1}$H NMR (400 MHz, $CDCl_3$): δ = 7.59 – 7.49 (m, 2H), 7.44 – 7.30 (m, 3H), 4.35 (h, *J* = 3.6 Hz, 1H), 3.51 (t, *J* = 6.7 Hz, 2H), 1.85 – 1.67 (m, 2H), 1.50 – 1.29 (m, 6H), 0.93 – 0.75 (m, 2H), 0.34 (d, *J* = 3.8 Hz, 3H) ppm. $^{13}$C{$^{1}$H} NMR (101 MHz, $CDCl_3$): δ = 136.7, 134.4, 129.4, 128.0, 45.3, 32.7, 32.5, 26.7, 24.3, 13.4, -5.5 ppm. $^{29}$Si{$^{1}$H} NMR (79 MHz, $CDCl_3$): δ = -13.6 ppm. Anal. Calcd for $C_{13}H_{21}ClSi$: C, 64.83; H, 8.79. Found: C, 64.74; H, 8.70.

**Methylphenyl(4-phenylbut-1-yl)silane (2o)**

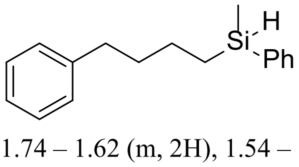

4-phenyl-1-butene (75 µL, 0.5 mmol, 1 equiv.); methylphenylsilane (76 µL, 0.55 mmol, 1.1 equiv.); eluent: petroleum ether; yield: 119 mg (94%) of a colorless oil $^{1}$H NMR (400 MHz, $CDCl_3$): δ = 7.59 – 7.51 (m, 2H), 7.40 – 7.33 (m, 3H), 7.29 – 7.24 (m, 2H), 7.22 – 7.09 (m, 3H), 4.36 (hept, *J* = 3.6 Hz, 1H), 2.66 – 2.56 (m, 2H), 1.74 – 1.62 (m, 2H), 1.54 – 1.40 (m, 2H), 0.98 – 0.79 (m, 2H), 0.34 (d, *J* = 3.8 Hz, 3H) ppm. $^{13}$C{$^{1}$H} NMR (101 MHz, $CDCl_3$): δ = 142.9, 136.8, 134.4, 129.3, 128.5, 128.4, 128.0, 125.7, 35.7, 35.1, 24.2, 13.4, -5.5 ppm. $^{29}$Si{$^{1}$H} NMR (79 MHz, $CDCl_3$): δ = -13.6 ppm. Anal. Calcd for $C_{17}H_{22}Si$: C, 80.25; H, 8.72. Found: C, 80.35; H, 8.79.

**Methylphenyl(3-phenylprop-1-yl)silane (2p)**

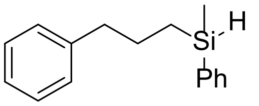

allylbenzene (66 µL, 0.5 mmol, 1 equiv.); methylphenylsilane (76 µL, 0.55 mmol, 1.1 equiv.); conversion: 55 % (determined by GC-MS). This compound could not be isolated in pure form.

**Methyl-3-[(trimethylsilyl)propyl]phenylsilane (2q)**

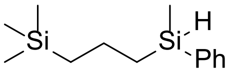

allyltrimethylsilane (79 µL, 0.5 mmol, 1 equiv.); methylphenylsilane (76 µL, 0.55 mmol, 1.1 equiv.); conversion: 50 % (determined by GC-MS) This compound could not be isolated in pure form.

**Dimethyl(2-methyl-2-phenyleth-1-yl)phenylsilane (2r)**

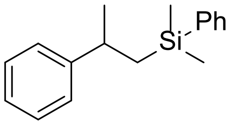

α-methylstyrene (65 µL, 0.5 mmol, 1 equiv.); dimethyllphenylsilane (84 µL, 0.55 mmol, 1.1 equiv.); conversion: 98 % (determined by GC-MS). This compound could not be isolated in pure form.

**Dimethylphenyl-[(1*E*)-2-phenylethen-1-yl]silane (3a)**

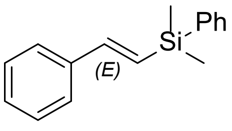

styrene (103 µL, 0.9 mmol, 1.8 equiv.); dimethylphenylsilane (77 µL, 0.5 mmol, 1.0 equiv.); catalyst (2.2 mg, 1 mol%); eluent: petroleum ether; yield: 109 mg (92%) of a colorless oil. $^{1}$H NMR (400 MHz, $CDCl_3$): δ = 7.52 – 7.42 (m, 2H), 7.37 – 7.33 (m, 2H), 7.29 – 7.25 (m, 3H), 7.25 – 7.19 (m, 2H), 7.15 (ddt, *J* = 7.3, 6.2, 1.4 Hz, 1H), 6.84 (d, *J* = 19.1 Hz, 1H), 6.49 (d, *J* = 19.1 Hz, 1H), 0.33 (s, 6H) ppm. $^{13}$C{1H} NMR (101 MHz, $CDCl_3$): δ = 145.4, 138.7, 138.3, 134.1, 129.2, 128.7, 128.3, 128.0, 127.3, 126.6, -2.4 ppm.$^{29}$Si{$^{1}$H} NMR (79 MHz, $CDCl_3$): δ = -10.4 ppm. The spectroscopic data correspond to previously reported findings.[8]

**[(1*E*)-2-(4-*tert*-butylphenyl)ethen-1-yl]dimethylphenylsilan (3b)**

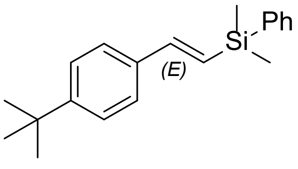

4-*tert*-butylstyrene (164 µL, 0.9 mmol, 1.8 equiv.); dimethylphenylsilane (77 µL, 0.5 mmol, 1.0 equiv.); catalyst (2.2 mg, 1 mol%); eluent: petroleum ether; yield: 134 mg (91%) of a colorless oil. $^{1}$H NMR (400 MHz, $CDCl_3$): δ = 7.61 – 7.54 (m, 2H), 7.43 – 7.33 (m, 7H), 6.94 (d, *J* = 19.1 Hz, 1H), 6.54 (d, *J* = 19.1 Hz, 1H), 1.32 (s, 9H), 0.43 (s, 6H) ppm. $^{13}$C{$^{1}$H} NMR (101 MHz, $CDCl_3$): δ = 151.5, 145.2, 138.9, 135.6, 134.1, 129.1, 127.9, 126.4, 126.2, 125.6, 34.8, 31.4, -2.3 ppm. $^{29}$Si{$^{1}$H} NMR (79 MHz, $CDCl_3$): δ = -10.4 ppm. The spectroscopic data correspond to previously reported findings.[8]

**[(1*E*)-2-(4-Methoxyphenyl)ethen-1-yl]dimethylphenylsilane (3c)**

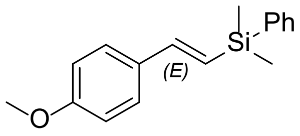

4-vinylanisole (121 µL, 0.9 mmol, 1.8 equiv.); dimethylphenylsilane (77 µL, 0.5 mmol, 1.0 equiv.); catalyst (2.2 mg, 1 mol%); eluent: petroleum ether; yield: 128 mg (96%) of a colorless oil. $^{1}$H NMR (400 MHz, $CDCl_3$): δ = 7.62 – 7.54 (m, 2H), 7.42 – 7.33 (m, 5H), 6.90 – 6.82 (m, 3H), 6.41 (d, *J* = 19.1 Hz, 1H), 3.82 (s, 3H), 0.42 (s, 6H) ppm. $^{13}$C{$^{1}$H} NMR (101 MHz, $CDCl_3$): δ = 159.9, 144.9, 139.0, 134.1, 131.3, 129.1, 128.8, 127.9, 124.3, 114.0, 55.5, -2.3 ppm. $^{29}$Si{$^{1}$H} NMR (79 MHz, $CDCl_3$): δ = -10.5 ppm. This spectroscopic data corresponds to previously reported findings.[8]

**[(1*E*)-2-(4-Chlorophenyl)ethen-1-yl]dimethylphenylsilane (3d)**

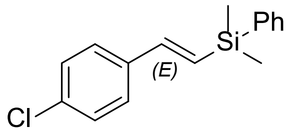

4-chlorostyrene (108 µL, 0.9 mmol, 1.8 equiv.); dimethylphenylsilane (77 µL, 0.5 mmol, 1.0 equiv.); catalyst (2.2 mg, 1 mol%); eluent: petroleum ether; yield: 115 mg (85%) of a colorless oil. $^{1}$H NMR (400 MHz, $CDCl_3$): δ = 7.61 – 7.51 (m, 2H), 7.39 – 7.35 (m, 5H), 7.33 – 7.27 (m, 2H), 6.88 (d, *J* = 19.1 Hz, 1H), 6.56 (d, *J* = 19.1 Hz, 1H), 0.44 (s, 6H) ppm. $^{13}$C{$^{1}$H} NMR (101 MHz, $CDCl_3$): δ = 144.0, 138.4, 136.8, 134.0, 133.9, 129.3, 128.8, 128.3, 128.0, 127.8, -2.5 ppm. $^{29}$Si{$^{1}$H} NMR (79 MHz, $CDCl_3$): δ = -10.3 ppm. This spectroscopic data corresponds to previously reported findings.[8]

**[(1*E*)-2-(3-Bromophenyl)ethen-1-yl]dimethylphenylsilane (3e)**

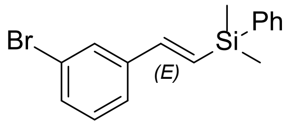

3-bromostyrene (117 µL, 0.9 mmol, 1.8 equiv.); dimethylphenylsilane (77 µL, 0.5 mmol, 1.0 equiv.); catalyst (2.2 mg, 1 mol%); eluent: petroleum ether; yield: 136 mg (86%) of a colorless oil. $^{1}$H NMR (400 MHz, $CDCl_3$): δ = 7.62 – 7.52 (m, 3H), 7.39 – 7.33 (m, 5H), 7.19 (t, *J* = 7.8 Hz, 1H), 6.85 (d, *J* = 19.1 Hz, 1H), 6.61 (d, *J* = 19.0 Hz, 1H), 0.44 (s, 6H) ppm. $^{13}$C{$^{1}$H} NMR (101 MHz, $CDCl_3$): δ = 143.7, 140.4, 138.2, 134.0, 131.1, 130.2, 129.5, 129.5, 129.3, 128.0, 125.3, 123.0, -2.5 ppm. $^{29}$Si{$^{1}$H} NMR (79 MHz, $CDCl_3$): δ = -10.2 ppm. Anal. Calcd for $C_{16}H_{17}BrSi$: C, 60.57; H, 5.40. Found: C, 60.35; H, 5.51.

**Dimethyl[(1*E*)-2-(2,4,6-trimethylphenyl)ethen-1-yl]phenylsilane (3f)**

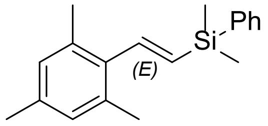

2,4,6-trimethylstyrene (132 µL, 0.9 mmol, 1.8 equiv.); dimethylphenylsilane (77 µL, 0.5 mmol, 1.0 equiv.); catalyst (2.2 mg, 1 mol%); eluent: petroleum ether; yield: 71 mg (51%) of a colorless oil. $^{1}$H NMR (400 MHz, $CDCl_3$): δ = 7.66 – 7.59 (m, 2H), 7.45 – 7.34 (m, 3H), 6.97 (d, *J* = 19.6 Hz, 1H), 6.88 (s, 2H), 6.07 (d, *J* = 19.6 Hz, 1H), 2.29 (s, 9H), 0.46 (s, 6H) ppm. $^{13}$C{$^{1}$H} NMR (101 MHz, $CDCl_3$): δ = 144.5, 139.0, 136.5, 136.3, 135.4, 134.0, 133.3, 129.1, 128.7, 127.9, 21.1, 20.9, -2.3 ppm. $^{29}$Si{$^{1}$H} NMR (79 MHz, $CDCl_3$): δ = -10.7 ppm. Anal. Calcd for $C_{19}H_{24}Si$: C, 81.36; H, 8.63. Found: C, 81.39; H, 8.61.

**N-[(1*E*)-2-(Dimethylphenylsilyl)ethenyl]-9*H*-carbazole (3g)**

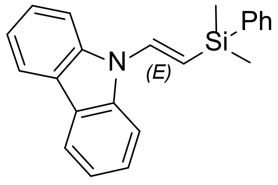

N-Vinylcarbazole (146 mg, 0.9 mmol, 1.8 equiv.); dimethylphenylsilane (77 µL, 0.5 mmol, 1.0 equiv.); catalyst (2.2 mg, 1 mol%); conversion: >99% (according to GC-MS). This compound could not ne isolated in pure form.

**(Cyclohexylidenemethyl)dimethylphenylsilane (3h)**

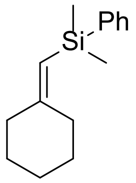

Methylenecyclohexane (135 µL 0.9 mmol, 1.8 equiv.); dimethylphenylsilane (77 µL, 0.50 mmol, 1.0 equiv.); conversion: 50 % (determined by GC-MS). This compound could not be isolated in pure form.

**Dimethyl(2-methyl-2-phenyl-1-ethen-1-yl)phenylsilane/Dimethylphenyl[1-(phenylmethyl)ethenyl]silane (3i)**

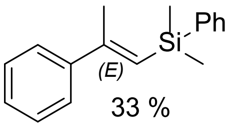

33 %

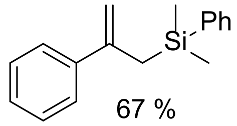

67 %

α-Methylstyrene (117 µL, 0.9 mmol, 1.8 equiv.); dimethylphenylsilane (77 µL, 0.50 mmol, 1.1 equiv.); conversion: 92 % (determined by GC-MS); isomer ratio determined *via* $^{1}$H-NMR spectroscopy. This compound could nobne isolated in pure form.

**Dimethyl-1-octenylphenylsilane/Dimethyl-2-octenylphenylsilane (3j)**

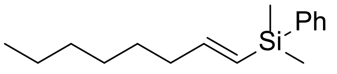

17 %

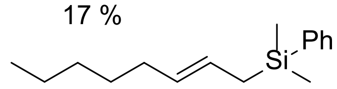

83 % (*E*/*Z*: 65/35)

1-Octene (142 µL, 0.9 mmol, 1.8 equiv.); dimethylphenylsilane (77 µL, 0.50 mmol, 1.1 equiv.); conversion: >99 % (determined by GC-MS); isomer ratio determined *via* $^{1}$H-NMR spectroscopy, *E*/*Z* ratio determined *via* GC-MS. This compound could not be isolated in pure form.

## References


(1) Zobernig, D. P.; Luxner, M.; Stöger, B.; Veiros, L. F.; Kirchner, K. Hydrogenation of Terminal Alkenes Catalyzed by Air-Stable Mn(I) Complexes Bearing an N-Heterocyclic Carbene-Based PCP Pincer Ligand. *Chem. Eur. J.* **2024**, *30*, e202302455.

(2) Zobernig, D. P.; Stöger, B.; Veiros, L. F.; Kirchner, K. Hydroboration of Terminal Alkynes Catalyzed by a Mn(I) Alkyl PCP Pincer Complex Following Two Diverging Pathways. *ACS Catal.* **2024**, 14, 12385–12391.

(3) Zobernig, D. P.; Stöger, B.; Veiros, L. F.; Kirchner, K. Hydrogenation of Alkenes Catalyzed by Mn(I) Alkyl Complexes Bearing NHC Phosphine Ligands. *ChemCatChem* **2024**, *n/a*, e202401172.

(4) X. Fan, M. Zhang, Y. Gao, Q. Zhou, Y. Zhang, J. Yu, W. Xu, J. Yan, H. Liu, Z. Lei, Y. C. Ter, S. Chanmungkalakul, Y. Lum, X. Liu, G. Cui, J. Wu, *Nat. Chem.* **2023**, *15*, 666–676.

(5) Du, X.; Zhang, Y.; Peng, D.; Huang, Z. Base–Metal-Catalyzed Regiodivergent Alkene Hydrosilylations. *Angew. Chem., Int. Ed.* **2016**, *55*, 6671–6675.

(6) Baruah, J. B.; Osakada, K.; Yamamoto, T. $RhCl(PPh_3)_3$ catalyzed hydrosilylation of styrene and phenylacetylene with phenylsilanes *J. Mol. Catal. A: Chemical* **1995**, *101*, 17-24.

(7) K. Kamata, A. Suzuki, Y. Nakai, H. Nakazawa, *Organometallics* **2012**, *31*, 3825–3828.

(8) Weber, S.; Glavic, M.; Stöger, B.; Pittenauer, E.; Podewitz, M.; Veiros, L. F.; Kirchner, K. Manganese-Catalyzed Dehydrogenative Silylation of Alkenes Following Two Parallel Inner-Sphere Pathways. *J. Am. Chem. Soc.* **2021**, *143*, 17825–17832.

(9) GAUSSIAN 09, Revision **A.01**, Frisch, M. J.; Trucks, G. W.; Schlegel, H. B.; Scuseria, G. E.; Robb, M. A.; Cheeseman, J. R.; Scalmani, G.; Barone, V.; Mennucci, B.; Petersson, G. A.; Nakatsuji, H.; Caricato, M.; Li, X.; Hratchian, H. P.; Izmaylov, A. F.; Bloino, J.; Zheng, G.; Sonnenberg, J. L.; Hada, M.; Ehara, M.; Toyota, K.; Fukuda, R.; Hasegawa, J.; Ishida, M.; Nakajima, T.; Honda, Y.; Kitao, O.; Nakai, H.; Vreven, T.; Montgomery, Jr., J. A.; Peralta, J. E.; Ogliaro, F.; Bearpark, M.; Heyd, J. J.; Brothers, E.; Kudin, K. N.; Staroverov, V. N.; Kobayashi, R.; Normand, J.; Raghavachari, K.; Rendell, A.; Burant, J. C.; Iyengar, S. S.; Tomasi, J.; Cossi, M.; Rega, N.; Millam, J. M.; Klene, M.; Knox, J. E.; Cross, J. B.; Bakken, V.; Adamo, C.; Jaramillo, J.; Gomperts, R.; Stratmann, R. E.; Yazyev, O.; Austin, A. J.; Cammi, R.; Pomelli, C.; Ochterski, J. W.; Martin, R. L.;

Morokuma, K.; Zakrzewski, V. G.; Voth, G. A.; Salvador, P.; Dannenberg, J. J.; Dapprich, S.; Daniels, A. D.; Farkas, Ö.; Foresman, J. B.; Ortiz, J. V.; Cioslowski, J.; Fox, D. J. Gaussian, Inc., Wallingford CT, **2009**.

(10) Hehre, W. J.; Radom, L.; Schleyer, P. v.R.; Pople, J. A. *Ab Initio Molecular Orbital Theory*, John Wiley & Sons, NY, **1986**.

(11) Parr, R. G.; Yang, W. *Density Functional Theory of Atoms and Molecules*; Oxford University Press: New York, **1989**.

(12) (a) Perdew, J. P.; Burke, K.; Ernzerhof, M. Generalized Gradient Approximation Made Simple *Phys. Rev. Lett.* **1996**, *77*, 3865-3868; (b) Perdew, J. P.; Burke, K.; Ernzerhof, M. Generalized Gradient Approximation Made Simple *Phys. Rev. Lett.* **1997**, *78*, 1396-1396. (c) Perdew, J. P. Density-functional approximation for the correlation energy of the inhomogeneous electron gas *Phys. Rev. B* **1986**, *33*, 8822-8824.

(13) (a) Haeusermann, U.; Dolg, M.; Stoll, H.; Preuss, H.; Schwerdtfeger, P.; Pitzer, R. M. Accuracy of energy-adjusted quasirelativistic ab initio pseudopotentials *Mol. Phys.* **1993**, *78*, 1211-1224. (b) Kuechle, W.; Dolg, M.; Stoll, H.; Preuss, H. Energy-adjusted pseudopotentials for the actinides. Parameter sets and test calculations for thorium and thorium monoxide *J. Chem. Phys.* **1994**, *100*, 7535-7542. (c) Leininger, T.; Nicklass, A.; Stoll, H.; Dolg, M.; Schwerdtfeger, P. The accuracy of the pseudopotential approximation. II. A comparison of various core sizes for indium pseudopotentials in calculations for spectroscopic constants of InH, InF, and InCl *J. Chem. Phys.* **1996**, *105*, 1052-1059.

(14) (a) Ditchfield, R.; Hehre, W. J.; Pople, J. A. Self-Consistent Molecular-Orbital Methods. IX. An Extended Gaussian-Type Basis for Molecular-Orbital Studies of Organic Molecules *J. Chem. Phys.* **1971**, *54*, 724-728. (b) Hehre, W. J.; Ditchfield, R.; Pople, J. A. Self-Consistent Molecular Orbital Methods. 12. Further extensions of Gaussian-type basis sets for use in molecular-orbital studies of organic-molecules *J. Chem. Phys.* **1972**, *56*, 2257-2261. (c) Hariharan, P. C.; Pople, J. A. Accuracy of AH equilibrium geometries by single determinant molecular-orbital theory *Mol. Phys.* **1974**, *27*, 209-214. (d) Gordon, M. S. The isomers of silacyclopropane *Chem. Phys. Lett.* **1980**, *76*, 163-168. (e) Hariharan, P. C.; Pople, J. A. Influence of polarization functions on molecular-orbital hydrogenation energies *Theor. Chim. Acta* **1973**, *28*, 213-222.

(15) (a) Peng, C.; Ayala, P. Y.; Schlegel, H. B.; Frisch, M. J. Using redundant internal coordinates to optimize equilibrium geometries and transition states *J. Comp. Chem.* **1996**, *17*, 49-56. (b) Peng, C.; Schlegel, H. B. Combining Synchronous Transit and Quasi-Newton Methods for Finding Transition States *Israel J. Chem.* **1993**, *33*, 449-454.

(16) (a) McClean, A. D.; Chandler, G. S. Contracted Gaussian basis sets for molecular calculations. I. Second row atoms, *Z*=11-18 *J. Chem. Phys.* **1980**, *72*, 5639-5648. (b) Krishnan, R.; Binkley, J. S.; Seeger, R.; Pople, J. A. Self-consistent molecular orbital methods. XX. A basis set for correlated wave functions *J. Chem. Phys.* **1980**, *72*, 650-654. (c) Wachters, A. J. H. Gaussian Basis Set for Molecular Wavefunctions Containing Third-Row Atoms *J. Chem. Phys.* **1970**, *52*, 1033-1036. (d) Hay, P. J. Gaussian basis sets for molecular calculations - representation of 3D orbitals in transition-metal atoms *J. Chem. Phys.* **1977**, *66*, 4377-4384. (e) Raghavachari, K.; Trucks, G. W. Highly correlated systems: Excitation energies of first row transition metals Sc-Cu *J. Chem. Phys.* **1989**, *91*, 1062-1065. (f) Binning Jr., R. C.; Curtiss, L. A. Compact contracted basis-sets for 3rd-row atoms - Ga-Kr *J. Comp. Chem.* **1990**, *11*, 1206-1216. (g) McGrath, M. P.; Radom, L. Extension of Gaussian-1 (G1) theory to bromine-containing molecules *J. Chem. Phys.* **1991**, *94*, 511-516. (h) Curtiss, L. A.; McGrath, M. P.; Blaudeau, J.-P.; Davis, N. E.; Binning Jr., R. C.; Radom, L. Extension of Gaussian-2 theory to molecules containing third-row atoms Ga-Kr *J. Chem. Phys.*, **1995**, *103*, 6104-6113. (i) Clark, T.; Chandrasekhar, J.; Spitznagel, G. W.; Schleyer, P. v. R. Efficient diffuse function-augmented basis-sets for anion calculations. 3. The 3-21+G basis set for 1st-row elements, Li-F *J. Comp. Chem.* **1983**, *4*, 294-301. (j) Frisch, M. J.; Pople, J. A.; Binkley, J. S. Self-Consistent Molecular Orbital Methods. 25. Supplementary Functions for Gaussian Basis Sets *J. Chem. Phys.* **1984**, *80*, 3265-3269.

(17) Grimme, S.; Antony, J.; Ehrlich, S.; Krieg, H. A consistent and accurate ab initio parameterization of density functional dispersion correction (DFT-D) for the 94 elements H-Pu *J. Chem. Phys.* **2010**, *132*, 154104.

(18) (a) Becke, A. D.; Johnson, E. R. A density-functional model of the dispersion interaction *J. Chem. Phys.* **2005**, *122*, 154101. (b) Johnson, E. R.; Becke, A. D. A post-Hartree-Fock model of intermolecular interactions *J. Chem. Phys.* **2005**, *123*, 24101. (c) Johnson, E. R.; Becke, A. D. A post-Hartree-Fock model of intermolecular interactions: Inclusion of higher-order corrections *J. Chem. Phys.* **2006**, *124*, 174104.

## NMR Spectra

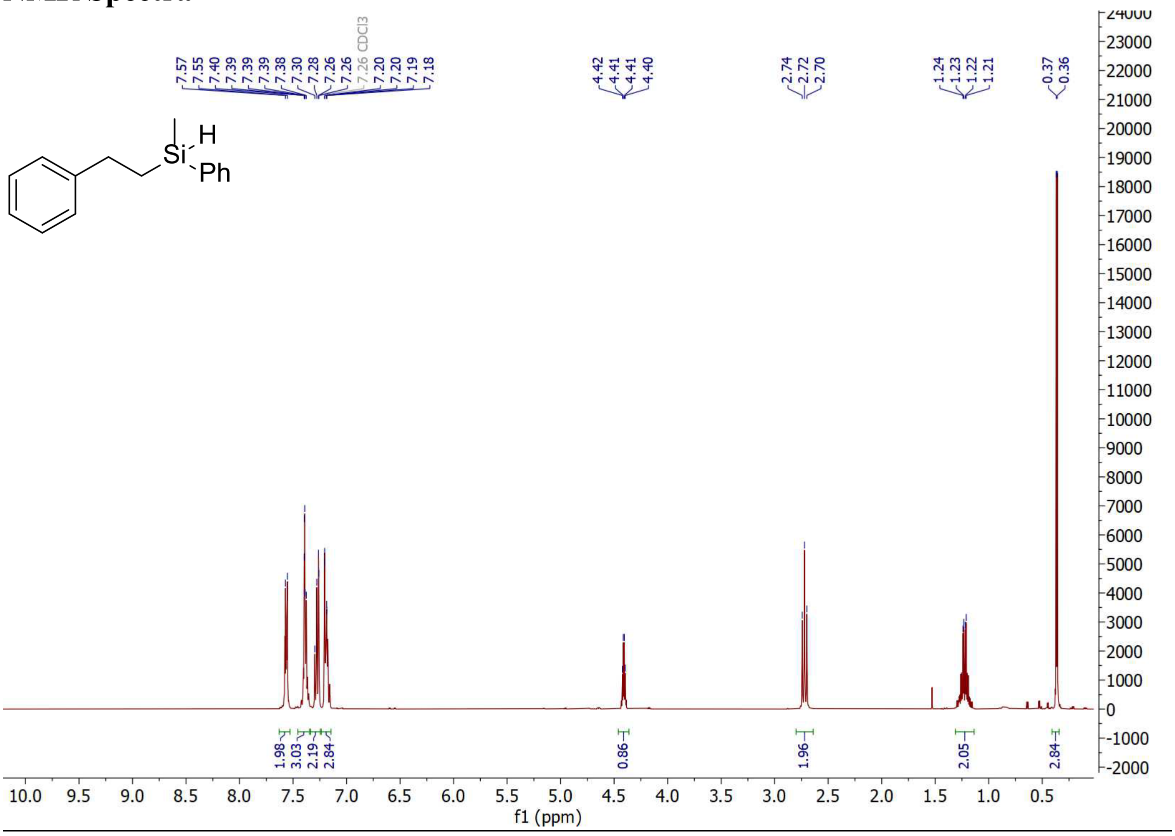

**Figure S13.** $^{1}$H-NMR spectrum of **2a**

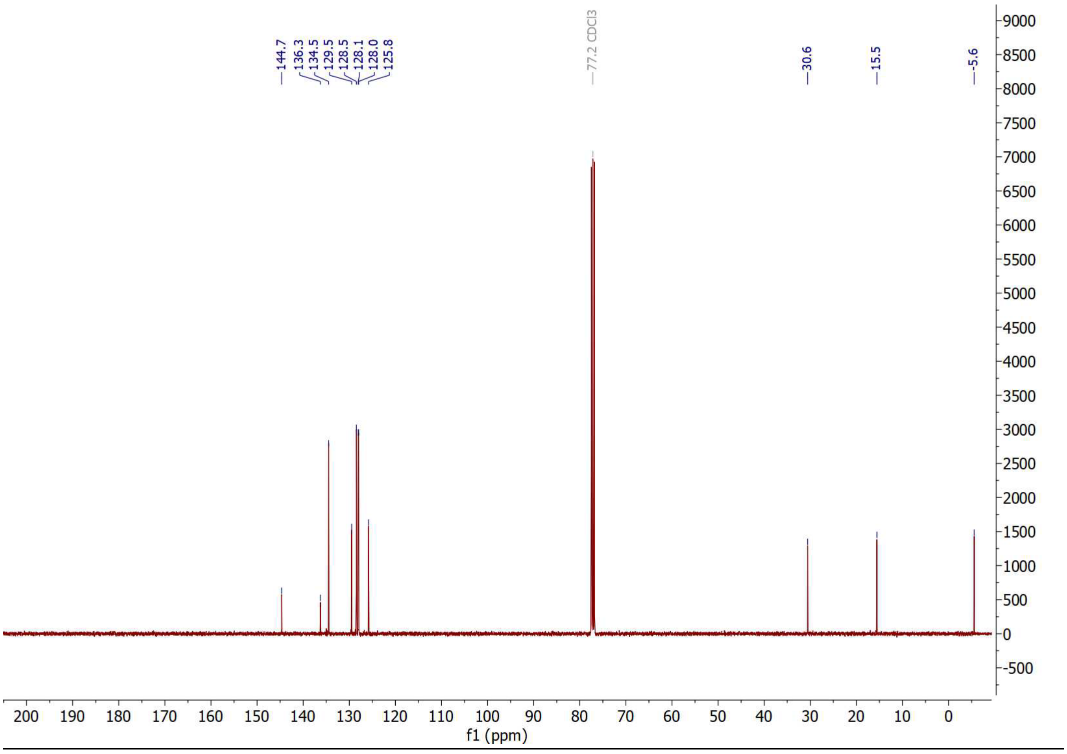

**Figure S14.** $^{13}C\{^{1}H\}$-NMR spectrum of **2a**

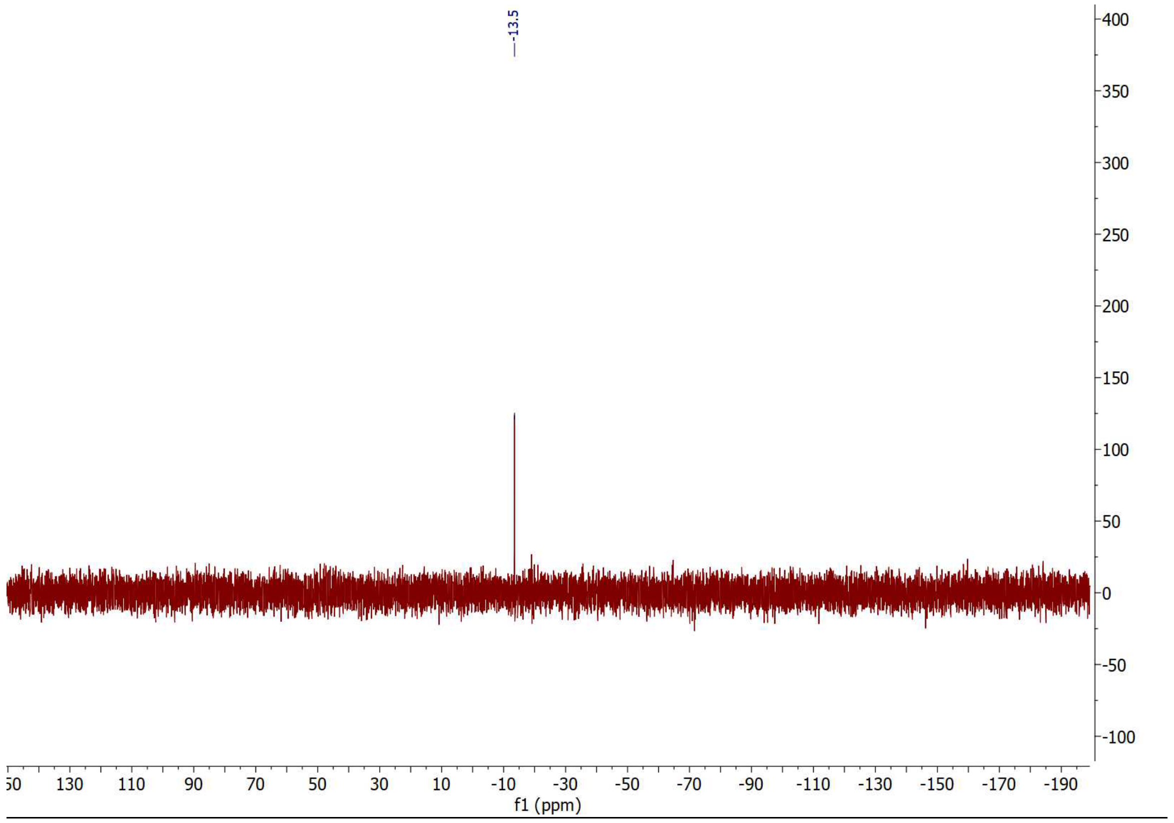

**Figure S15.** $^{29}Si\{^{1}H\}$-NMR spectrum of **2a**

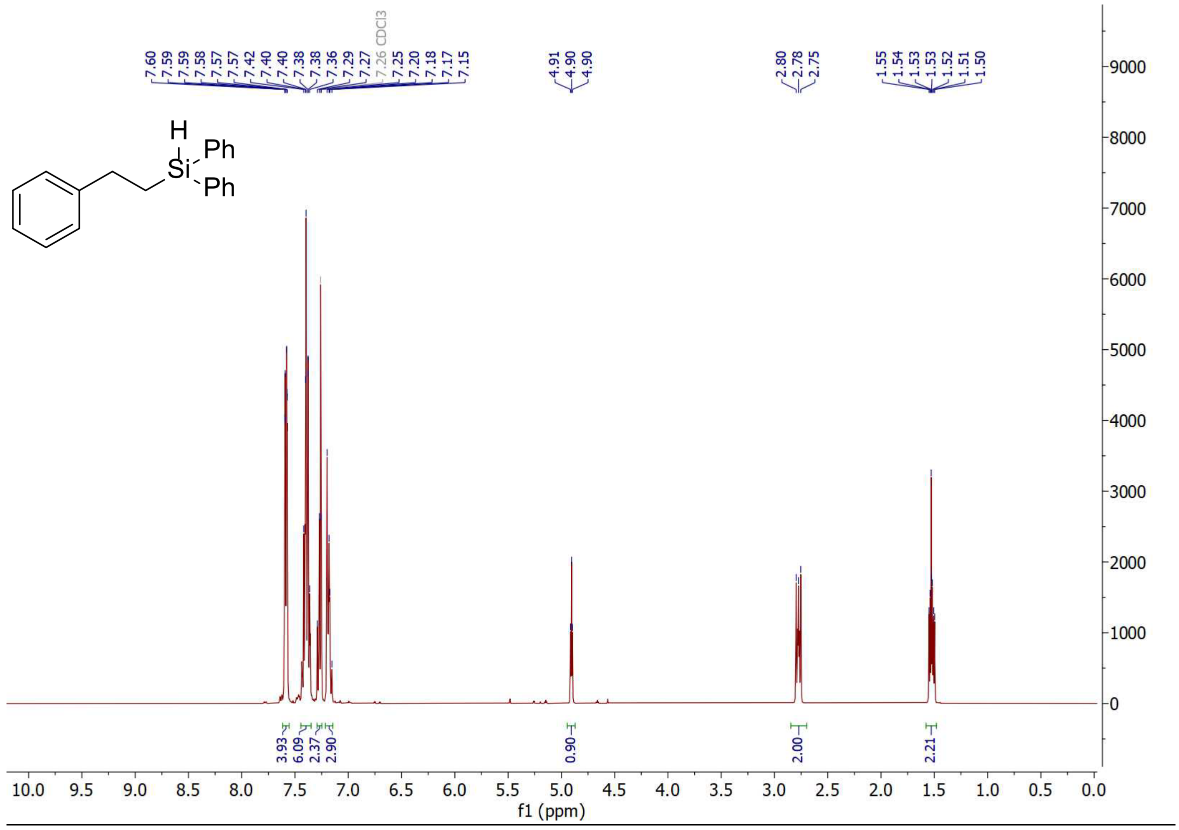

**Figure S16.** $^{1}H$-NMR spectrum of **2c**

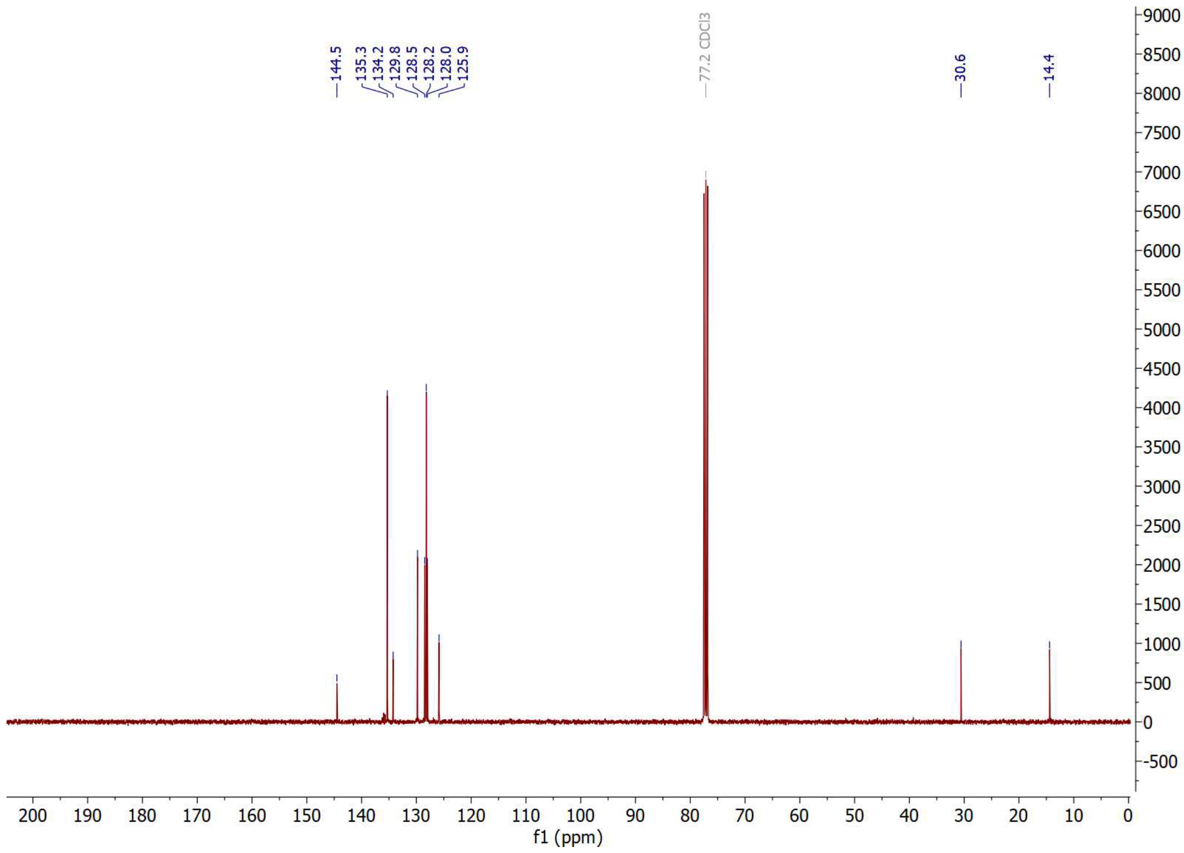


**Figure S17.** $^{13}C\{^{1}H\}$-NMR spectrum of **2c**

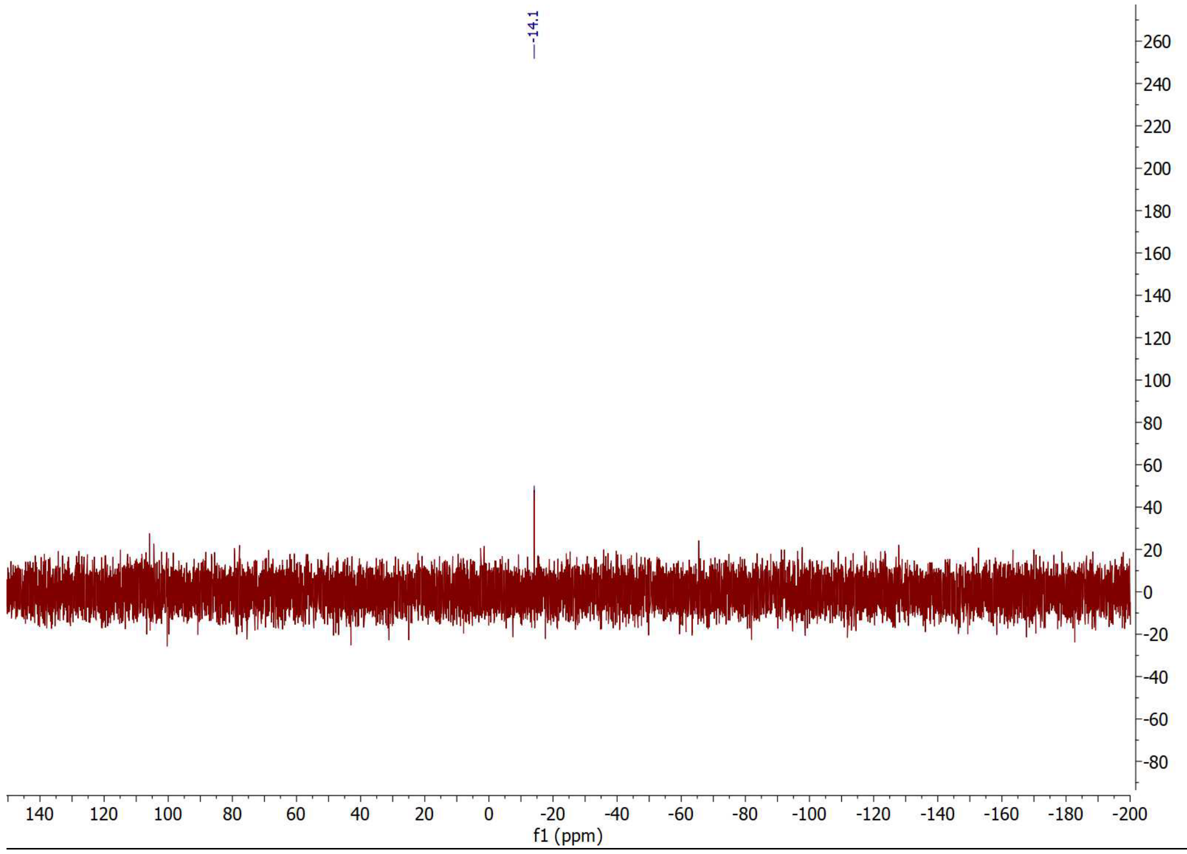


**Figure S18.** $^{29}Si\{^{1}H\}$-NMR spectrum of **2c**

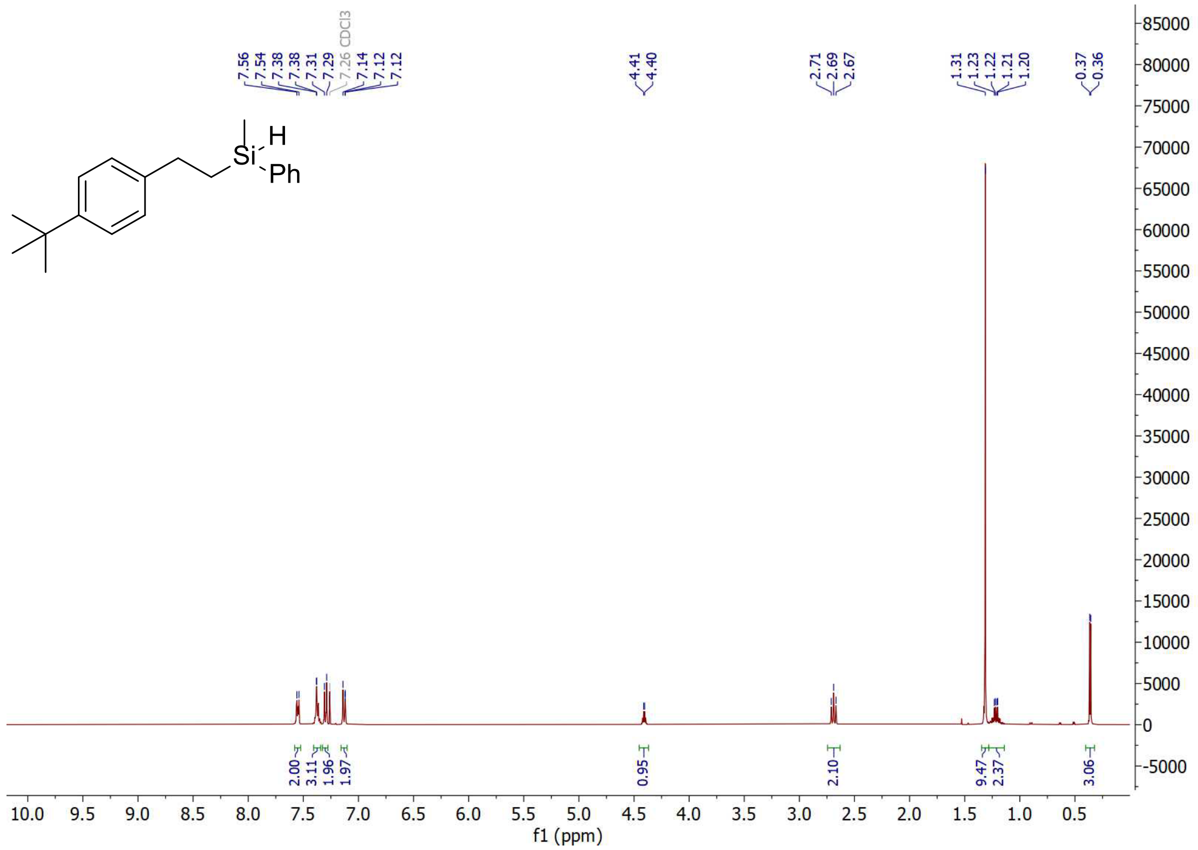


**Figure S19.** $^{1}$H-NMR spectrum of **2d**

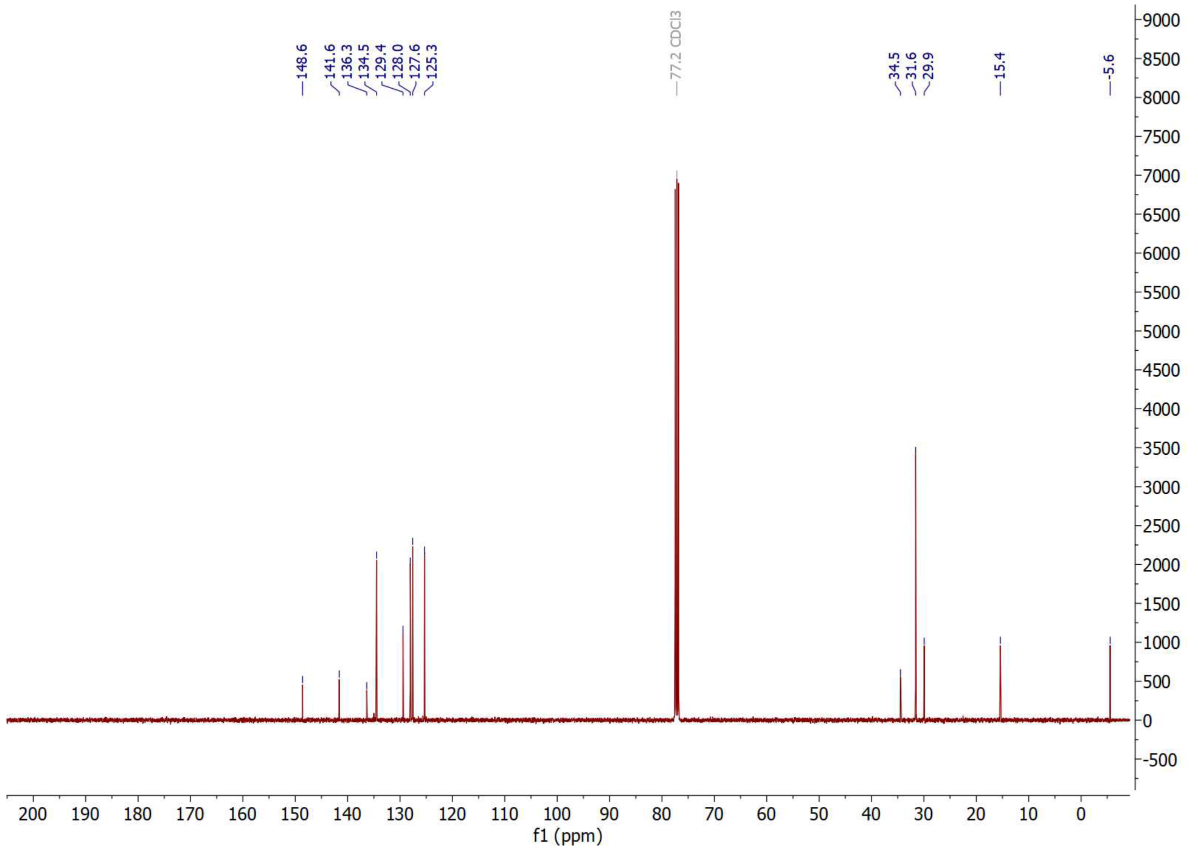


**Figure S20.** $^{13}$C{$^{1}$H}-NMR spectrum of **2d**

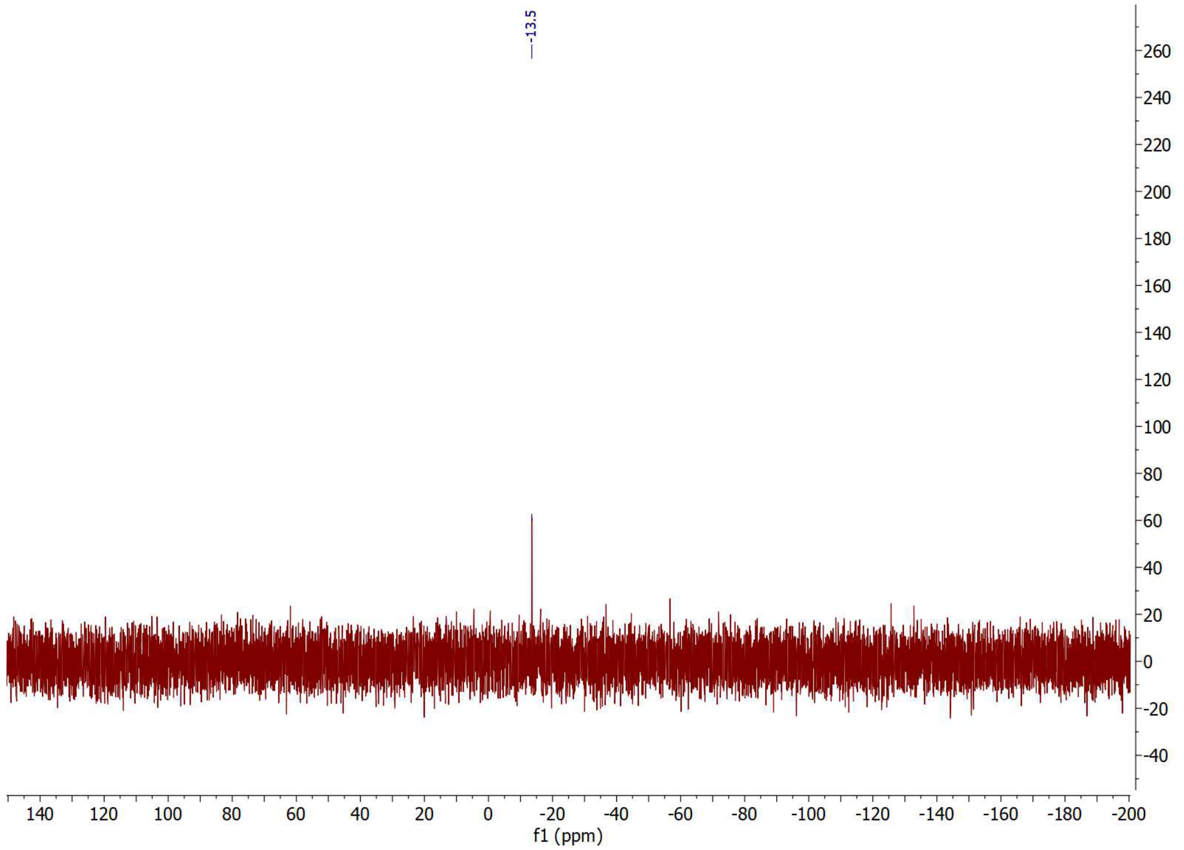

**Figure S21.** $^{29}Si\{^{1}H\}$-NMR spectrum of **2d**

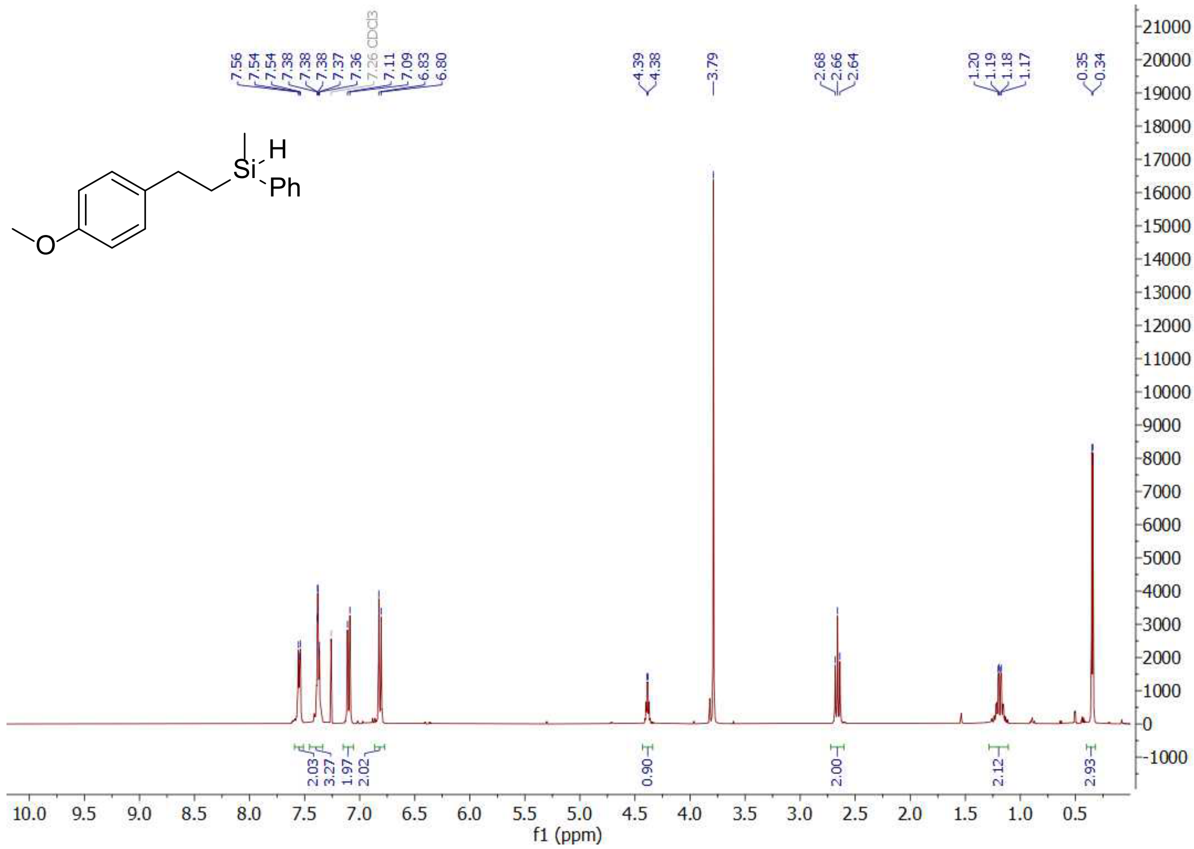

**Figure S22.** $^{1}H$-NMR spectrum of **4e**

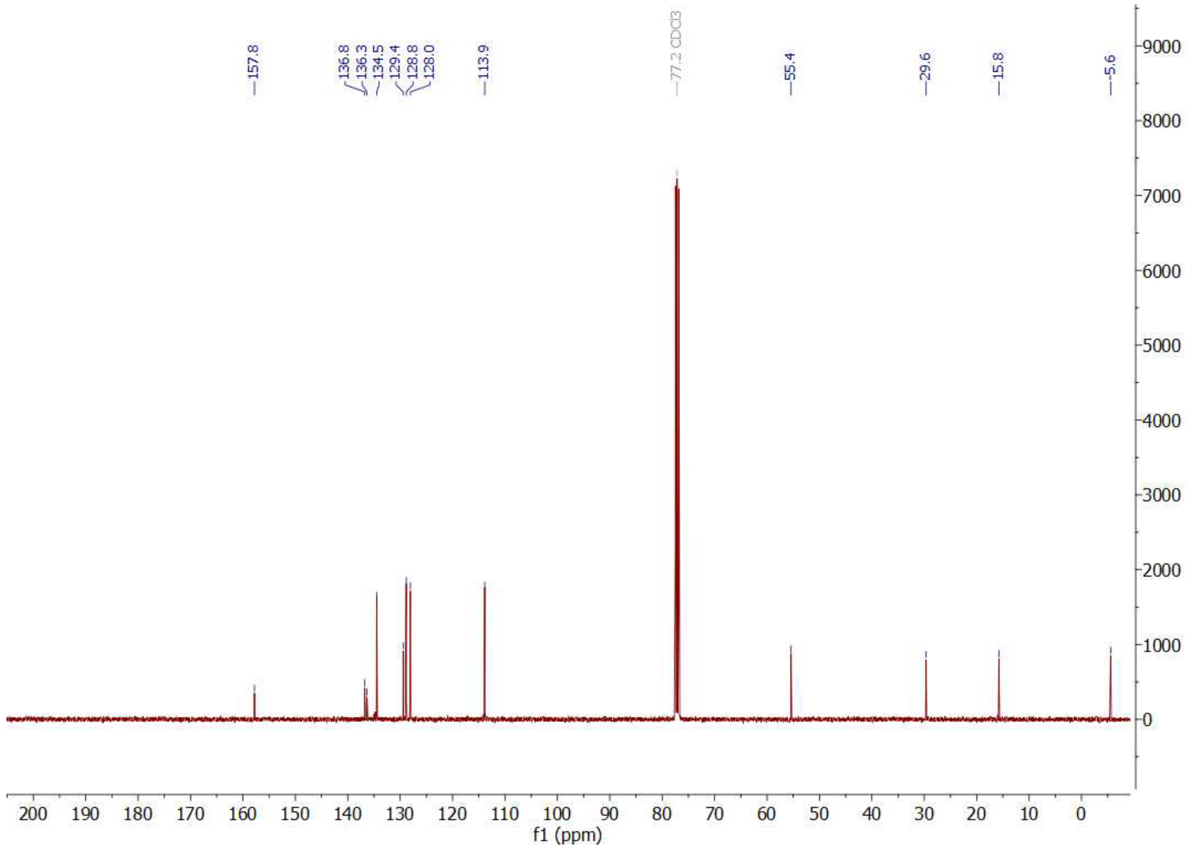


**Figure S23.** $^{13}C\{^{1}H\}$-NMR spectrum of **2e**

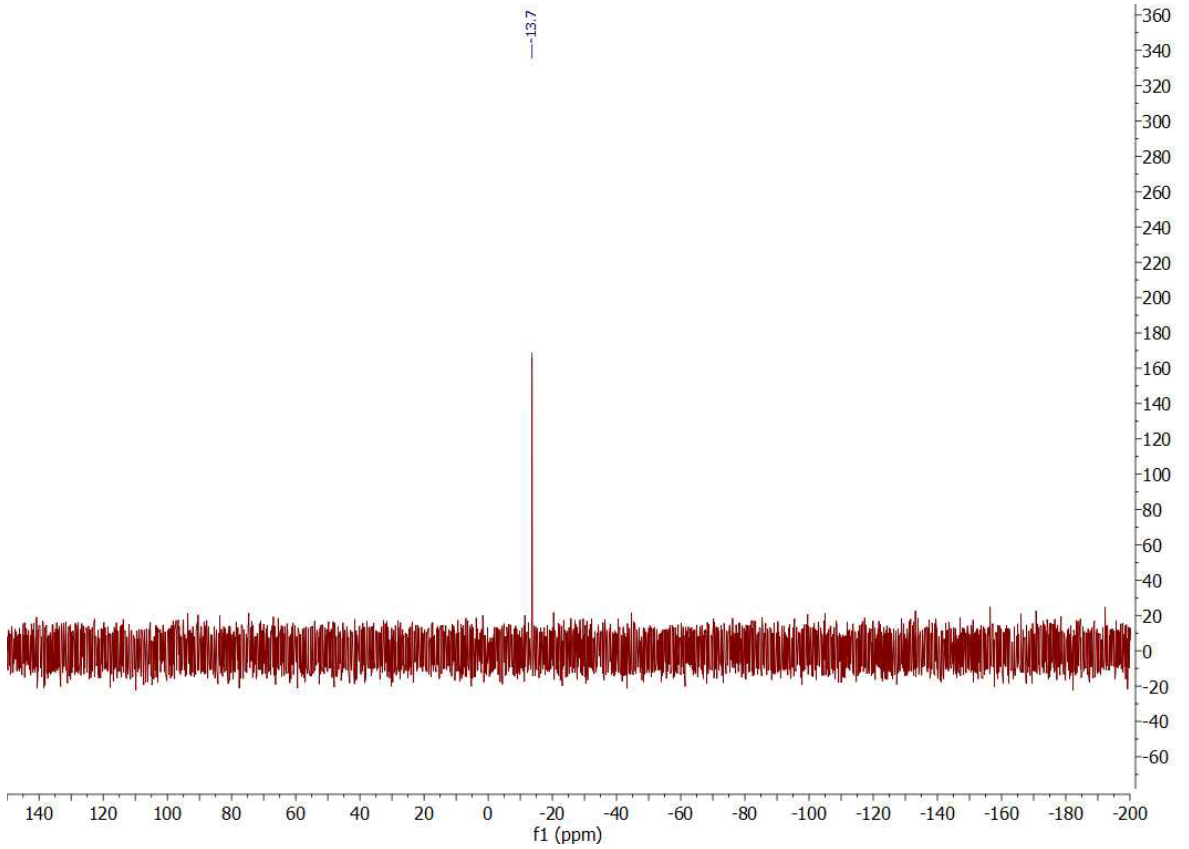


**Figure S24.** $^{29}Si\{^{1}H\}$-NMR spectrum of **2e**

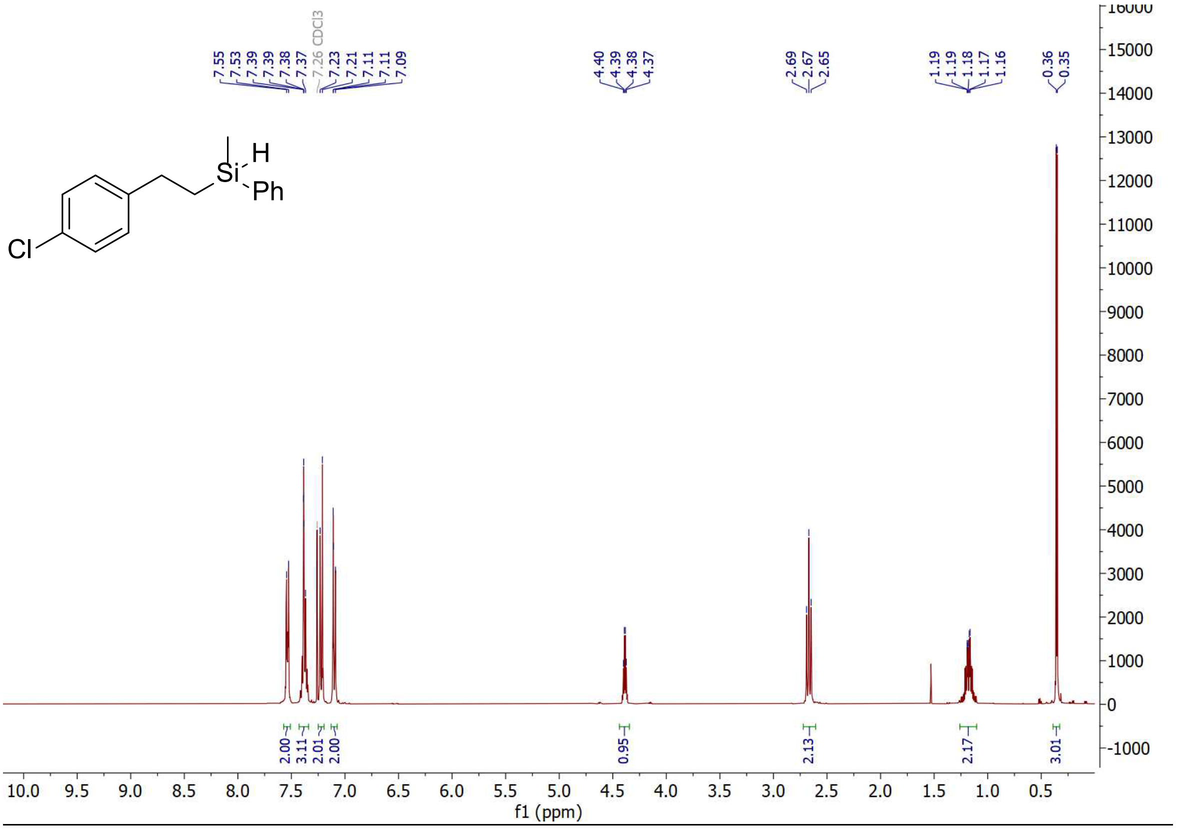


**Figure S25.** $^{1}$H-NMR spectrum of **2f**

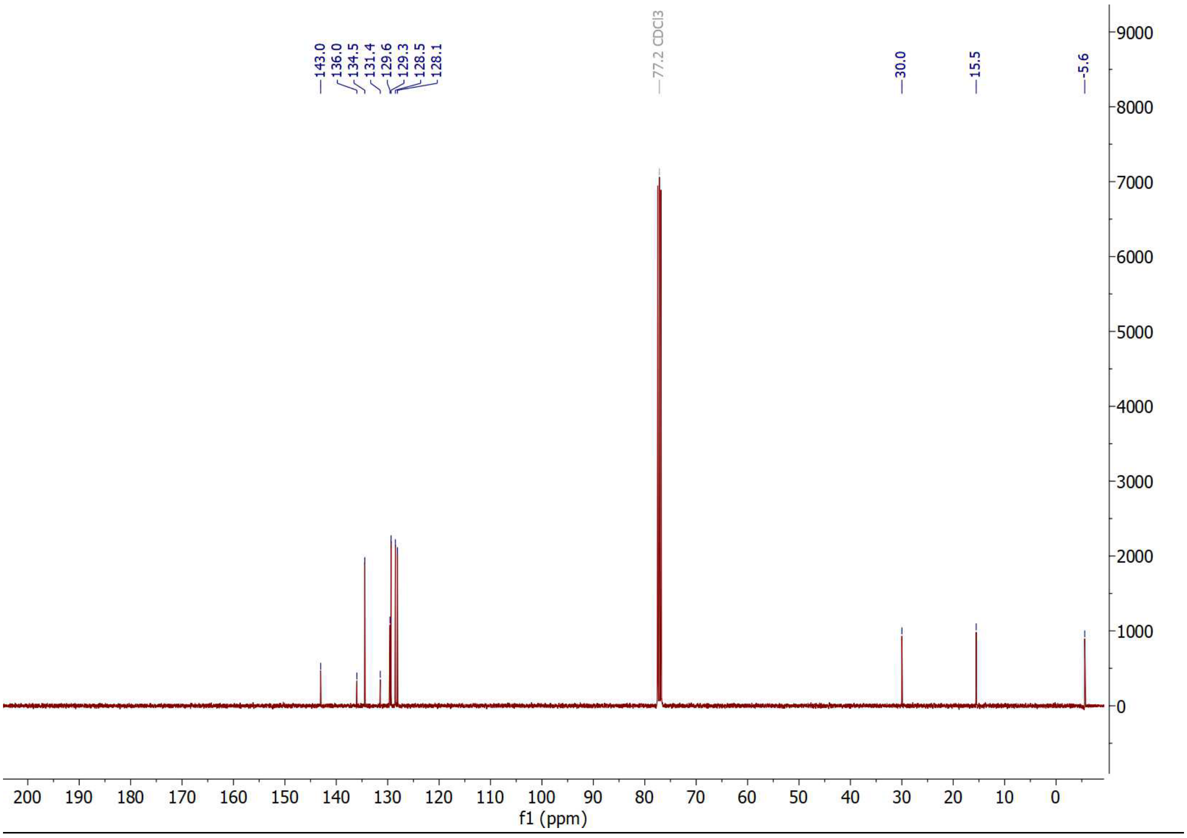


**Figure S26.** $^{13}C\{^{1}H\}$-NMR spectrum of **2f**

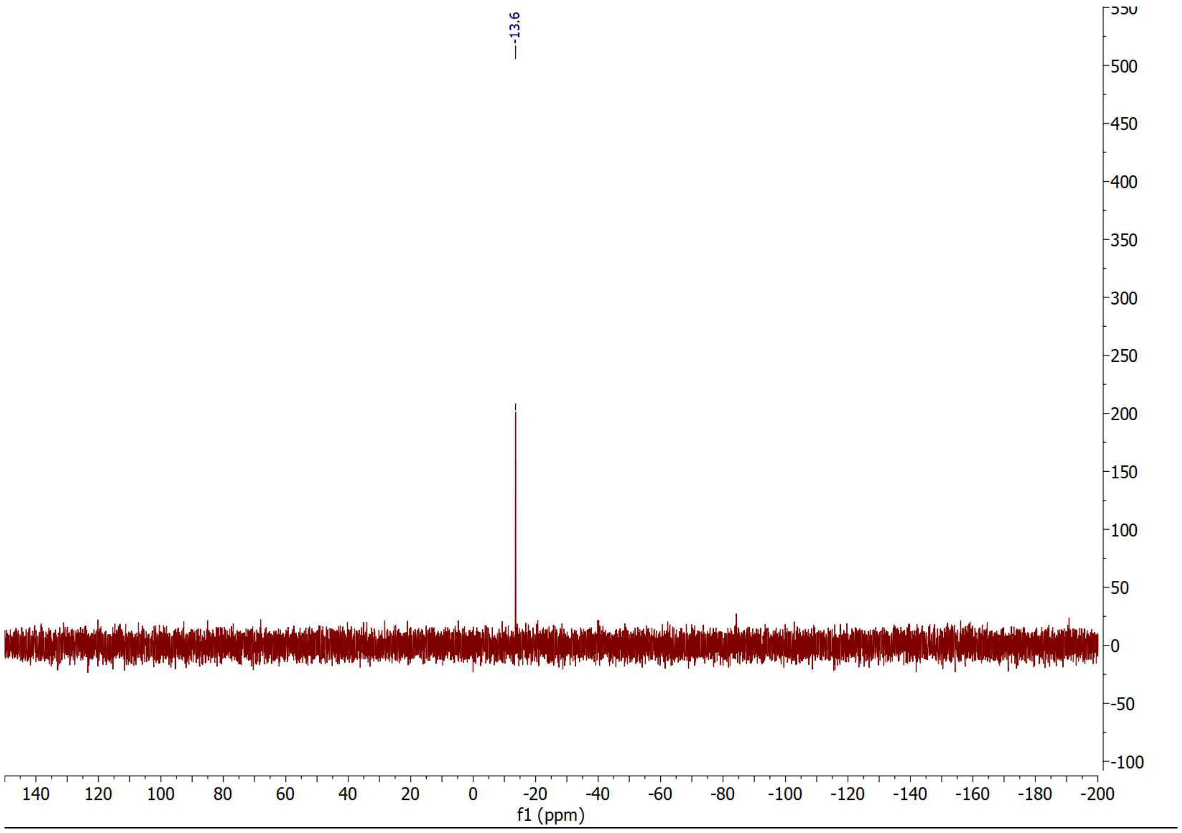


**Figure S27.** $^{29}Si\{^{1}H\}$-NMR spectrum of **2f**

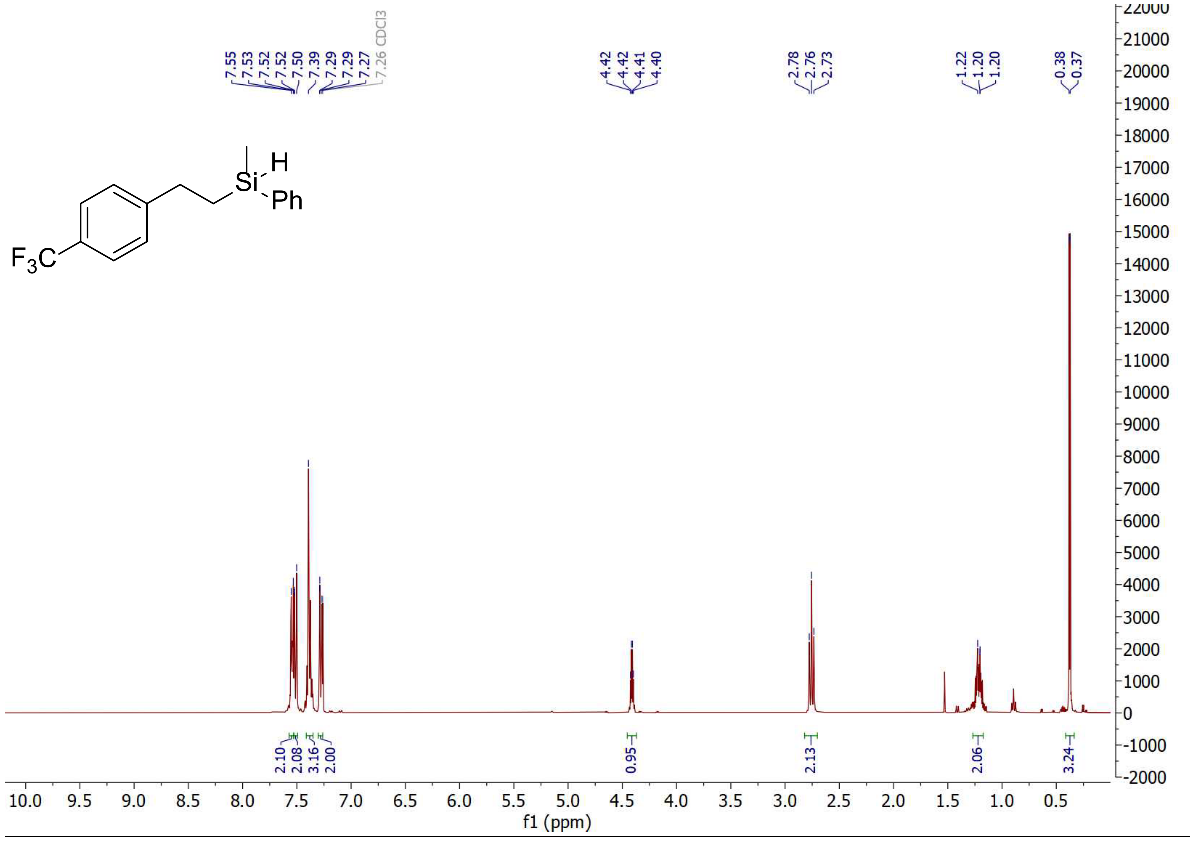


**Figure S28.** $^{1}$H-NMR spectrum of **2g**

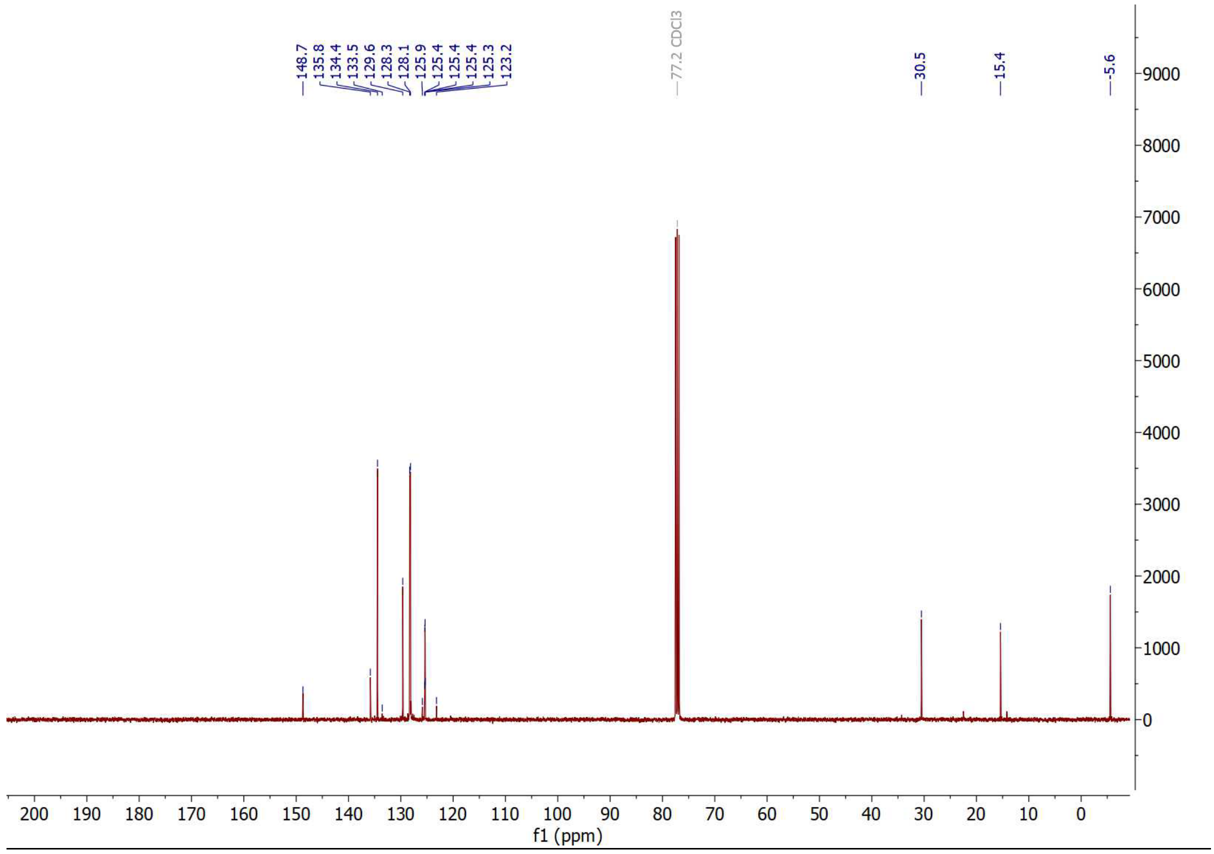


**Figure S29.** $^{13}C\{^{1}H\}$-NMR spectrum of **2g**

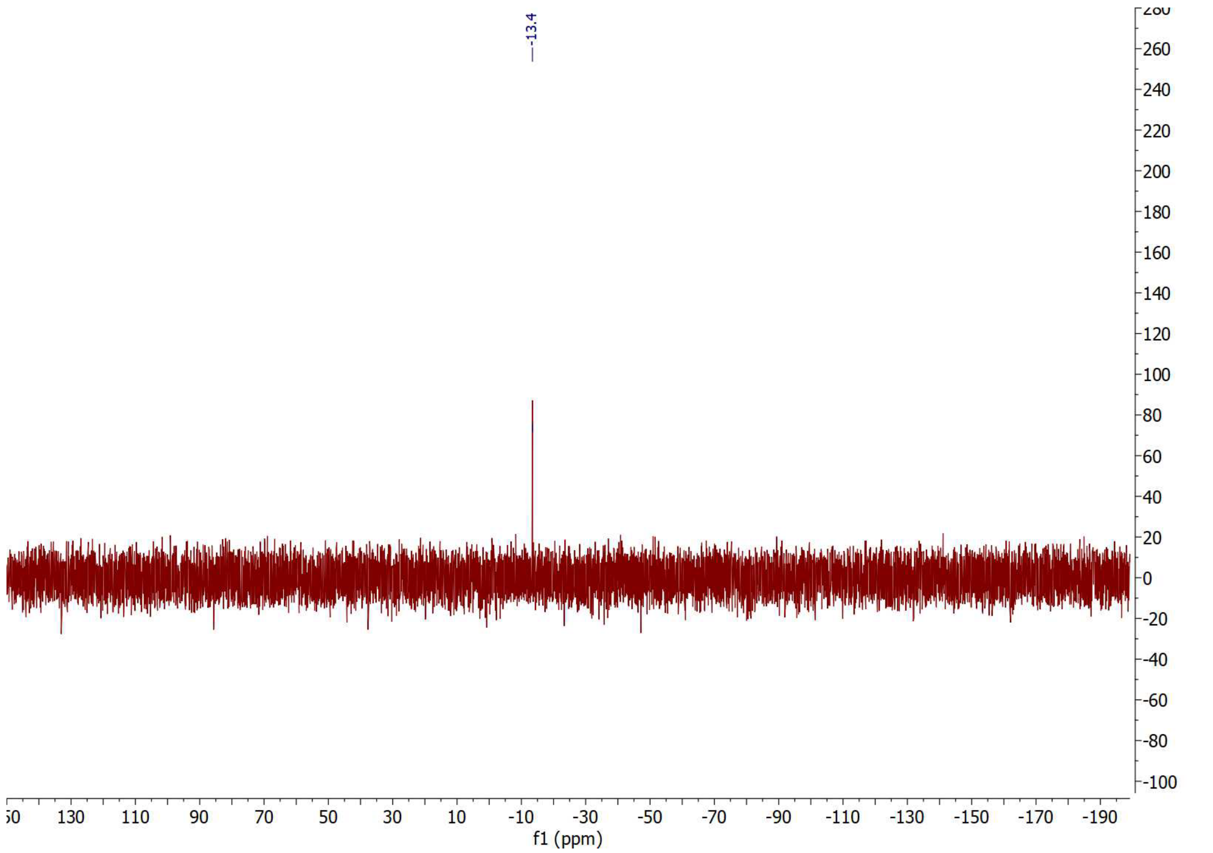


**Figure S30.** $^{29}Si\{^{1}H\}$-NMR spectrum of **2g**

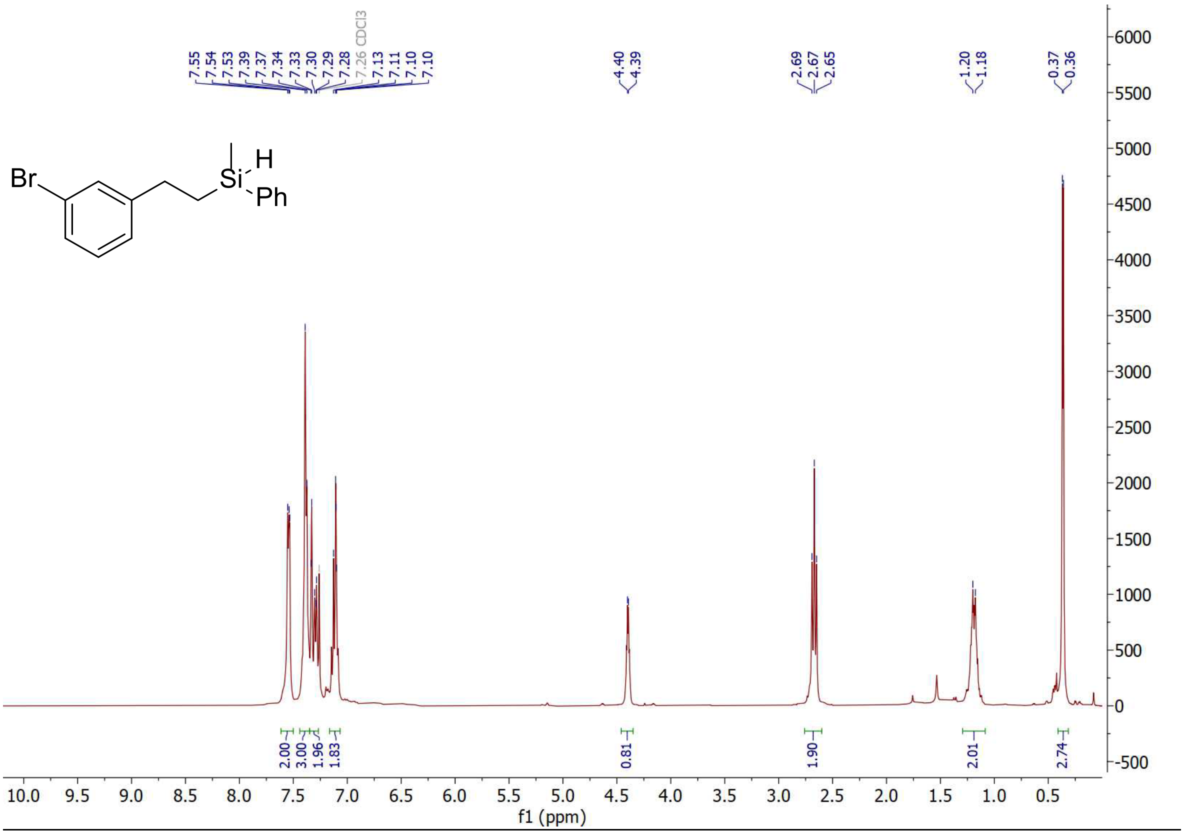

**Figure S31.** $^{1}$H-NMR spectrum of **2h**

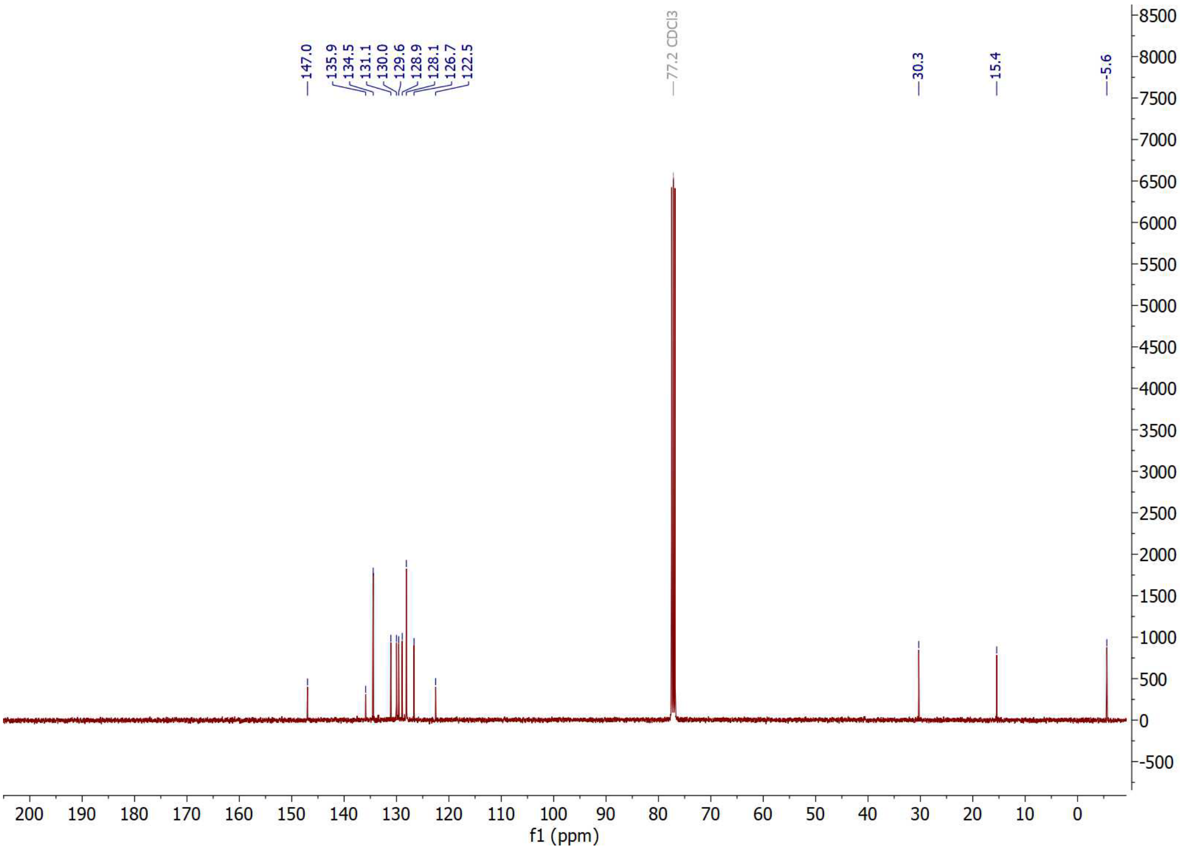

**Figure S32.** $^{13}C\{^{1}H\}$-NMR spectrum of **2h**

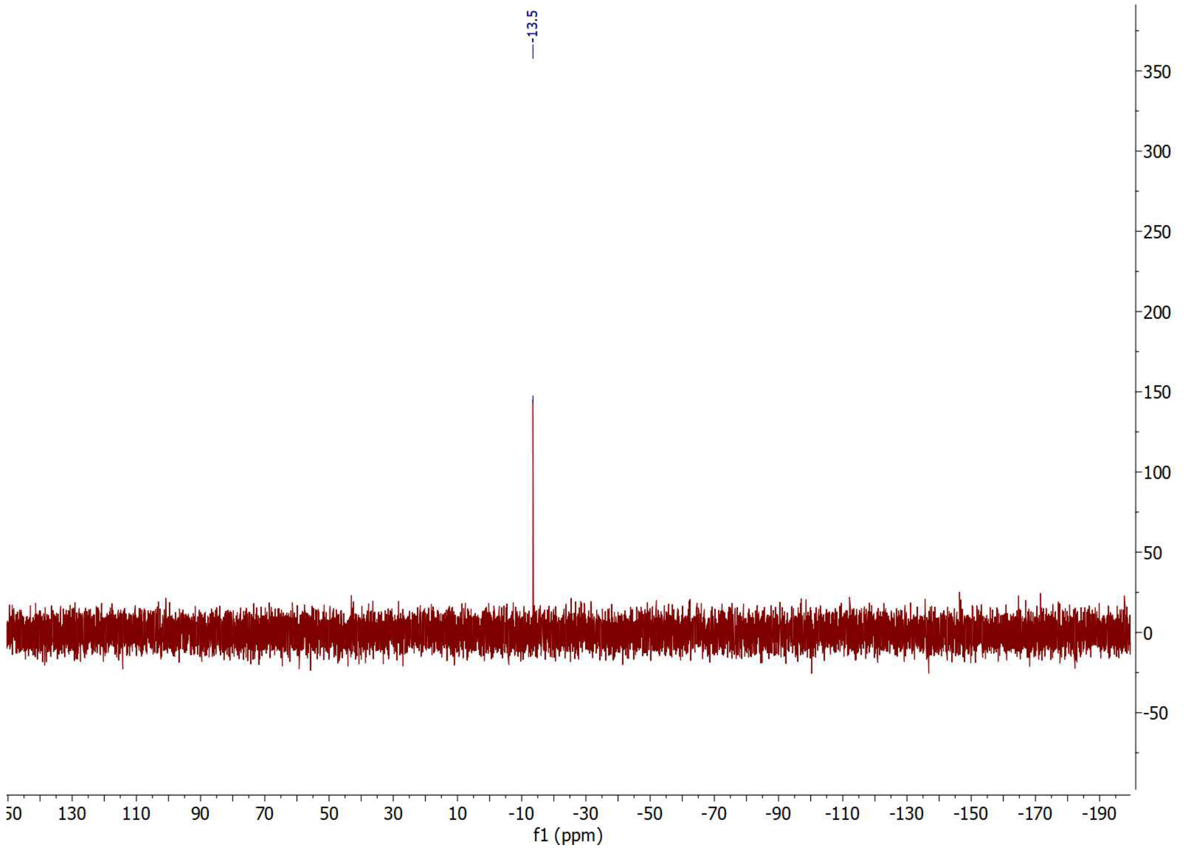


**Figure S33.** $^{29}Si\{^{1}H\}$-NMR spectrum of **2h**

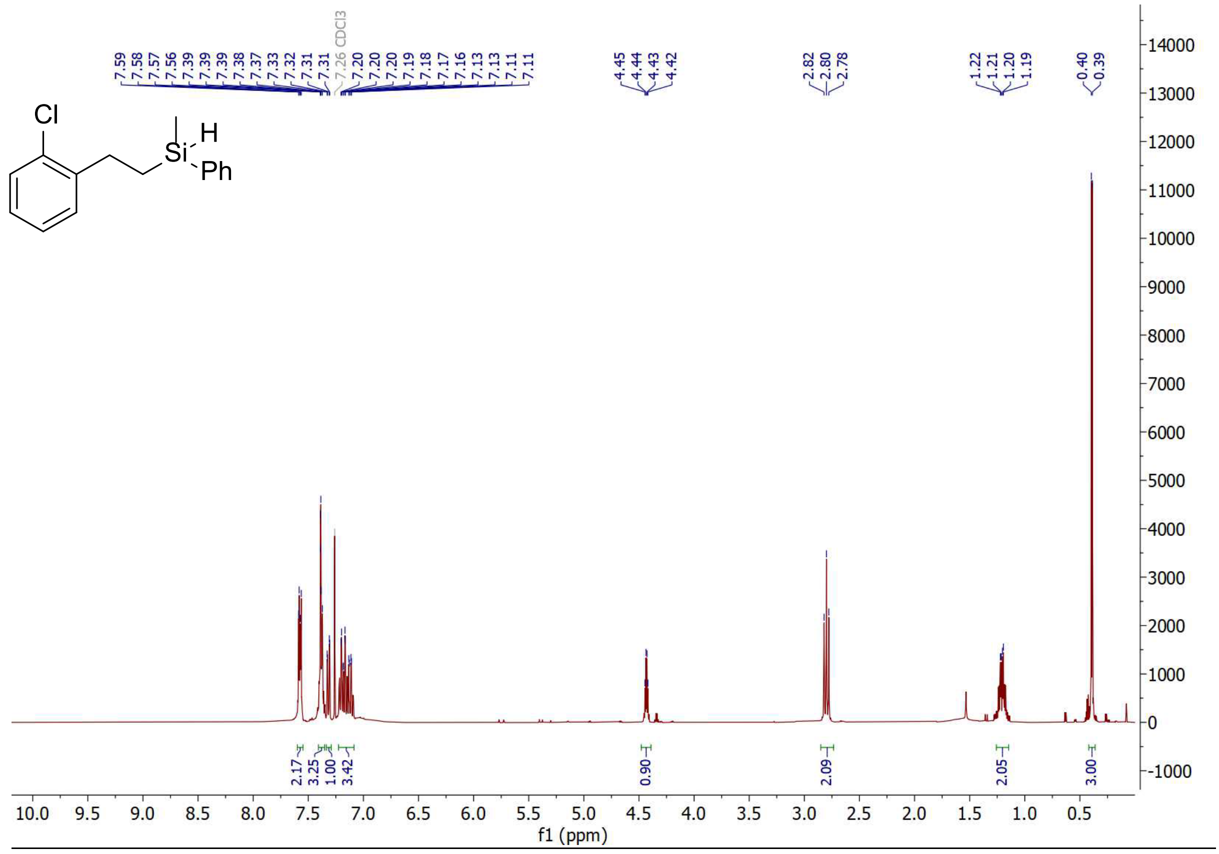


**Figure S34.** $^{1}H$-NMR spectrum of **2i**

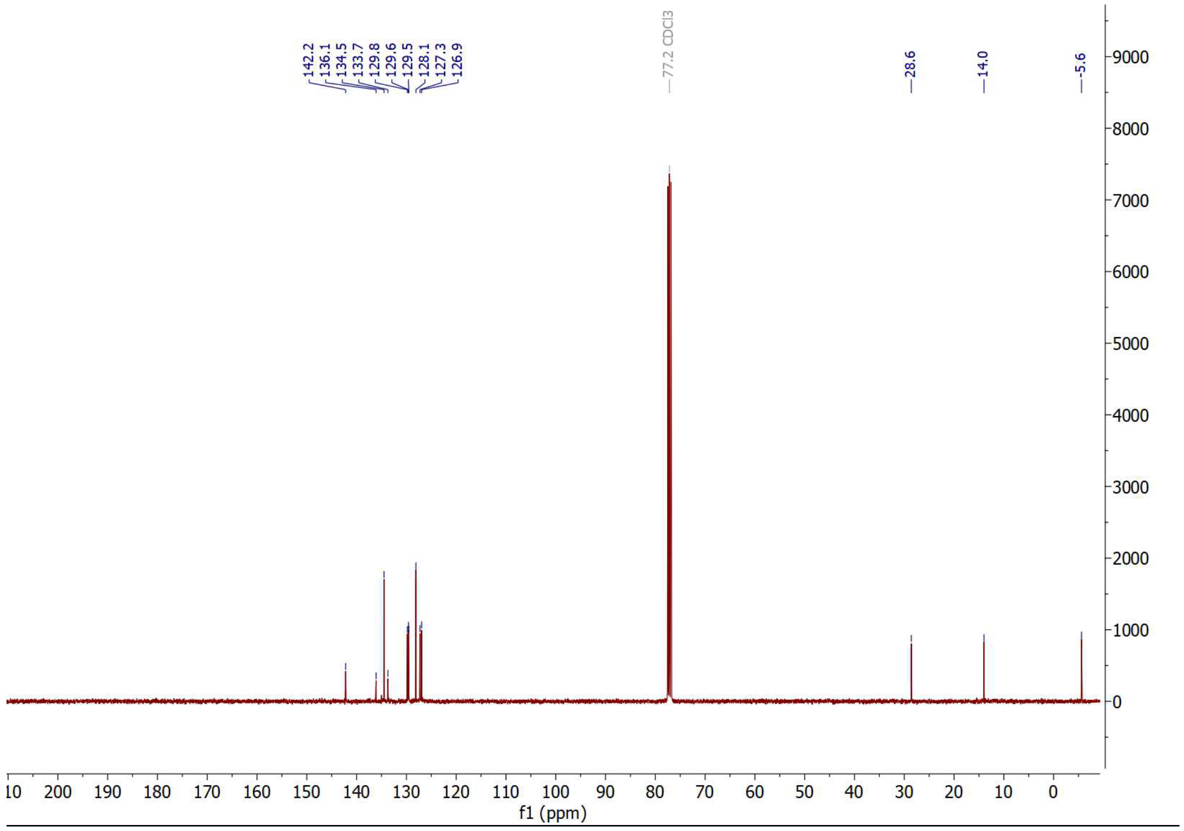


**Figure S35.** $^{13}C\{^1H\}$-NMR spectrum of **2i**

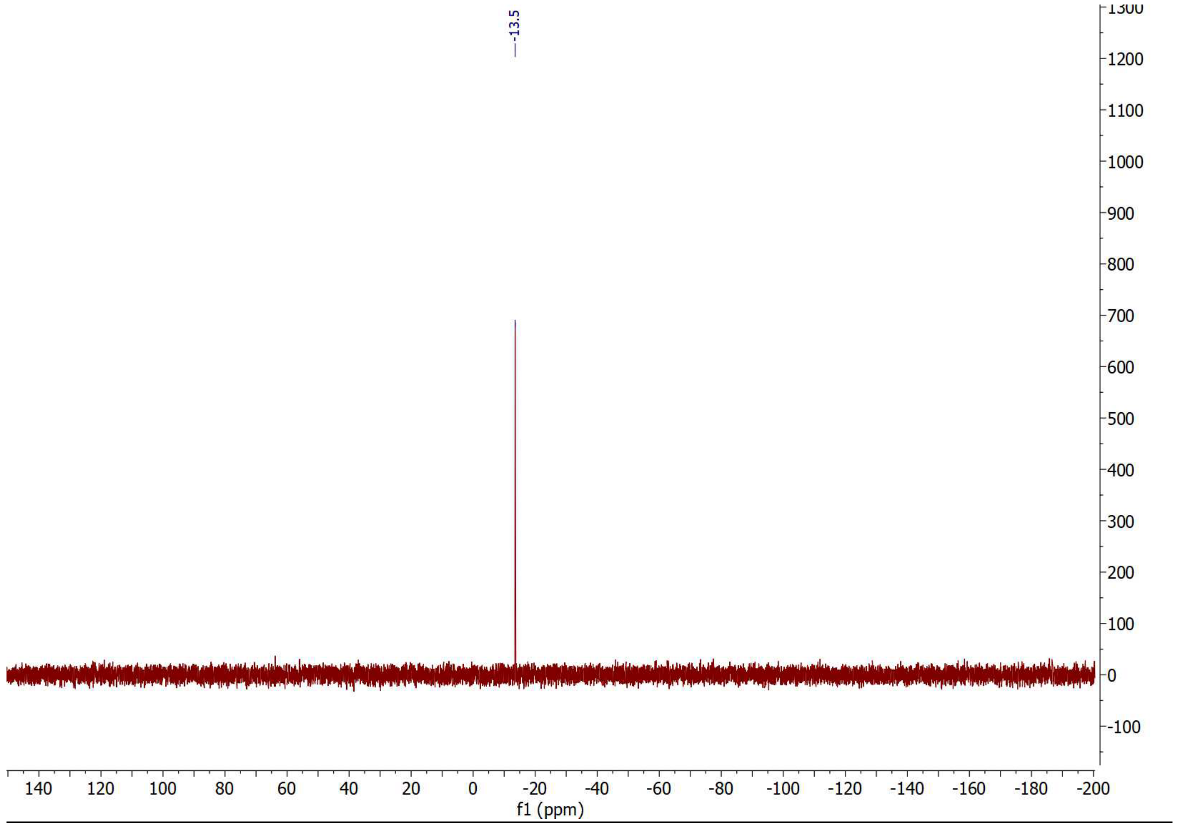


**Figure S36.** $^{29}Si\{^1H\}$-NMR spectrum of **2i**

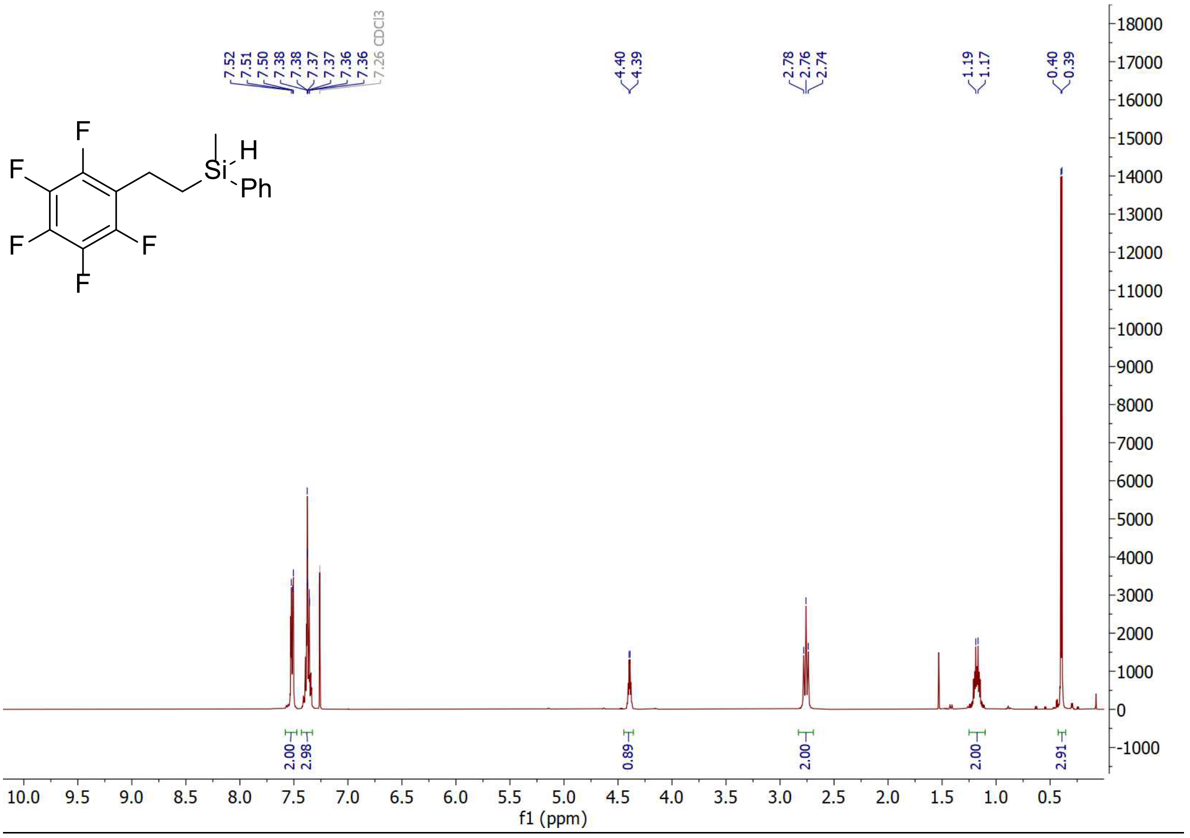


**Figure S37.** $^{1}$H-NMR spectrum of **2j**

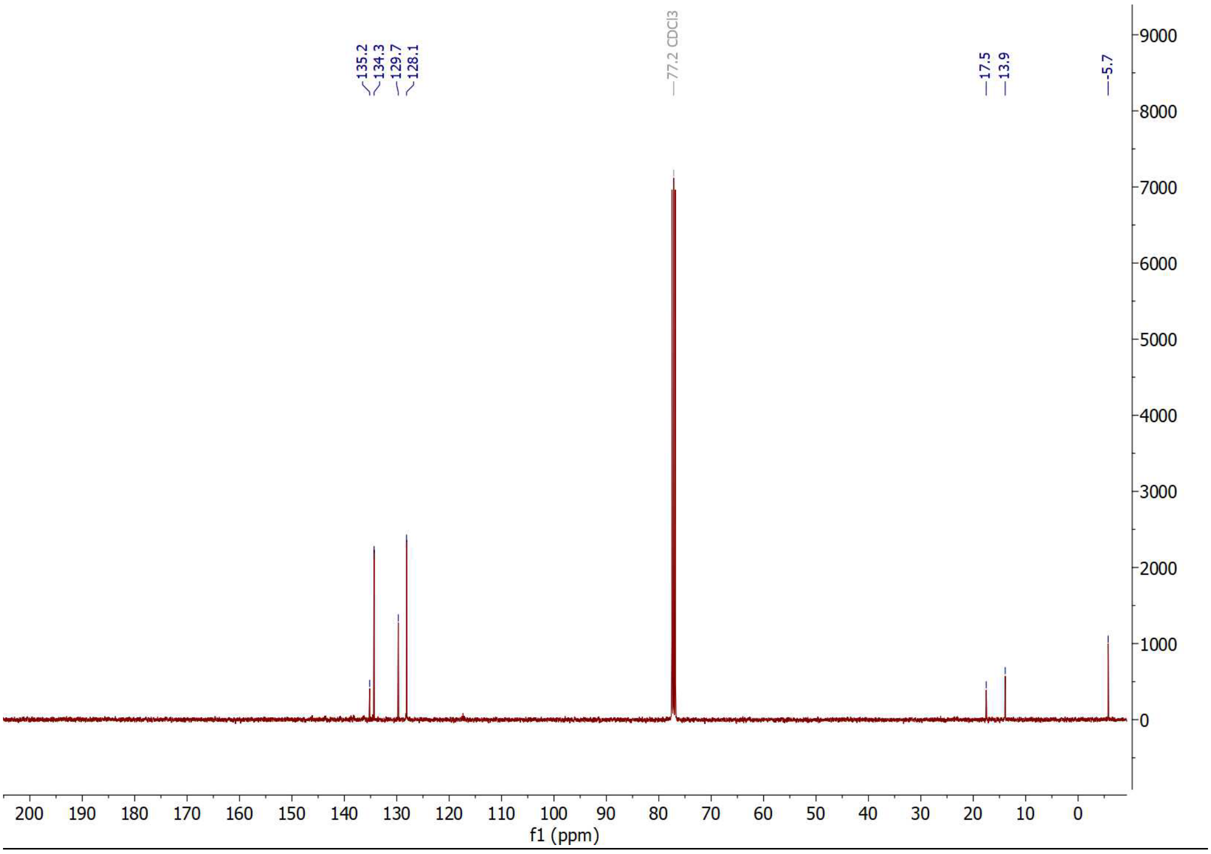


**Figure S38.** $^{13}C\{^{1}H\}$-NMR spectrum of **2j**

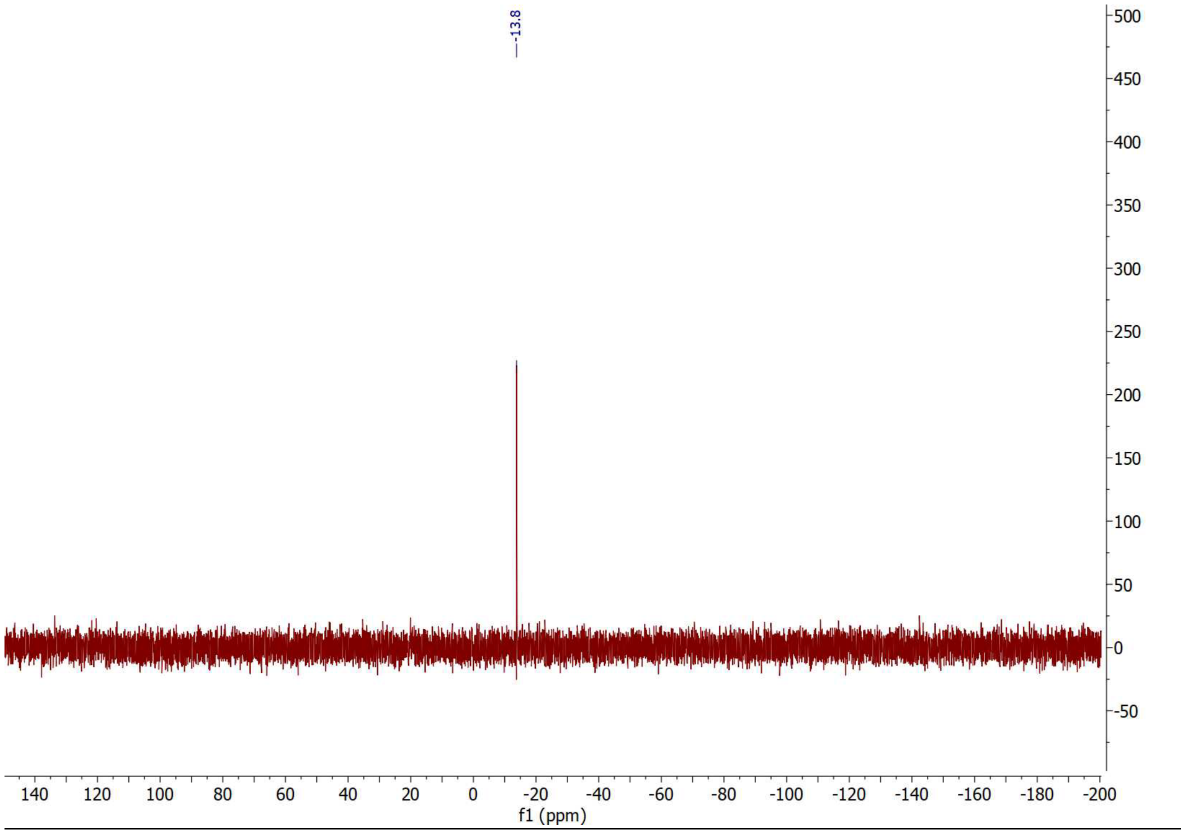


**Figure S39.** $^{29}Si\{^{1}H\}$-NMR spectrum of **2j**

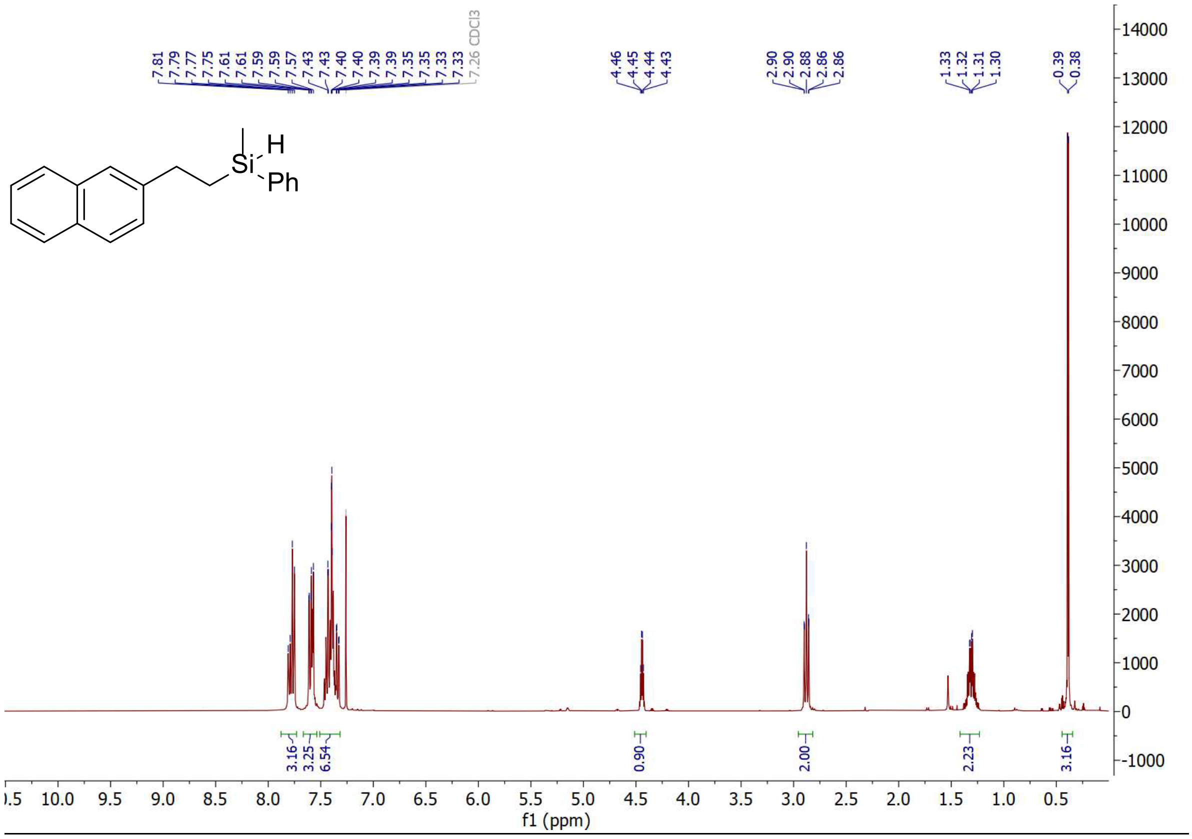


**Figure S40.** $^{1}H$-NMR spectrum of **2k**

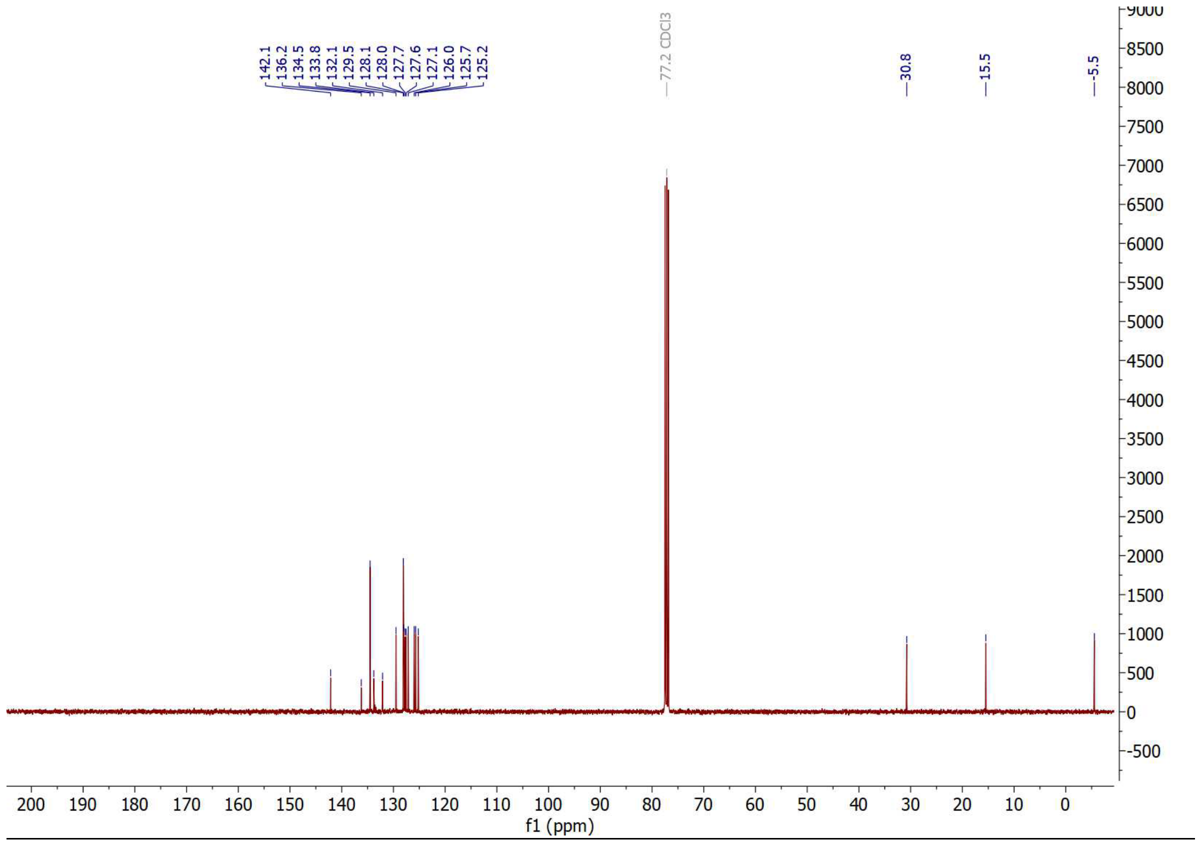


**Figure S41.** $^{13}C\{^{1}H\}$-NMR spectrum of **2k**

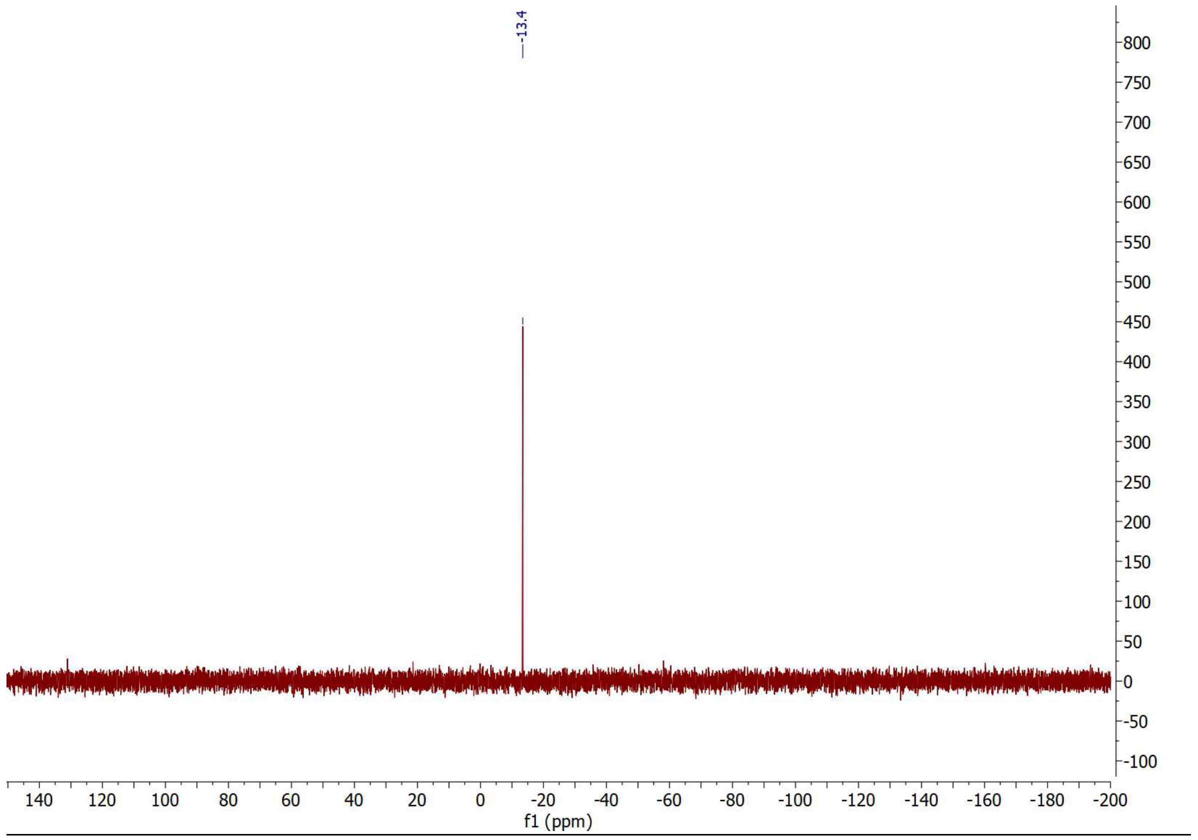


**Figure S42.** $^{29}Si\{^{1}H\}$-NMR spectrum of **2k**

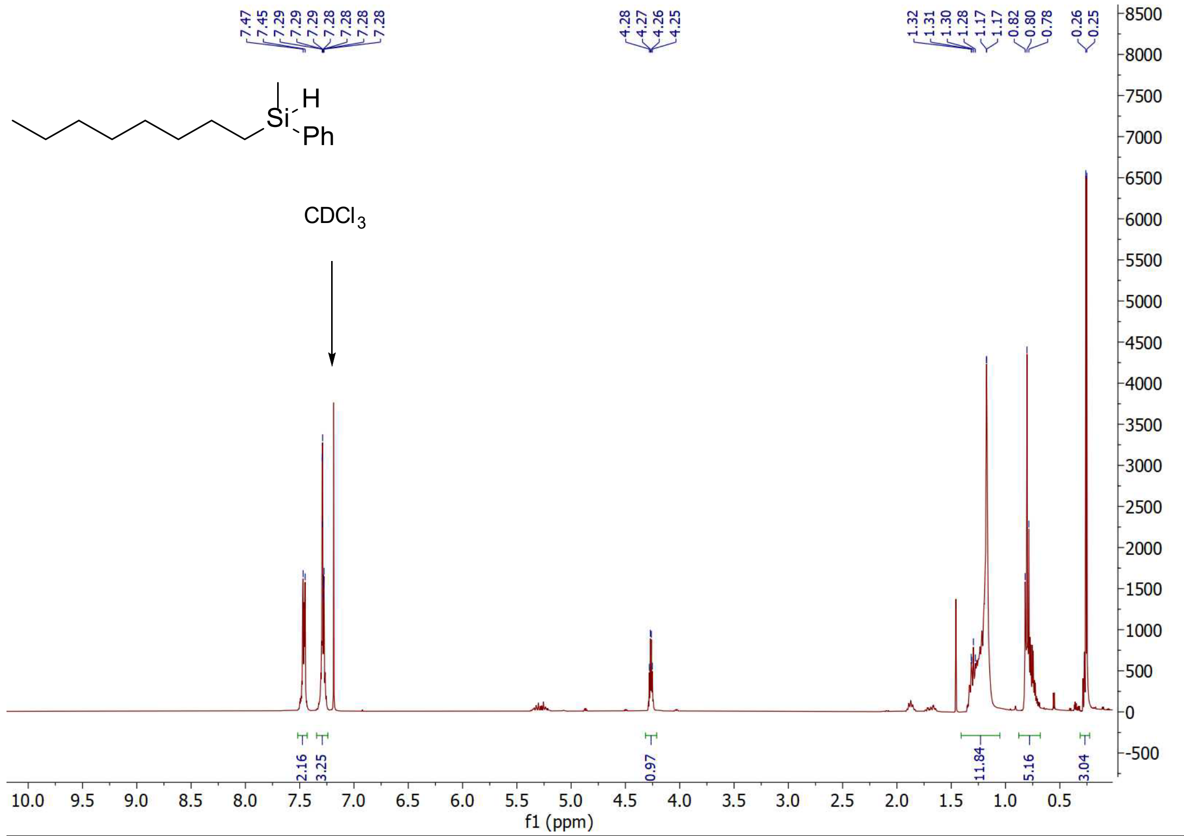


**Figure S43.** $^{1}$H-NMR spectrum of **2m**

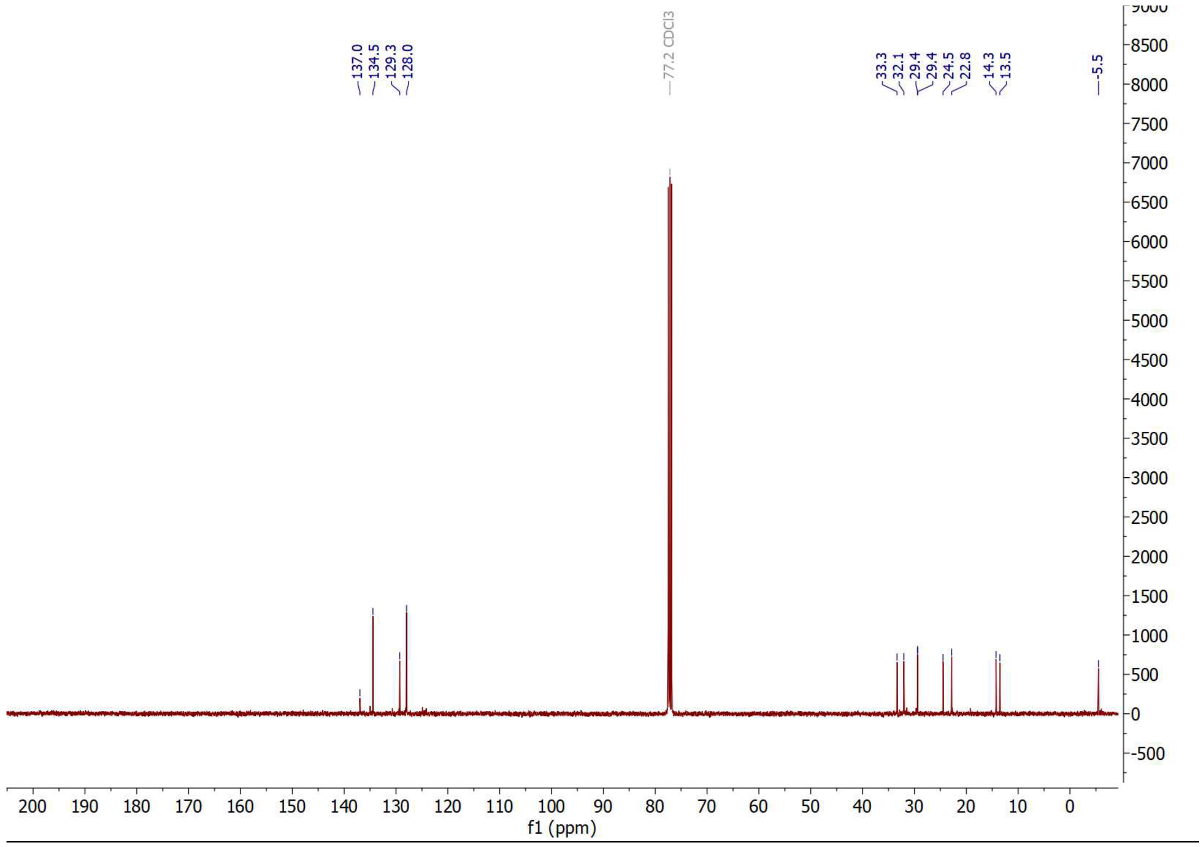


**Figure S44.** $^{13}C\{^{1}H\}$-NMR spectrum of **2m**

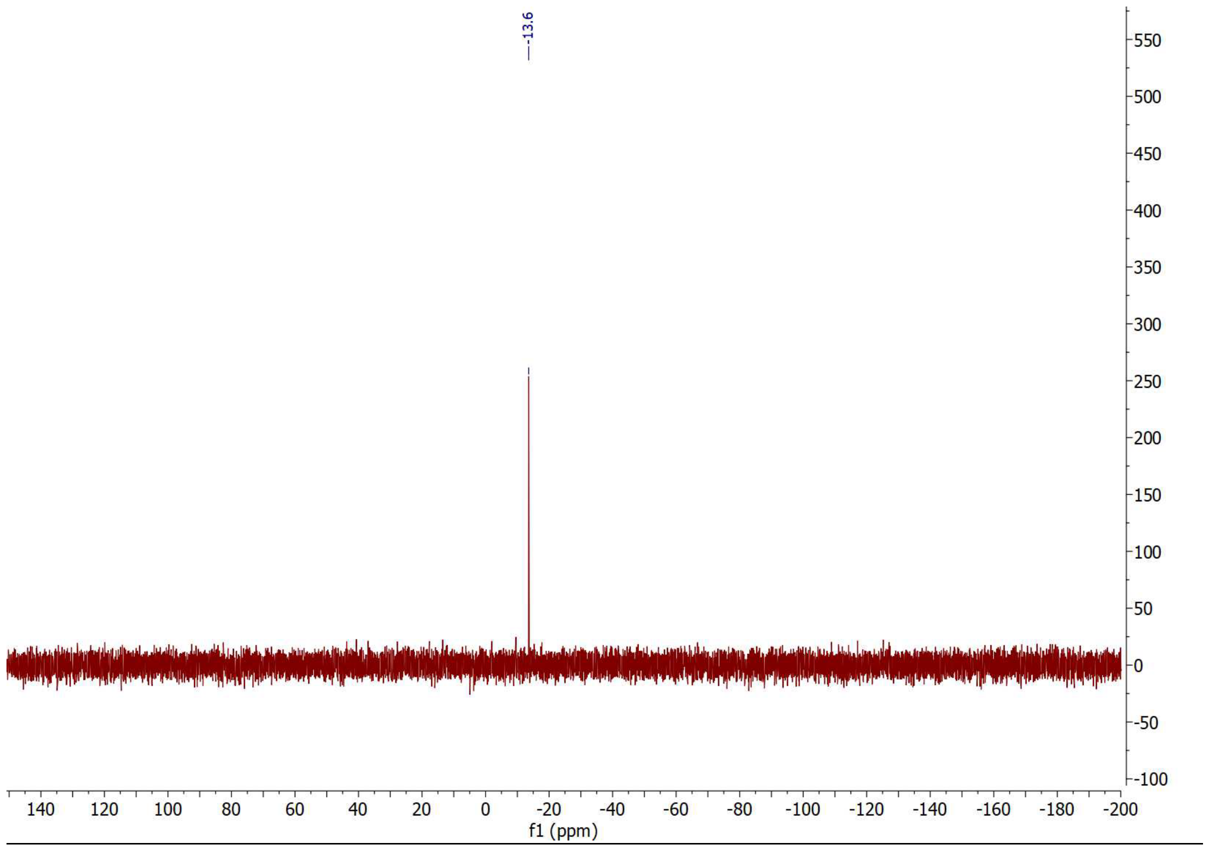

**Figure S45.** $^{29}Si\{^{1}H\}$-NMR spectrum of **2m**

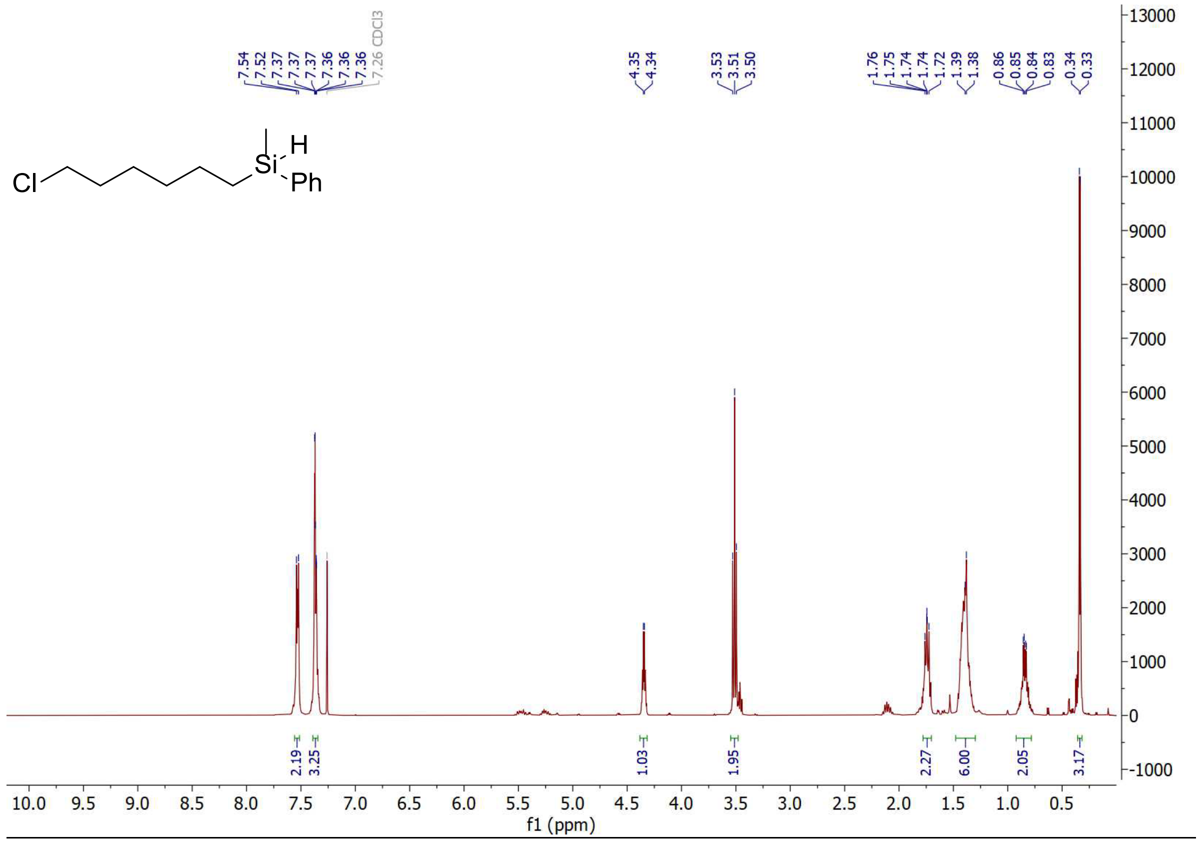

**Figure S46.** $^{1}H$-NMR spectrum of **2n**

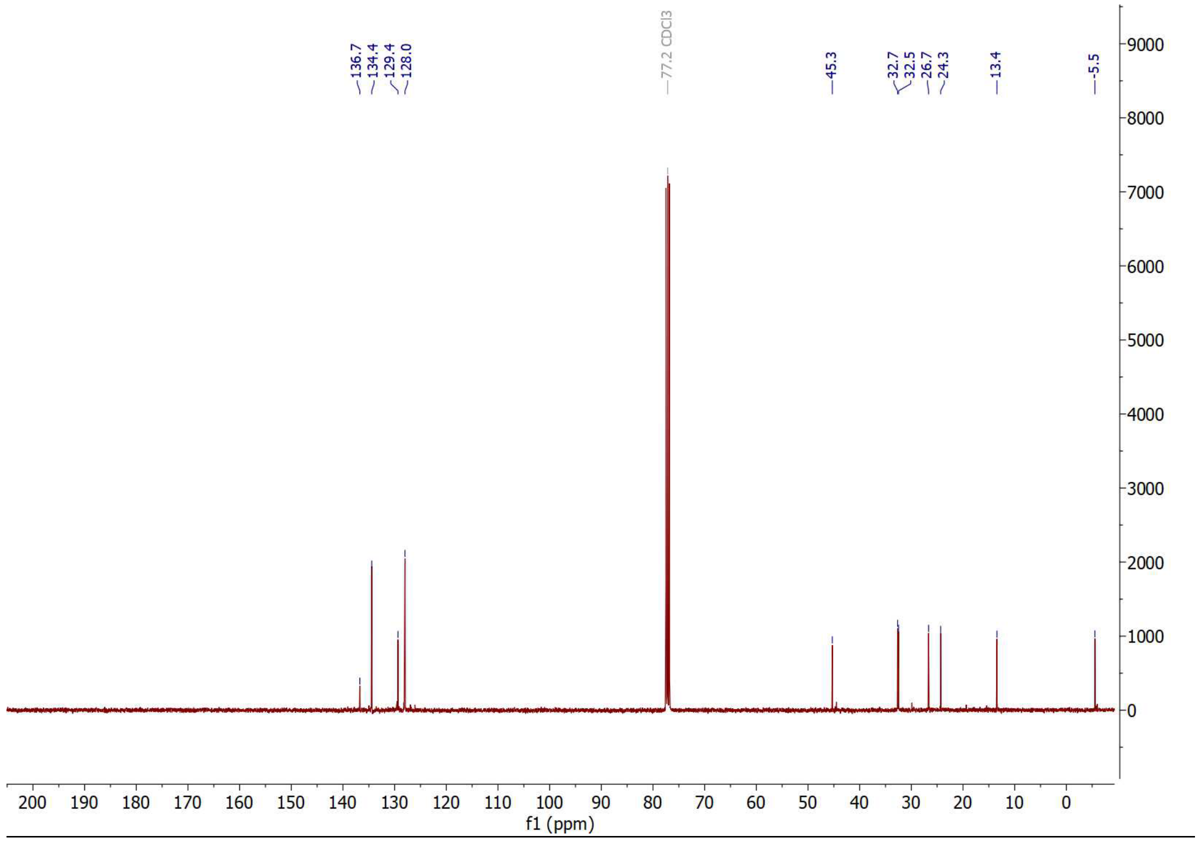


**Figure S47.** $^{13}C\{^1H\}$-NMR spectrum of **2n**

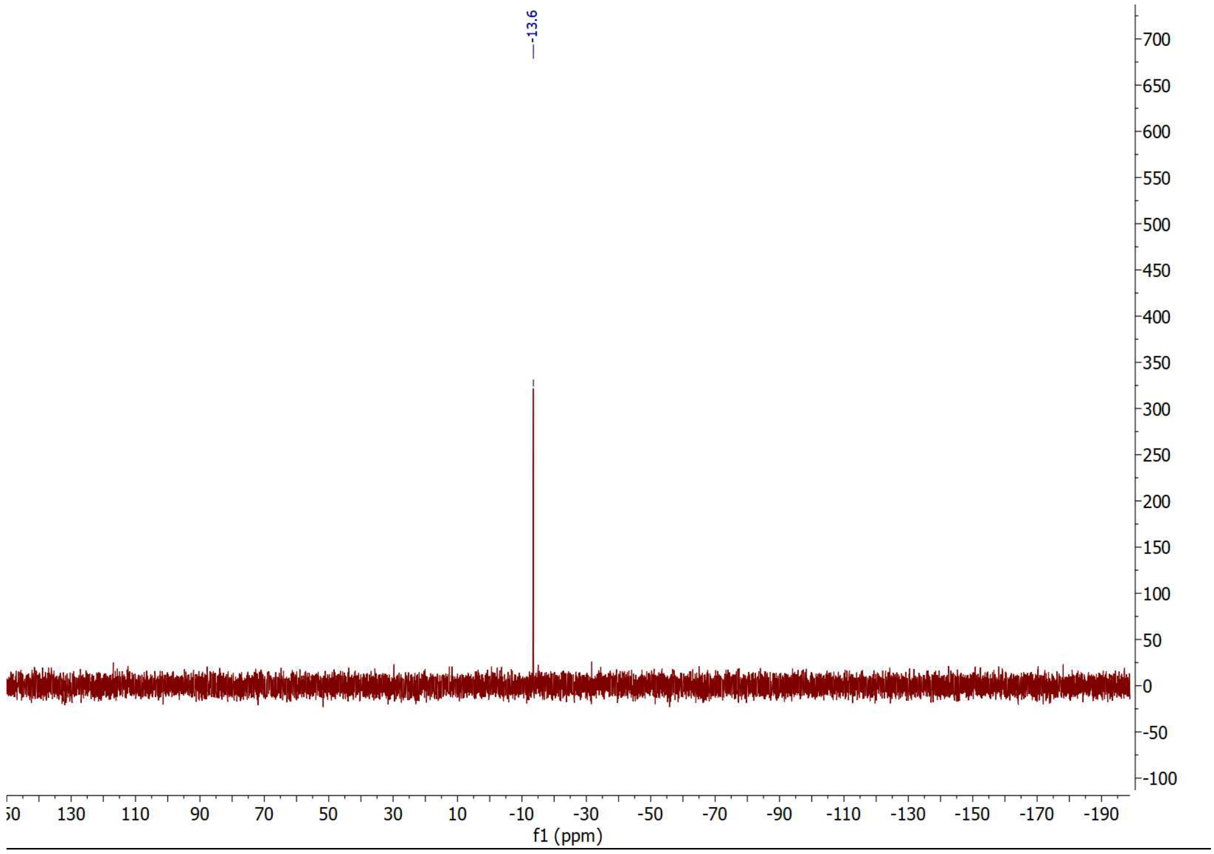


**Figure S48.** $^{29}Si\{^1H\}$-NMR spectrum of **2n**

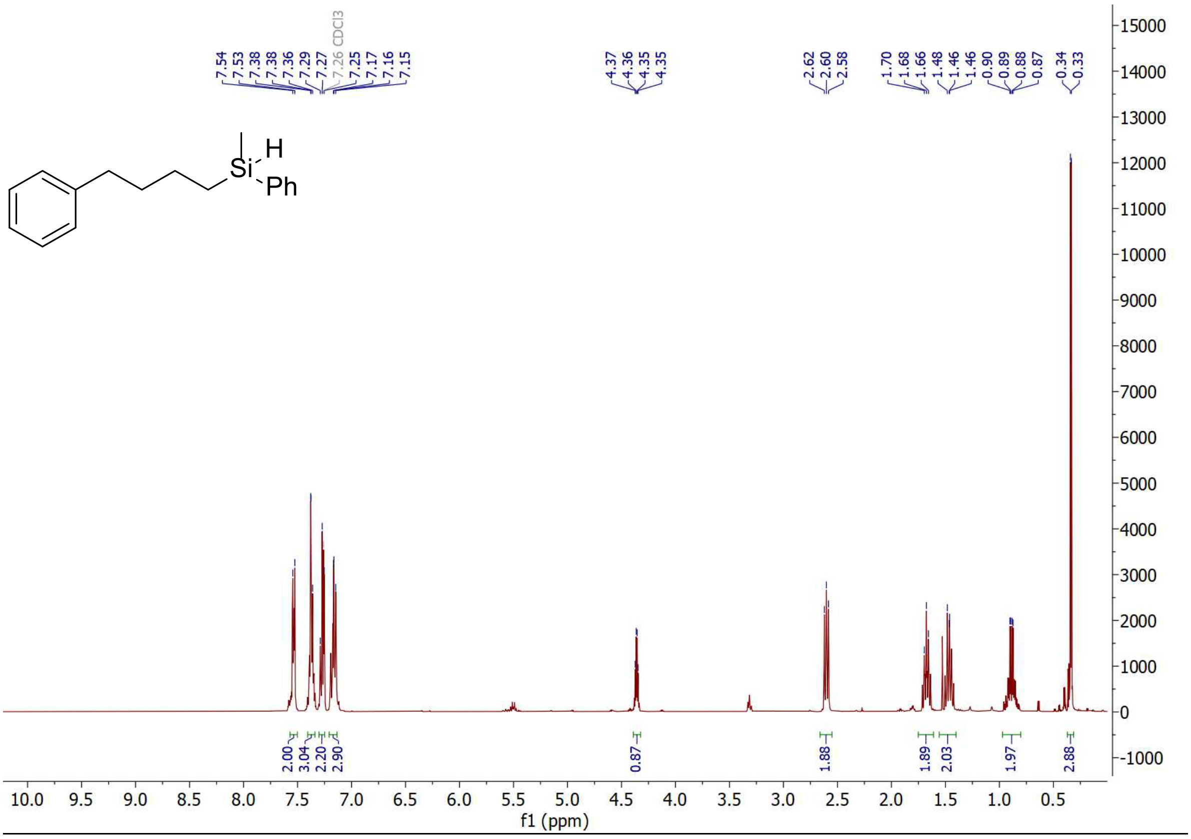


**Figure S49.** $^{1}$H-NMR spectrum of **2o**

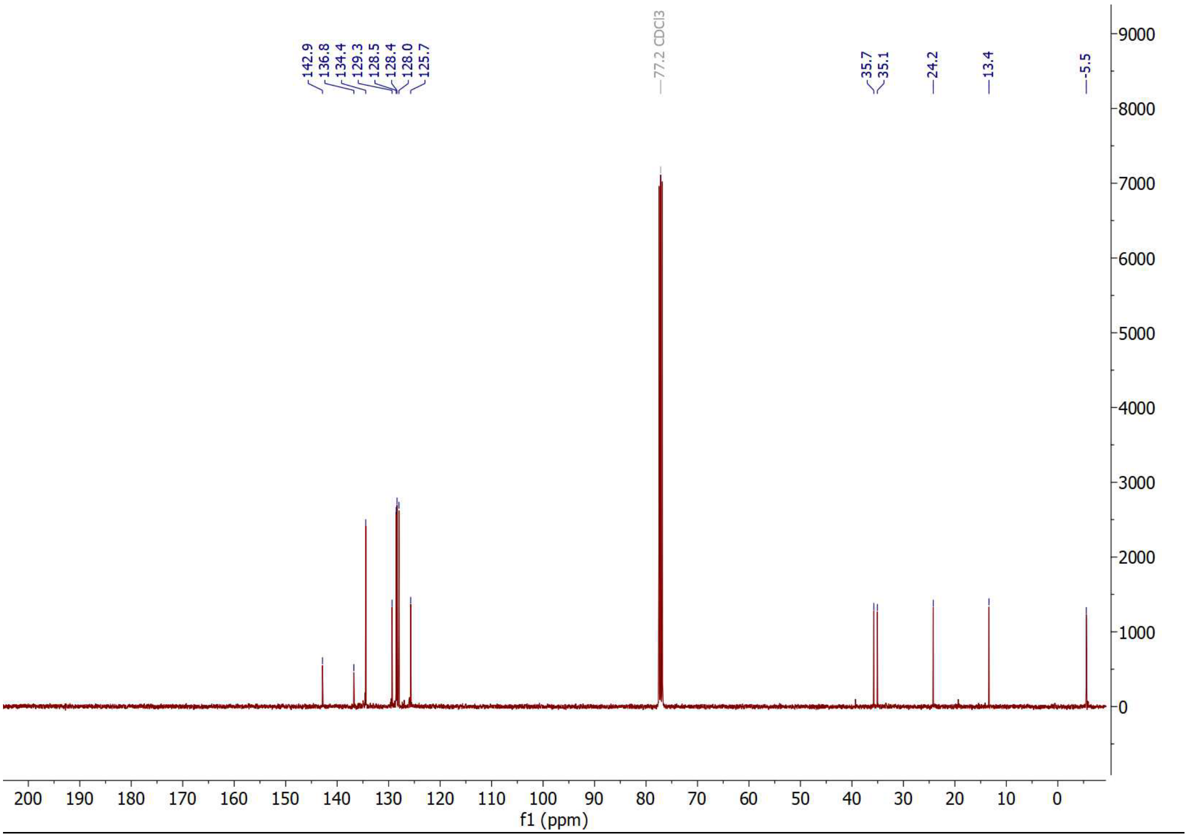


**Figure S50.** $^{13}$C{$^{1}$H}-NMR spectrum of **2o**

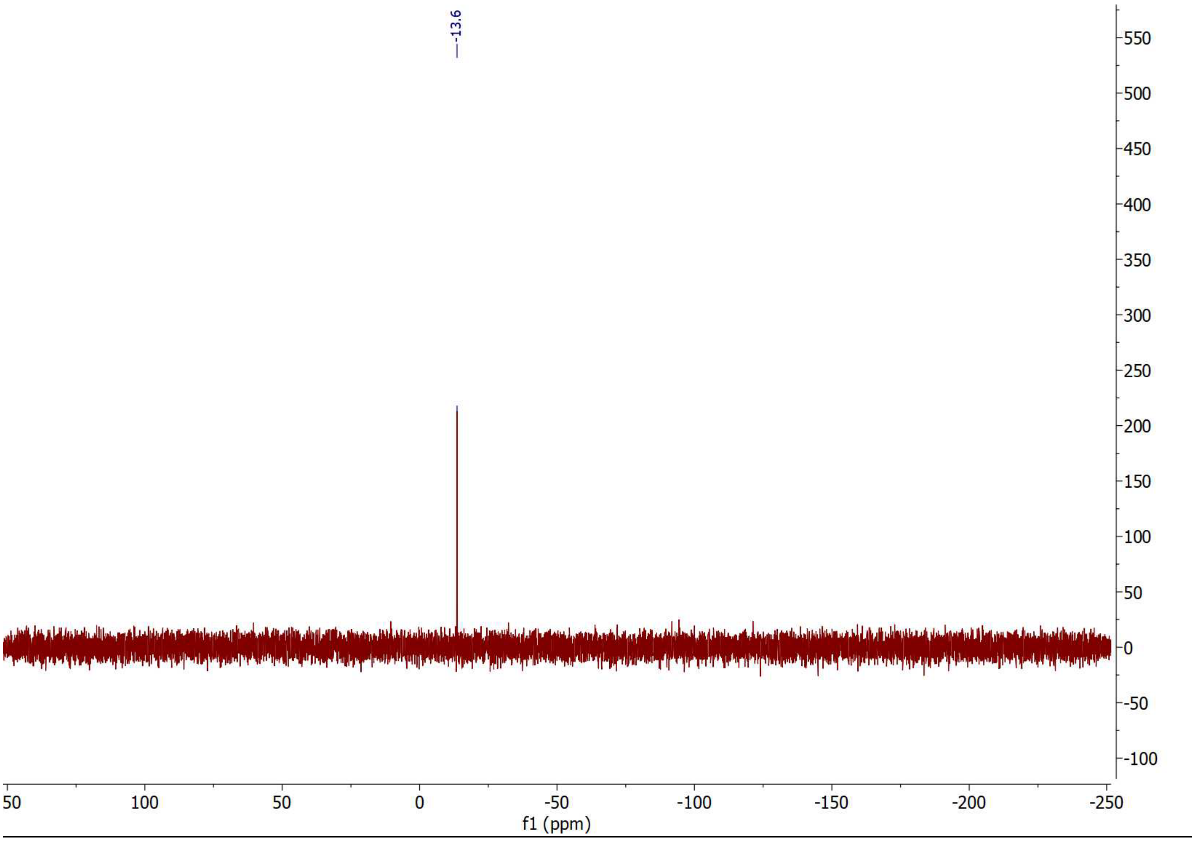


**Figure S51.** $^{29}Si\{^{1}H\}$-NMR spectrum of **2o**

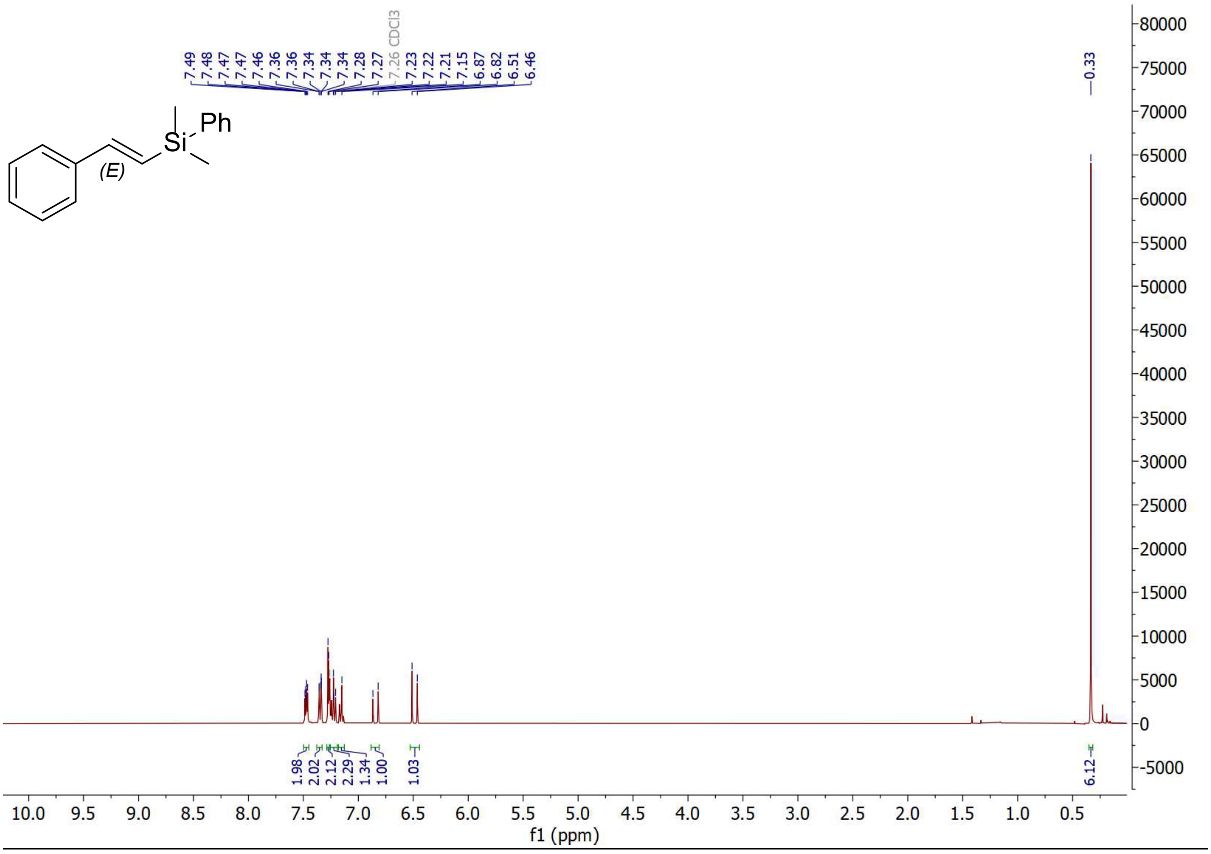


**Figure S52.** $^{1}H$-NMR spectrum of **3a**

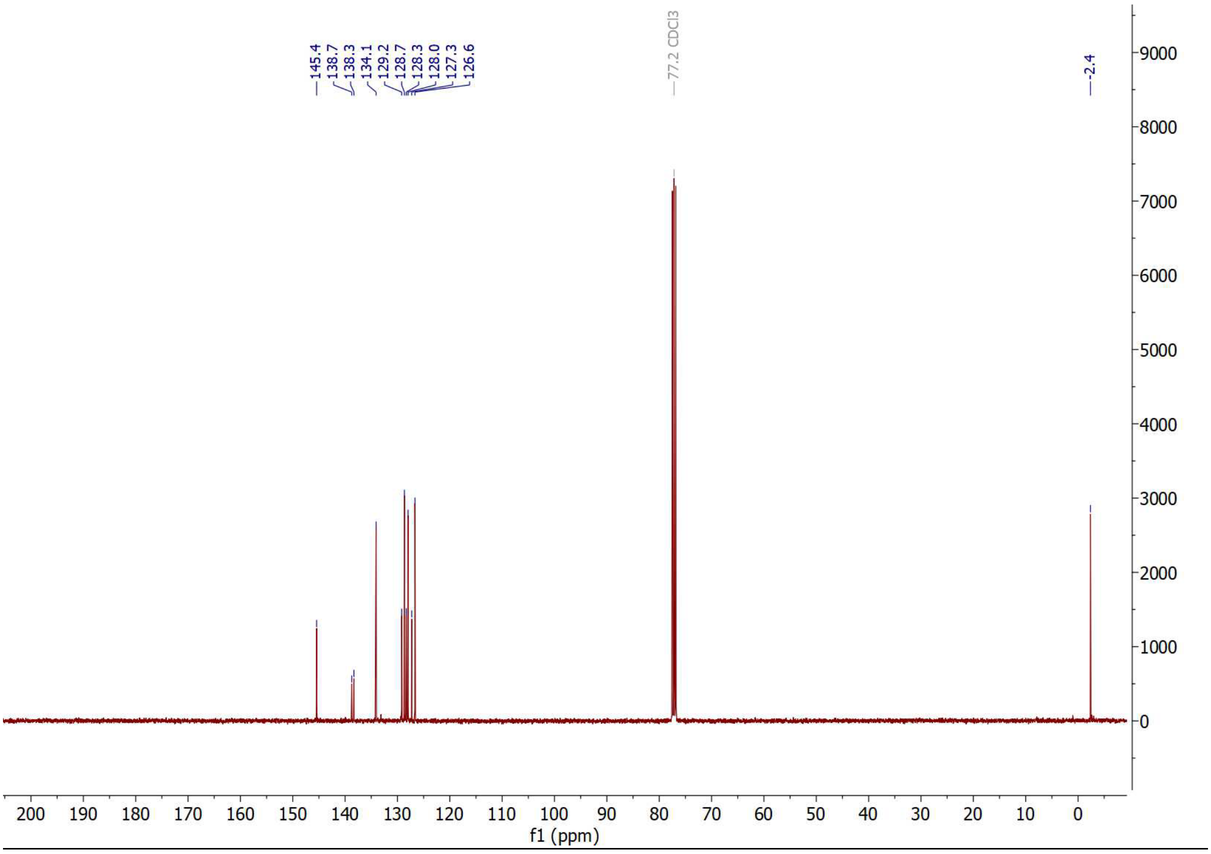


**Figure S53.** $^{13}C\{^1H\}$-NMR spectrum of **3a**

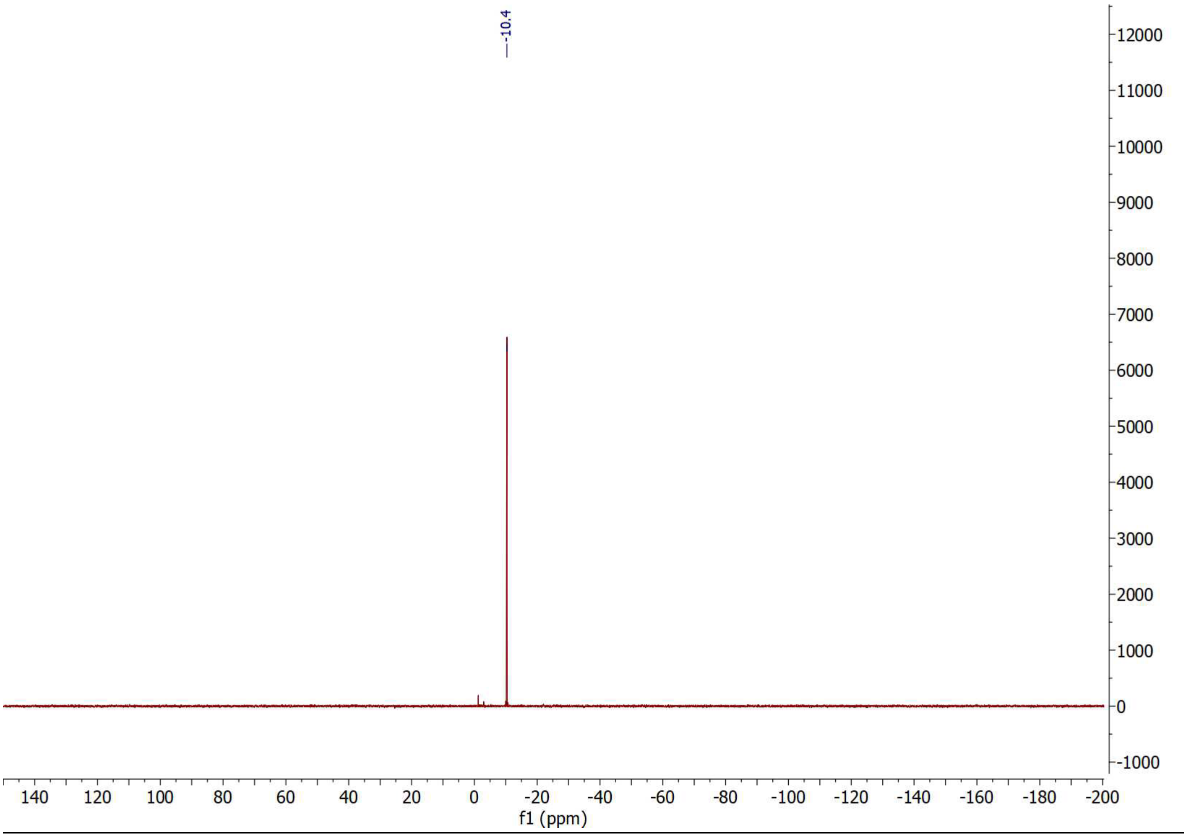


**Figure S54.** $^{29}Si\{^1H\}$-NMR spectrum of **3a**

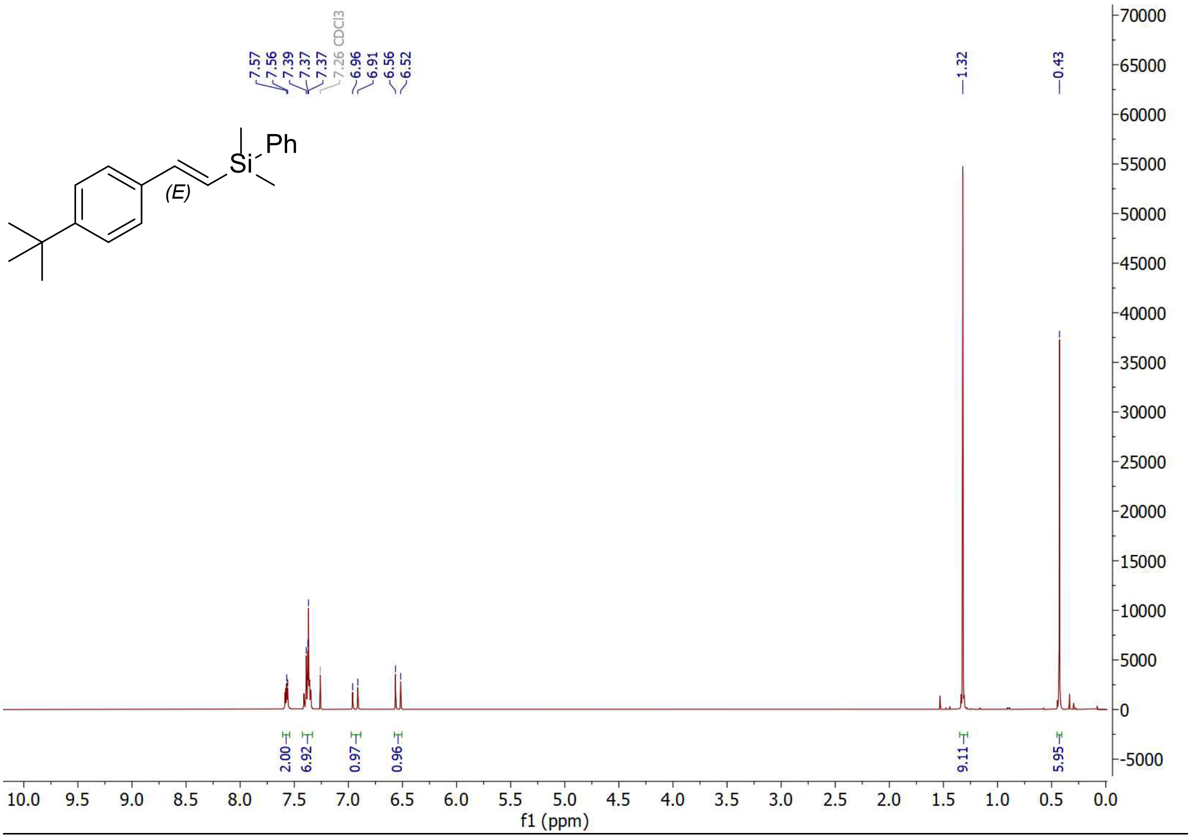


**Figure S55.** $^{1}$H-NMR spectrum of **3b**

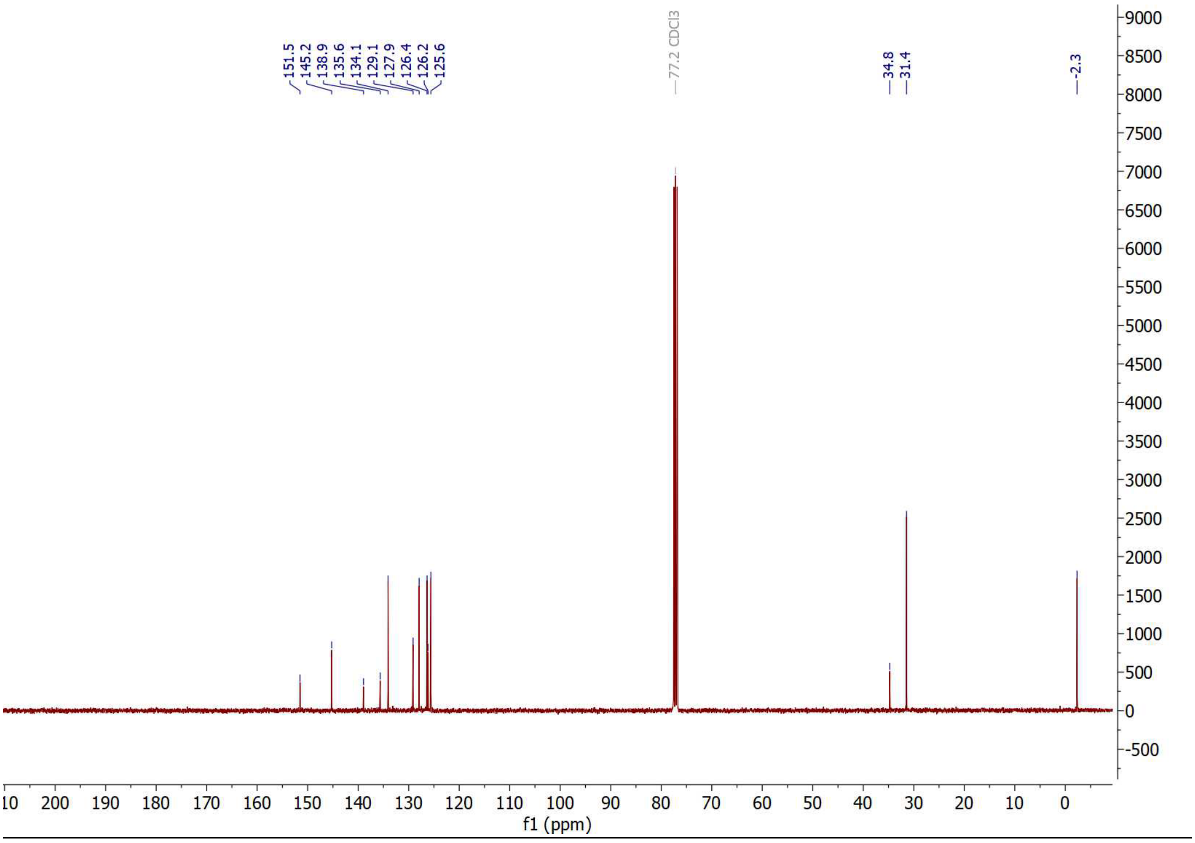


**Figure S56.** $^{13}C\{^{1}H\}$-NMR spectrum of **3b**

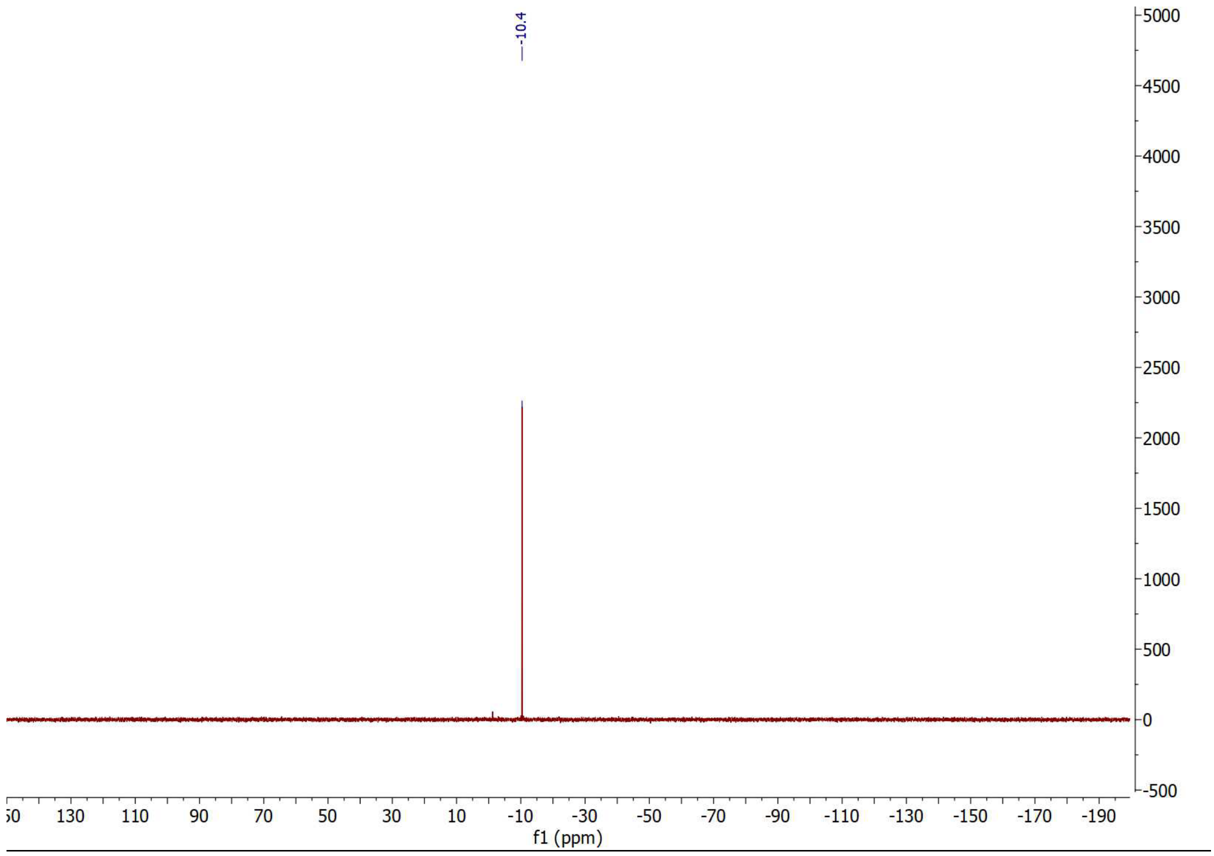

**Figure S57.** $^{29}Si\{^1H\}$-NMR spectrum of **3b**

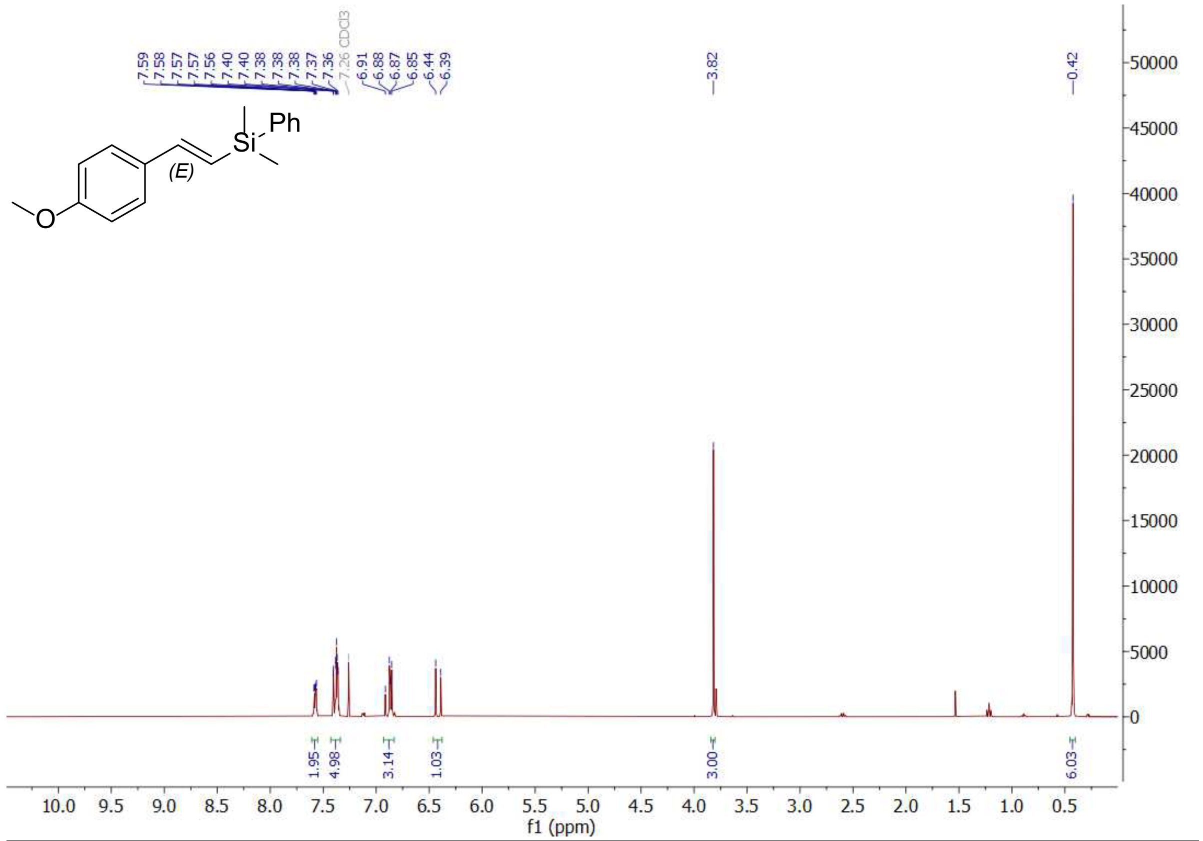

**Figure S58.** $^1H$-NMR spectrum of **3c**

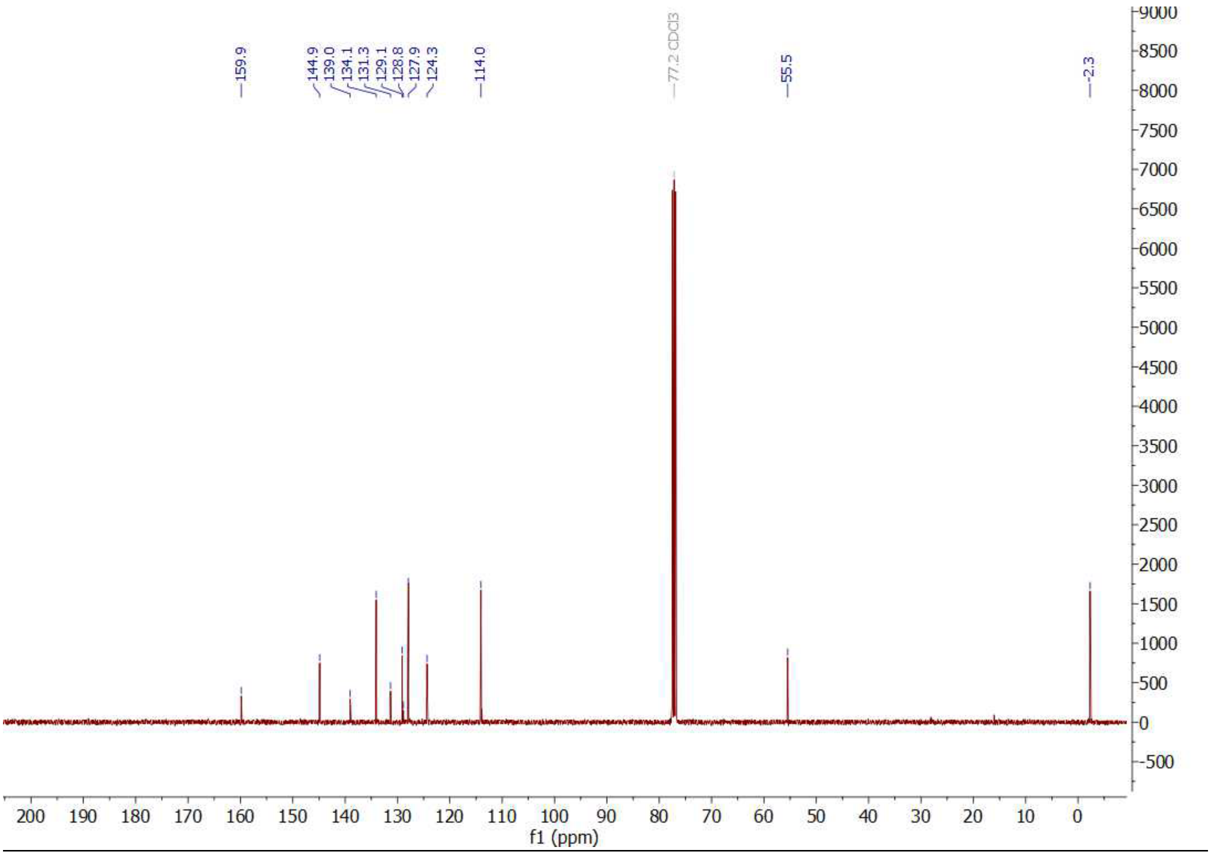


**Figure S59.** $^{13}C\{^{1}H\}$-NMR spectrum of **3c**

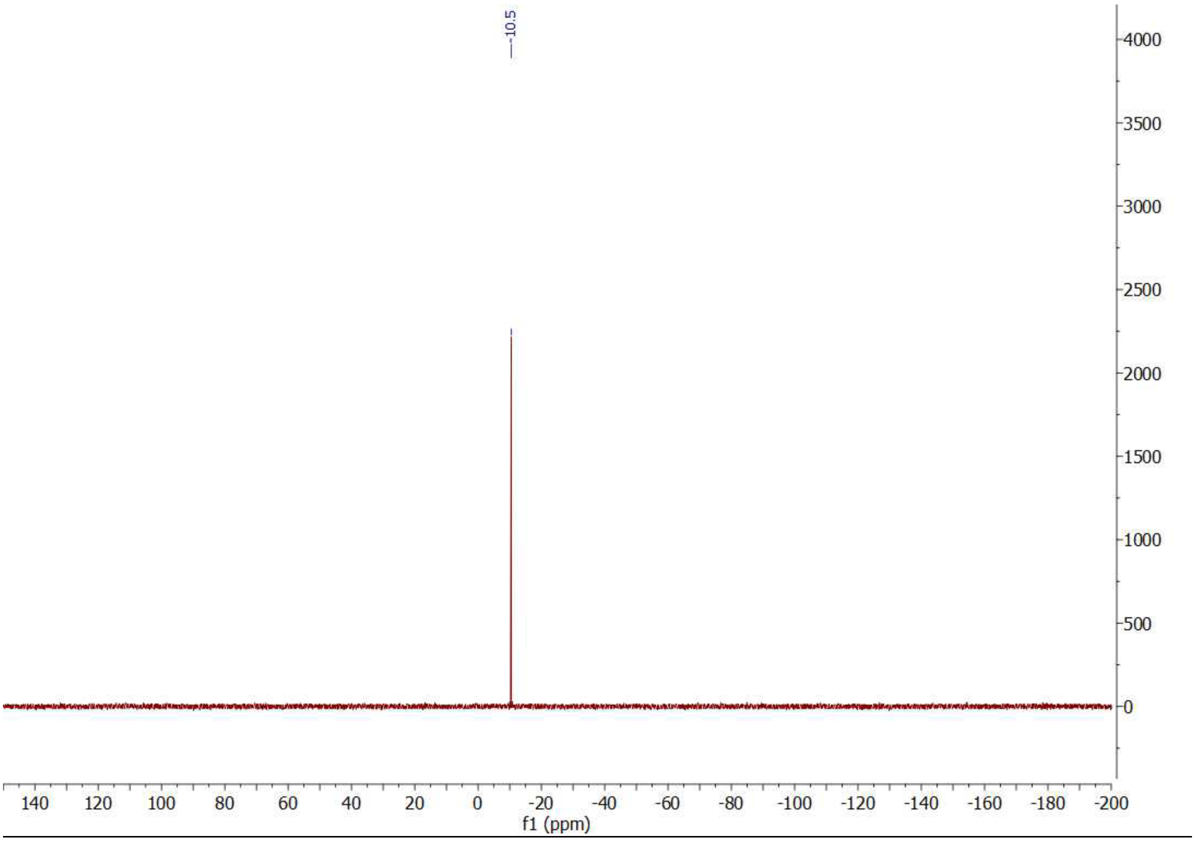


**Figure S60.** $^{29}Si\{^{1}H\}$-NMR spectrum of **3c**

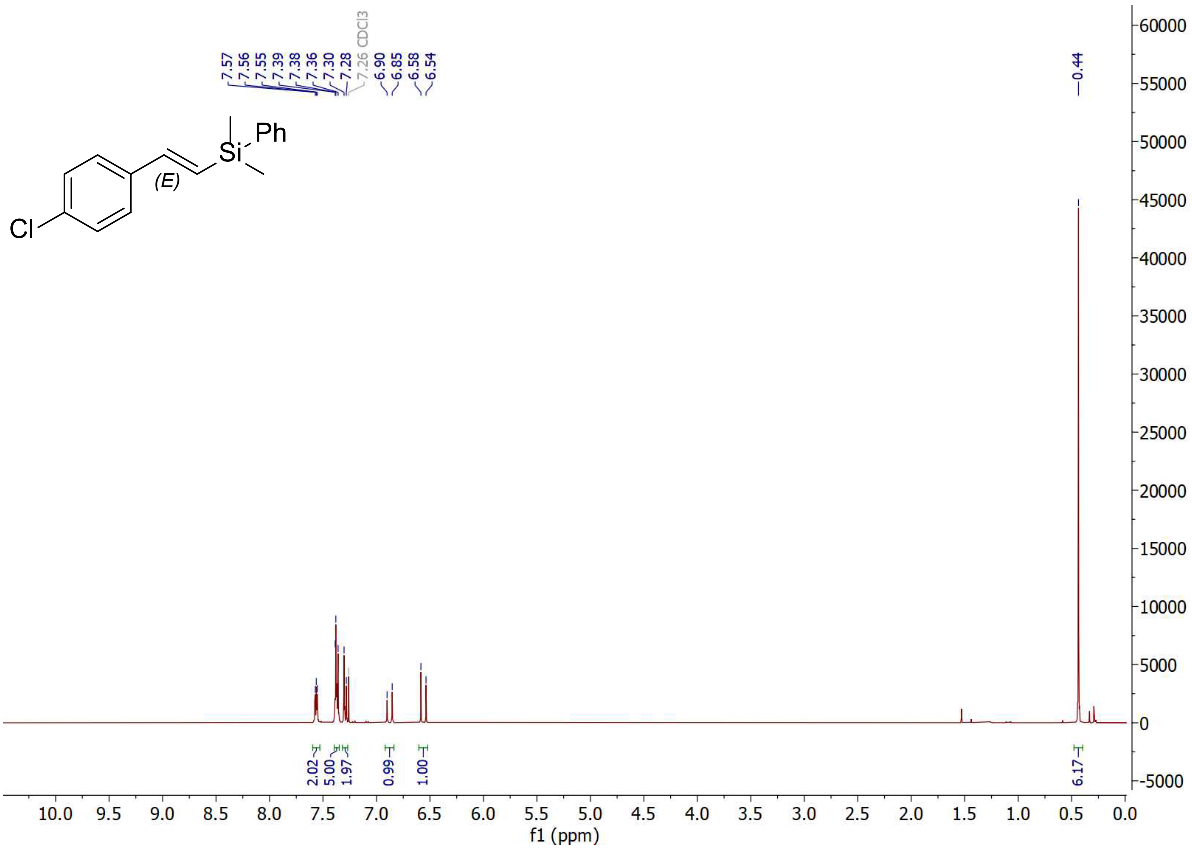


**Figure S61.** $^{1}$H-NMR spectrum of **3d**

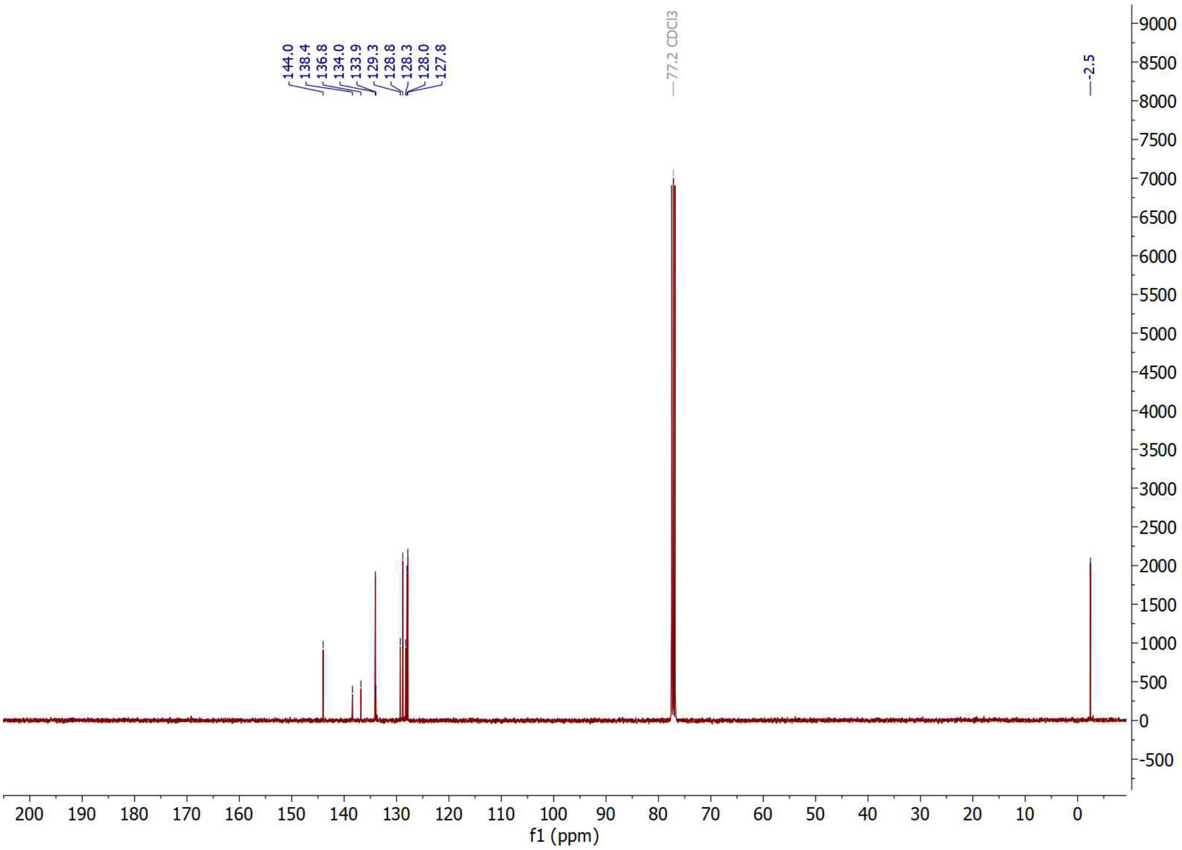


**Figure S62.** $^{13}C\{^{1}H\}$-NMR spectrum of **3d**

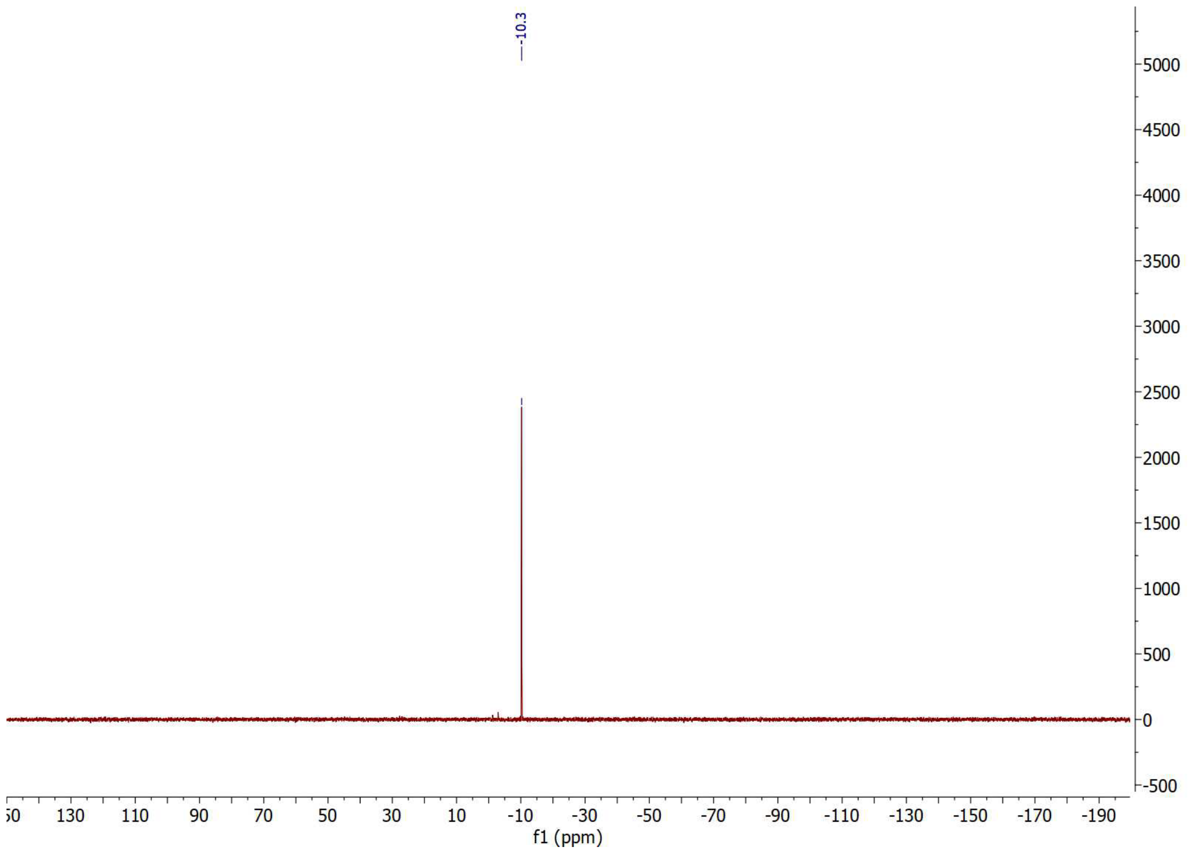


**Figure S63.** $^{29}Si\{^1H\}$-NMR spectrum of **3d**

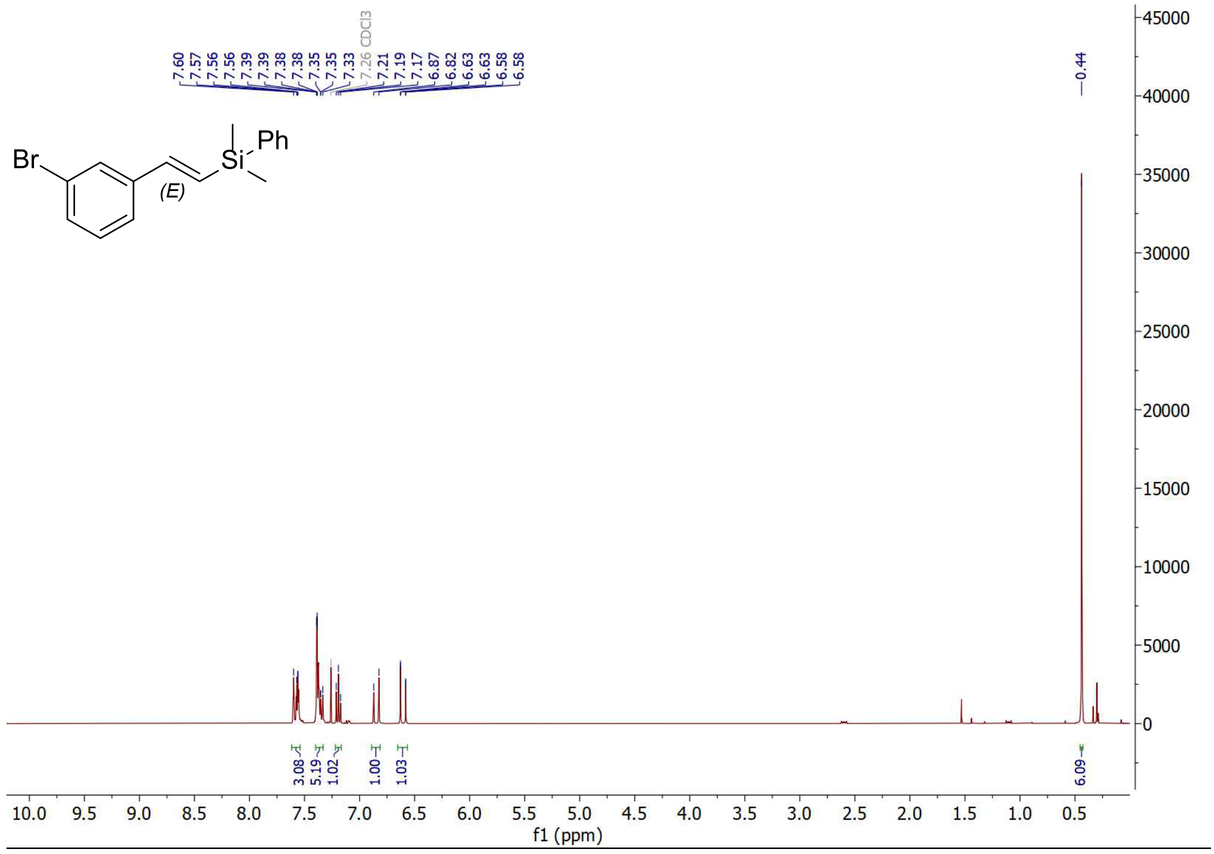


**Figure S64.** $^1H$-NMR spectrum of **3e**

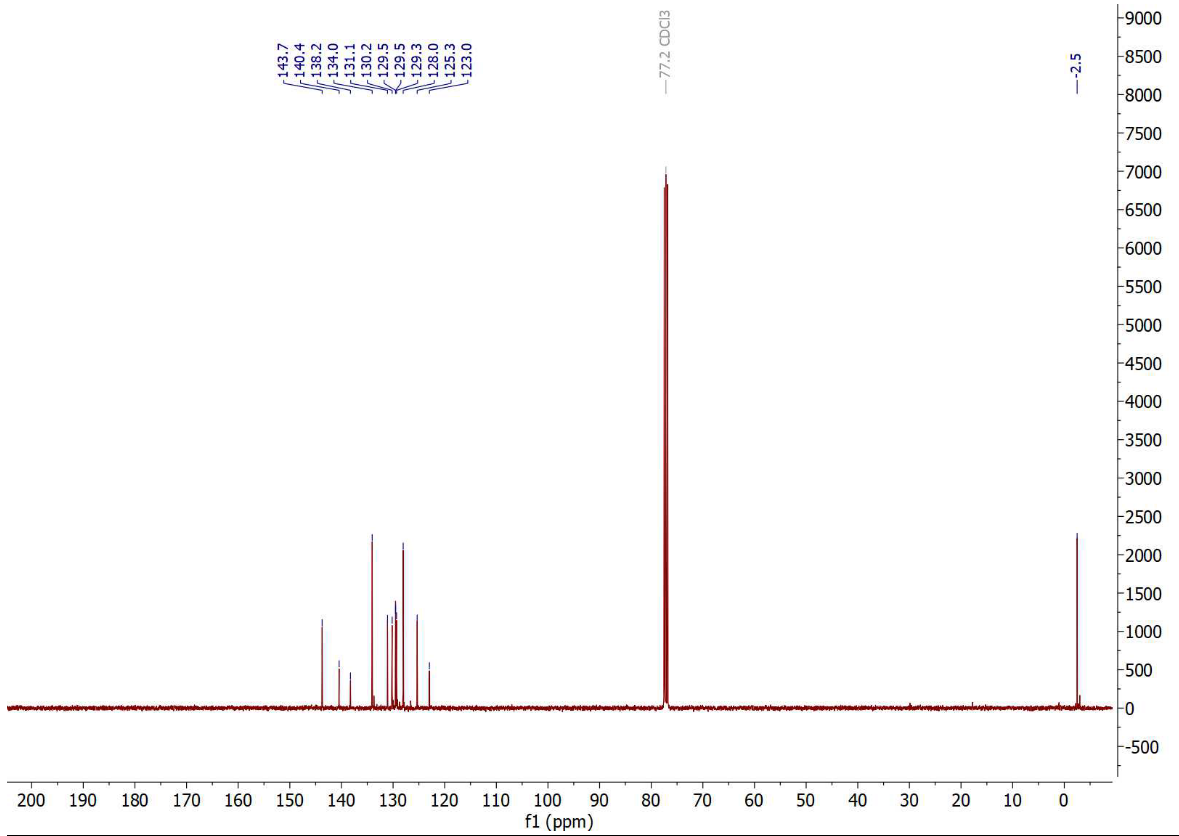


**Figure S65.** $^{13}C\{^{1}H\}$-NMR spectrum of **3e**

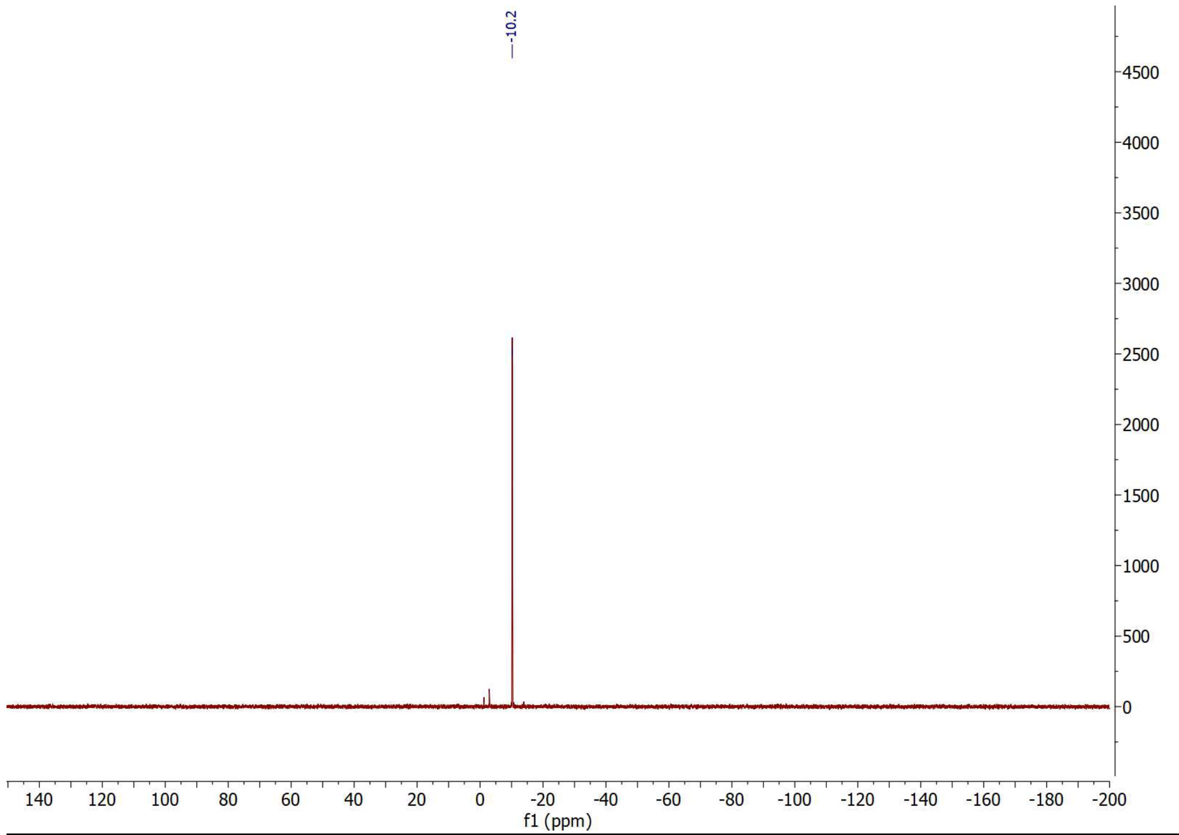


**Figure S66.** $^{29}Si\{^{1}H\}$-NMR spectrum of **3e**

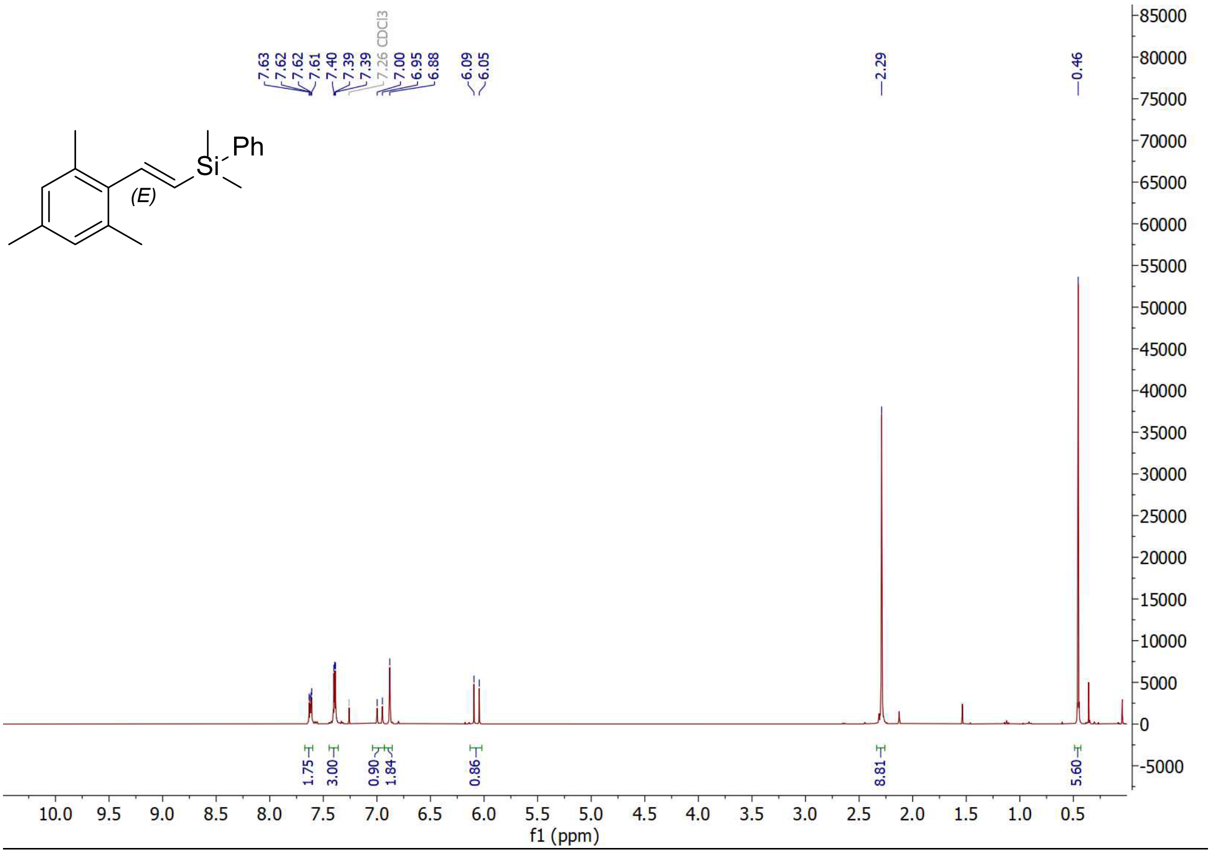


**Figure S67.** $^{1}$H-NMR spectrum of **3f**

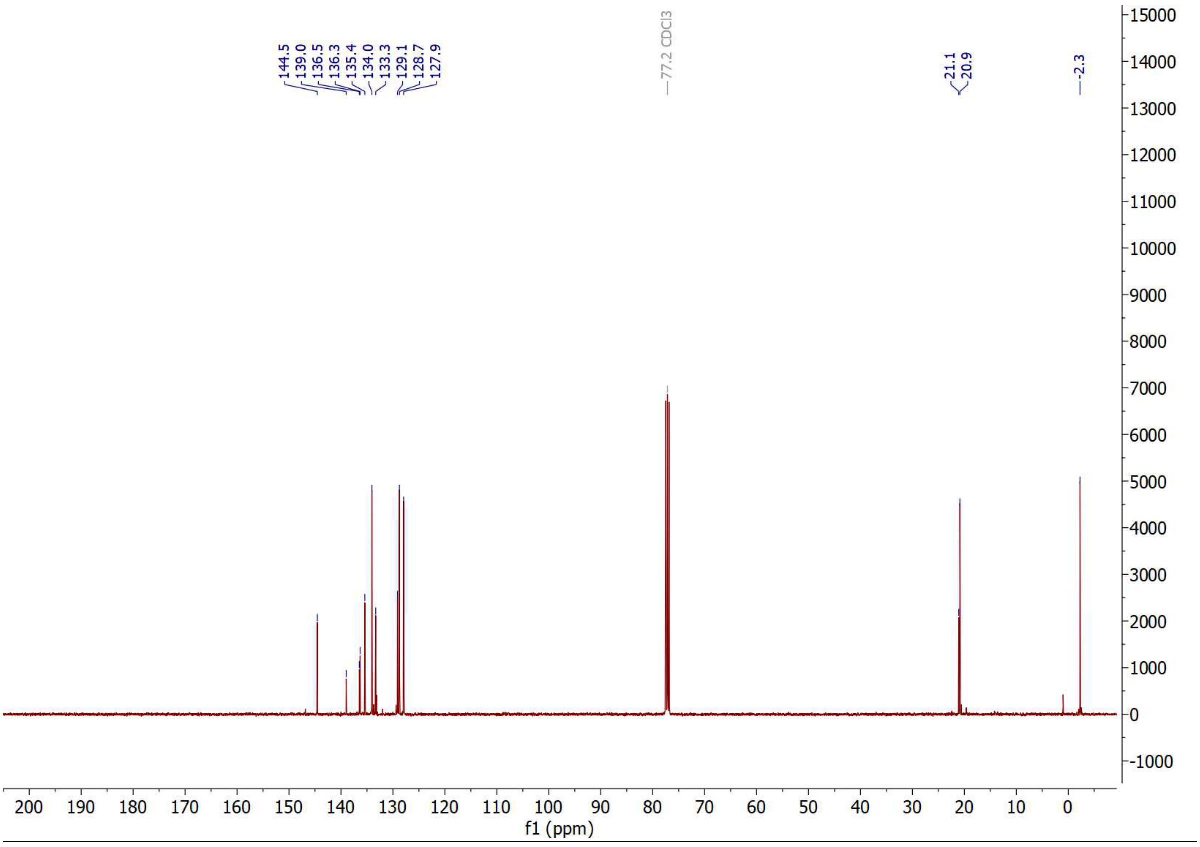


**Figure S68.** $^{13}C\{^{1}H\}$-NMR spectrum of **3f**

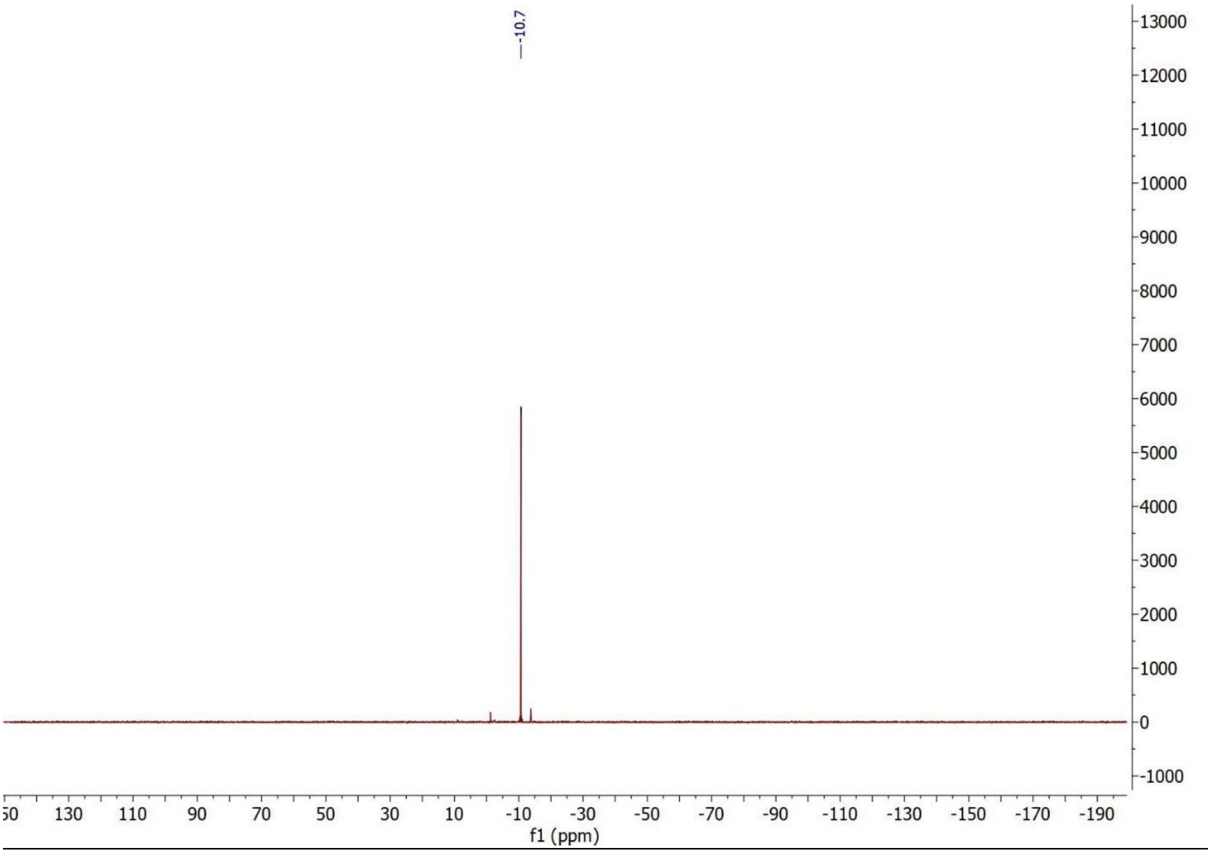


**Figure S69.** $^{29}Si\{^{1}H\}$-NMR spectrum of **3f**
